# Stereoselective Synthesis of Oxazolidin-2-ones via an Asymmetric Aldol/Curtius Reaction: Concise Total Synthesis of (−)-Cytoxazone

**DOI:** 10.3390/molecules26030597

**Published:** 2021-01-23

**Authors:** Hosam Choi, Hanho Jang, Joohee Choi, Kiyoun Lee

**Affiliations:** Department of Chemistry, The Catholic University of Korea, Bucheon 14662, Korea; pzpz1233@gmail.com (H.C.); gksghwkd0925@gmail.com (H.J.); cholo4015@naver.com (J.C.)

**Keywords:** oxazolidin-2-one, (−)-cytoxazone, natural product, total synthesis, curtius reaction, asymmetric aldol

## Abstract

Herein, we are reporting an efficient approach toward the synthesis of 4,5-disubstituted oxazolidin-2-one scaffolds. The developed approach is based on a combination of an asymmetric aldol and a modified Curtius protocol, which uses an effective intramolecular ring closure to rapidly access a range of oxazolidin-2-one building blocks. This strategy also permits a straightforward and concise asymmetric total synthesis of (−)-cytoxazone. Consisting of three steps, this is one of the shortest syntheses reported to date. Ultimately, this convenient platform would provide a promising method for the early phases of drug discovery.

## 1. Introduction

**Scheme 1:**
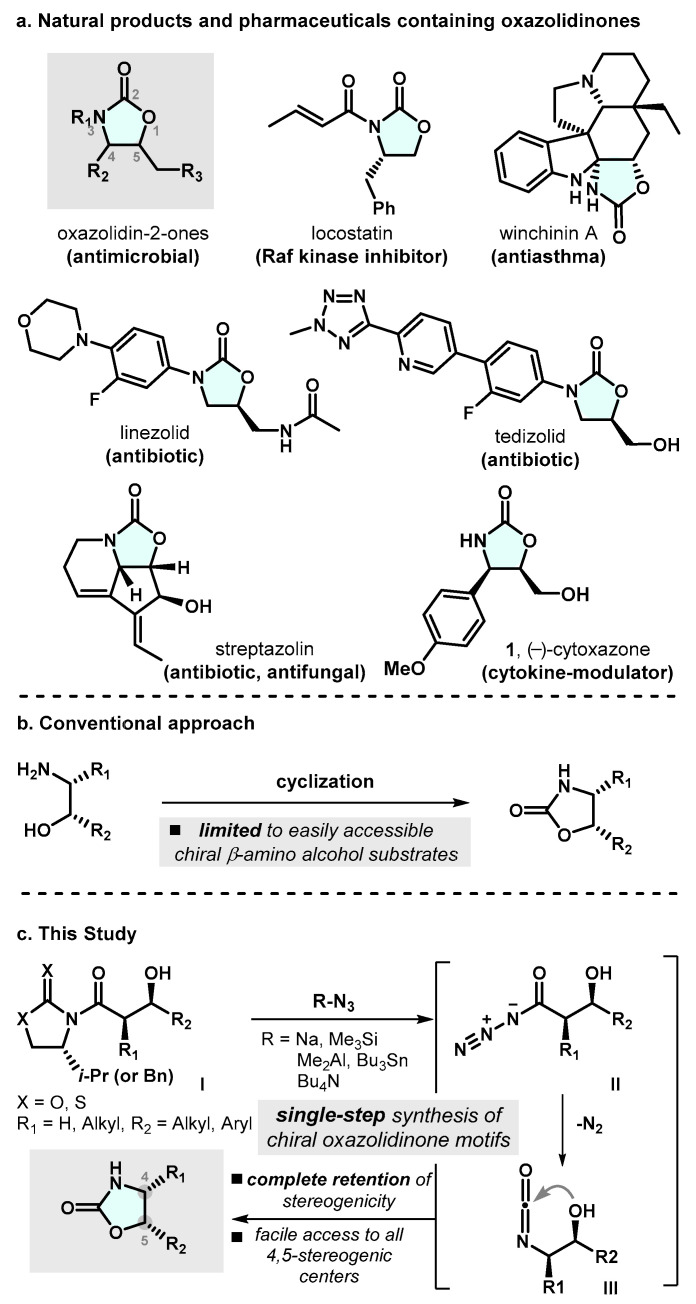
(**a**) Natural products and pharmaceuticals containing oxazolidin-2-ones (**b**) Conventional method towards the oxazolidin-2-ones (**c**) Proposed Strategy for the synthesis of the 4,5-disubstituted oxazolin-2-one framework.

Functionalized oxazolidin-2-ones are among the most interesting heterocyclic compounds, with uses both as pharmaceuticals and as key synthetic intermediates (Scheme 1a). Since the seminal discovery of anti-bacterial activity of certain oxazolidin-2-ones by EI DuPont de Nemours and Co., Inc. in 1987 [1], the synthesis of substituted oxazolidin-2-one motifs has drawn a considerable amount of interest from the synthetic community [2,3]. These functionalized oxazolidin-2-one compounds have demonstrated a wide spectrum of pharmacological properties [4,5,6]. For instance, linezolid (LZD), the first synthetic oxazolidin-2-one antimicrobial agent, has shown potent efficacy against Gram-positive bacteria through the inhibition of bacterial protein synthesis [7,8]. LZD was approved by the FDA for the treatment of a range of traditionally drug-resistant infections, including MRSA and drug-resistant tuberculosis in 2002 [9]. Tedizolid is also a promising antibacterial agent that is used to treat skin infection in adults [10]. Thanks for these pharmacological properties, structurally diverse oxazolidin-2-one motifs often serve as key synthetic intermediates within the context of macrolide antibiotics syntheses. Additionally, these cyclic carbamate moieties are one of the most significant and widely utilized chiral auxiliaries in the realm of organic synthesis [11,12,13].

Due to the amount of intrigue surrounding the unique structural features and diverse therapeutic utility, in the past decades, great emphasis has been placed on the development of synthetic approaches toward the enantioselective construction of oxazolidin-2-one moieties. There are several conventional approaches for the construction of oxazolidin-2-ones. One of the most common methods involves the intermolecular reaction of a *β*-amino alcohol with phosgene (Scheme 1b) [14,15].

However, these types of method suffer from limited availability of starting materials, in some cases, due to the need for a pre-installed stereogenic center on the β-amino alcohol prior to ring closure. Alternative common strategies include the reaction of isocyanates with epoxides and the reaction of aziridines with carbon dioxide [16,17,18]. Despite these advances in the field of oxazolidin-2-one synthesis, successful methods are thus far largely limited in terms of the access to substrates, regio- and stereochemical outcome, ability to increase molecular complexity of easily obtainable starting materials, and sufficiently mild reaction conditions that are compatible with various functional groups. Furthermore, to date, the asymmetric synthesis of oxazolidin-2-one motifs has thus far remained out of reach. More specifically, many asymmetric strategies reported to have focused on the generation of C4- or C5-monosubstituted oxazolidinones, while strategies providing access to oxazolidinones possessing 4,5-two vicinal stereogenic centers have rarely been reported. These aspects have resulted in the continuous demand for new synthetic approaches for the construction of optically active oxazolidin-2-one moieties.

To meet this synthetic need, herein we disclose studies on the development of an efficient synthesis of oxazolidin-2-one scaffolds with access to 4,5-vicinal stereogenic centers, which uses a combination of an asymmetric aldol and a modified Curtius procedure to undergo an effective intramolecular cyclization. This convenient platform consequently permitted the concise total synthesis of natural (−)-cytoxazone (**1**) in only three steps. To the best of our knowledge, this work presents the shortest asymmetric total synthesis of (−)-cytoxazone reported to date.

The Curtius approach most commonly involves the isolation of acyl azides from carboxylic acid derivatives such as acyl chlorides or mixed anhydrides [19,20,21]. However, the acid chloride itself often raises the issue of compatibility with acid-labile functionalities and has additional drawbacks such as issues with preparation and storage. Alternative one-pot protocols for the synthesis of carbarmates from carboxylic acids by employing the diphenylphosphoryl azide (DPPA) have been more attractive [22,23]. Though quite efficient, toxicity issues and purification difficulties have been inescapable, resulting in restriction of scope and consequently, synthetic utility.

## 2. Results and Discussion

Keeping these considerations in mind, we were interested in the direct conversion of chiral imides to cyclic carbamates without the isolation of highly reactive acyl chlorides and/or unstable acyl azides. As illustrated in Scheme 1c, we envisaged that efficient, direct, and even diastereoselective construction of an optically active 4,5-disubstituted oxazolidin-2-one motif would be possible if a chiral auxiliary-mediated asymmetric aldol reaction was utilized in conjunction with the Curtius rearrangement, followed by in situ intramolecular ring closure in a tandem manner.

In terms of the simplified reaction mechanism, upon treatment with sufficiently nucleophilic azide transfer reagents, the chiral auxiliary bearing starting material **I** could be converted into acyl azide intermediate **II**. Subsequent thermal decomposition results in the formation of an acyl nitrene with the loss of N_2_, followed by the molecular rearrangement to generate the desired chiral oxazolidin-2-one, presumably through the intramolecular ring closure of the isocyanate intermediate **III**.

**Scheme 2:**
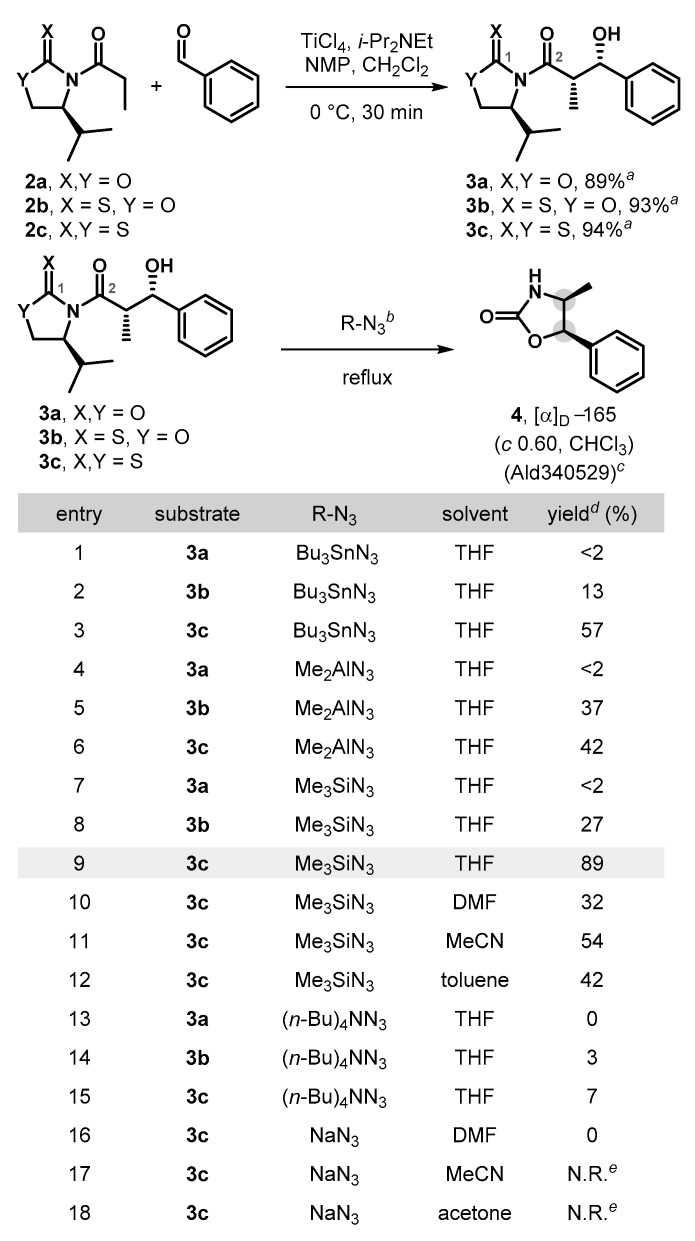
Synthesis of oxazolidin-2-one via the azidation/Curtius rearrangement sequence. *^a^* Isolated yield of major diastereomer. *^b^* Reaction conditions: 0.05–0.2 mmol of **3a**–**c**. Bu_3_SnN_3_ (3 equiv., 30 min, entries 1–3), Me_2_AlN_3_ (3 equiv., 5 min, entries 4–6), Me_3_SiN_3_ (3 equiv., 3–5 h, entries 7–12), (*n*-Bu)_4_NN_3_ (3 equiv., 2 h, entries 13–15), NaN_3_ (10 equiv., 5 h, entries 16–18). *^c^* Commercially available. *^d^* Isolated yield. *^e^* No reaction was observed.

In order to test this hypothesis, *syn*-aldol adduct **3a** was prepared as a model substrate by employing an Evans type auxiliary-mediated aldol protocol [11,24,25,26] following the procedure described by Crimmins [27,28,29] (Scheme 2). Initially, we attempted the direct nucleophilic azidation/Curtius reaction by treatment of **3a** with Bu_3_SnN_3_ (3 equiv., THF, 90 °C). To our disappointment, the reaction only produced a trace amount (less than 2%) of product **4** (entry 1). Introducing an oxazolidinethione in *syn*-aldol product **3b** and ensuing subjection in the nucleophilic azide transfer/Curtius reaction improved the yield, but only to 13% (entry 2).

These low conversions could be attributed to the inherent electrophilic nature of C1 carbonyl carbon in **3a**–**c** and the associated inhibition of the initial acyl azide formation. Therefore, we envisioned that corresponding aldol precursors with diminished electrophilicity at C1 in auxiliaries would potentially be more productive than oxazolidinones or oxazolidinethiones in establishing more effective construction of the oxazolidin-2-ones [29,30]. Consistent with our expectation, the nucleophilic azidation/Curtius reaction of thiazolidinethione **3c** under the same reaction conditions (Bu_3_SnN_3_, THF, 90 °C) proceeded to provide corresponding oxazolidin-2-one **4** in higher conversion (57%).

Encouraged by this promising result, we contemplated a series of alternative chiral auxiliaries and several N_3_ transfer reagents and ultimately found that a combination of the use of asymmetric *syn*-aldol products bearing *N*-acylthiazolidinethione with a Bn or an *i*-Pr moiety and treatment with Me_3_SiN_3_ [31] (3 equiv., THF, 90 °C, entry 9) proved to be highly effective and cleanly afforded the corresponding oxazolidin-2-one **4** in superb conversion (89%). Optimization of the solvent system revealed that THF was the best solvent of those examined (entries 9–12). The use of higher reaction concentrations (0.2 M) or prolonged reaction times (12 h) did not further improve the already excellent yield. It is worth mentioning that the developed protocol proceeds with complete retention of the enantiomeric excess and the diastereoselectivity in **4** (see Supplementary Material). Alternative nucleophilic azide transfer reactions (M_2_Al-N_3_/THF or (*n*-Bu)_4_NN_3_/THF) were also successful, but afforded **4** in lower conversions (Scheme 2, entries 4–6, 13–15). However, reactions that employed NaN_3_ proved ineffective in all tested solvent systems (DMF, MeCN, and acetone) (entries 16–18).

**Scheme 3:**
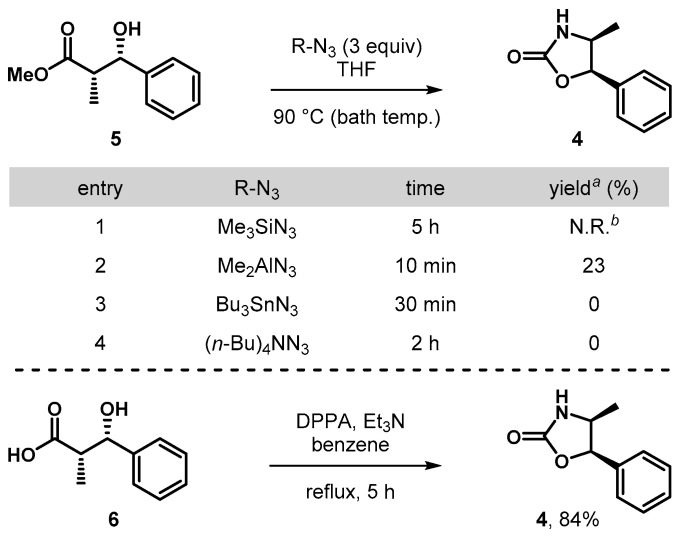
Synthesis of 4,5-disubstituted oxazolidin-2-ones from **5** and **6**. *^a^* Isolated yield. *^b^* No reaction was observed. DPPA = diphenyl phosphoryl azide.

To gain further insight into the effect of thiazolidinethione moiety in compound **3c**, we next sought to address the reactivity with a simpler ester functionality (Scheme 3). To this end, methyl ester **5** was prepared by methanolysis of **3c** (DMAP (20 mol%), MeOH (1.5 equiv.), CH_2_Cl_2_, 25 °C) and subsequent azidation/Curtius reaction in the presence of Me_2_AlN_3_ gave the oxazolidin-2-one **4** in low yield (23%). Surprisingly, the azidation/Curtius reactions of **5** with the treatment of Me_3_SiN_3_, Bu_3_SnN_3_, or (*n*-Bu)_4_NN_3_ failed to provide the corresponding **4**, but rather only resulted in no reactivity for the former and significant decomposition for the latter two cases. Subsequently, carboxylic acid **6** was also evaluated by the treatment of DPPA (Et_3_N, benzene, reflux), which proceeded to provide **4** in 84%, suggesting that a direct mode of oxazolidin-2-one formation from a substrate such as **3c** may constitute a more effective approach than a multi-step process.

**Scheme 4:**
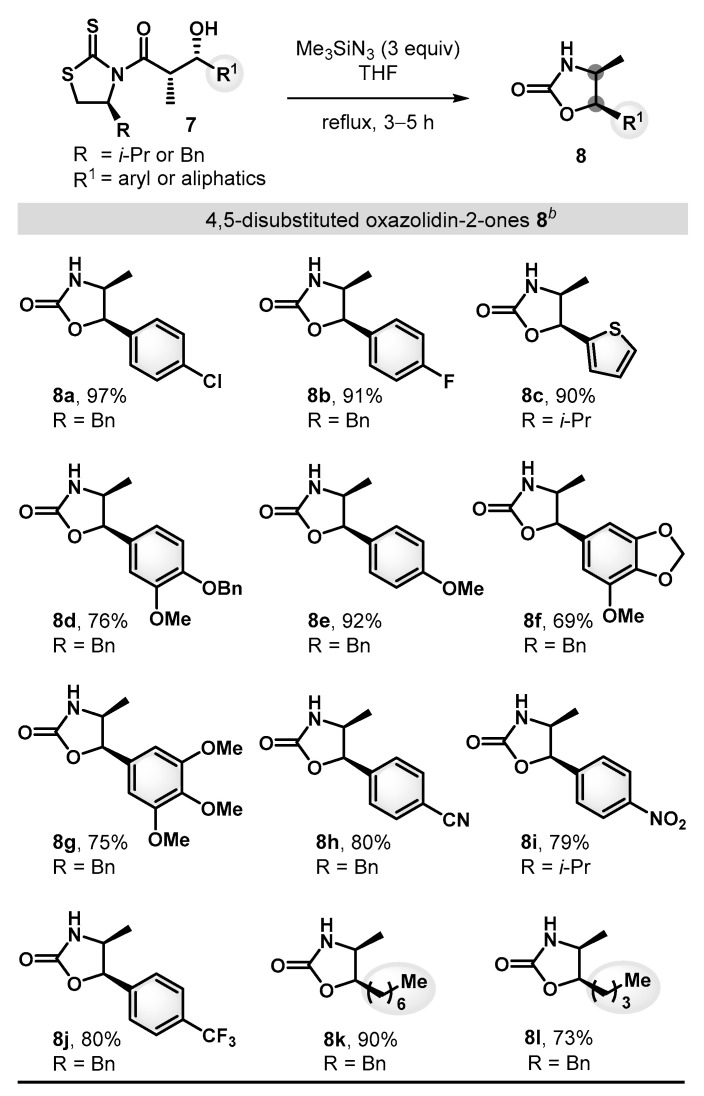
Substrate scope of the synthesis of 4,5-disubstituted oxazolidin-2-ones *^a^. ^a^* All reactions were run on 0.1–0.2 mmol scale under the standard conditions. *^b^* Yields of isolated products after purification by flash chromatography.

Having achieved optimal reaction conditions, we set out to explore the scope and the limitations of this transformation (Scheme 4). A series of *β*-hydroxy carbonyl substrates bearing aryl and aliphatic substituents were synthesized and found to react well to afford the desired 4,5-disubstituted oxazolidin-2-ones in good to excellent conversions. Electron-neutral and electron-rich aryl motifs, such as *p*-chlorophenyl (**7a**), *p*-fluorophenyl (**7b**), *p*-methoxyphenyl (**7e**), and thiophene (**7c**) groups, readily proceeded to provide the desired products **8a**–**c** and **8e** (90–97%). Substrates with strong electron withdrawing functionalities, such as *p*-cyano (**7h**), p-nitro (**7i**), and *p*-trifluoromethyl (**7j**) were also tolerated to generate the corresponding oxazolidin-2-ones (**8h**–**8j**, 79–80%). In addition, expanding the scope to aliphatic substituents (**7k** and **7l**) proved to be feasible and provided the corresponding products **8k** and **8l** (73–90%), demonstrating excellent compatibility with a range of functional groups.

To demonstrate the synthetic utility of the present methodology, we tackled the synthesis (−)-cytoxazone (**1**). (−)-Cytoxazone was originally isolated from cultures of *Streptomyces* sp. in 1998 by Osada and co-workers [32]. Structural elucidation and relative stereochemistry was established by a combination of high-resolution mass spectroscopy and two-dimensional-NMR studies, while the absolute configuration was secured via the comparison of CD spectra with authentic samples, as well as the enantioselective synthesis by Nakata and co-workers [33]. It was reported that (−)-cytoxazone exhibited a cytokine modulator effect via the signaling pathway of Th2 cells (type 2 cytokines), which is involved in cell growth and differentiation [34]. These interesting pharmacological properties made this natural product and its analogs important targets for chemical synthesis, resulting in more than thirty to date [35,36,37,38,39].

**Scheme 5:**
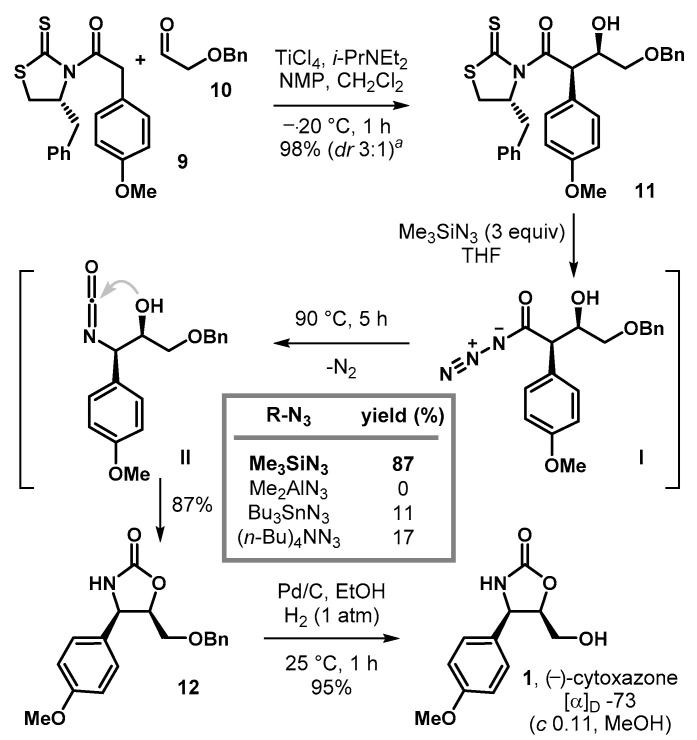
Concise total synthesis of (−)-cytoxazone (**1**). *^a^* A mixture of diastereomers (Evans *syn*: non-Evans *syn* = 3:1) was observed.

Our synthesis of (−)-cytoxazone (**1**) is illustrated in Scheme 5. An asymmetric aldol addition of chlorotitanium enolate of **9** upon treatment with 2-benzyloxyacetaldehyde **10** furnished the *syn*-aldol adduct **11** in 98% yield (*dr* 3:1). Subsequent nucleophilic azidation/Curtius reaction of the resulting **11** in the presence of trimethylsilyl azide (3 equiv., THF, 90 °C, 5 h) smoothly proceeded to afford the desired cyclic carbamate **12** in 87% yield as a single diastereomer. Attempts with other methods (Bu_3_SnN_3_ or (*n*-Bu)_4_NN_3_, 3 equiv., THF, 90 °C) were also successful but afforded **12** in lower conversions (11–17%). Lastly, removal of benzyl group (Pd/C, H_2_, EtOH) allowed the completion of the synthesis of (−)-cytoxazone (**1**), whose properties proved identical in all respects with those of an authentic sample of the natural product.

## 3. Experimental Section

### 3.1. General Information

All reactions were conducted in oven-dried glassware under nitrogen. Unless otherwise stated, all reagents were purchased from Sigma-Aldrich (St. Louis, MO, USA), Acros, or Fisher (Hampton, NH, USA), and were used without further purification. All solvents were ACS grade or better and used without further purification. Analytical thin layer chromatography (TLC) was performed with glass backed silica gel (60 Å) plates with fluorescent indication (Whatman, St. Louis, MO, USA). Visualization was accomplished by UV irradiation at 254 nm and/or by staining with ninhydrin, phosphomolybdic acid (PMA) solution, or *p*-anisaldehyde solution. Flash column chromatography was performed by using silica gel (particle size 70–230 mesh ASTM). All ^1^H-NMR and ^13^C-NMR spectra were recorded at 298 K on a Bruker Avance III HD 500 MHz (Bruker Corporation, Billerica, MA, USA) spectrometer in CDCl_3_ by using the signal of residual CHCl_3_, as an internal standard. All-NMR *δ* values are given in ppm, and all *J* values are in Hz. Optical rotation values were measured with a Rudolph Research Analytical (AUTOPOL II, Hackettstown, NJ, USA) polarimeter.

### 3.2. Representative Procedure for the Synthesis of ***4*** 

*(4S,5R)-4-methyl-5-phenyloxazolidin-2-one* (**4**): A solution of (2*S*,3*S*)-3-hydroxy-1-((*S*)-4-isopropyl-2-thioxothiazolidin-3-yl)-2-methyl-3-phenylpropan-1-one **3c** (38.9 mg, 0.120 mmol, 1.0 equiv.) in THF (1.2 mL, 0.1 M) was treated with Me_3_SiN_3_ (47.0 μL, 0.360 mmol, 3.0 equiv.) and the resulting mixture was heated to reflux at 90 °C. After stirring for 5 h at 90 °C, the resulting mixture was cooled to 25 °C, quenched with the addition of H_2_O, and diluted with CH_2_Cl_2_. The layers were separated, and the aqueous layer was extracted with CH_2_Cl_2_. The combined organic layers were washed with saturated aqueous NaCl, dried over anhydrous Na_2_SO_4_, and concentrated in vacuo. The residue was purified by column chromatography (SiO_2_, gradient eluent: 25–75% EtOAc/hexane) to provide (4*S*,5*R*)-4-methyl-5-phenyloxazolidin-2-one **4** (19.0 mg, 89%) as a white powder: [α]_D_^25^ –165 (*c* 0.60, CHCl_3_); ^1^H-NMR (500 MHz, CDCl_3_) *δ* 7.35–7.40 (m, 2H), 7.31–7.34 (m, 1H), 7.27–7.29 (m, 2H), 6.79 (br s, 1H), 5.70 (d, *J* = 8.1 Hz, 1H), 4.18–4.24 (m, 1H), 0.80 (d, *J* = 6.6 Hz, 3H); ^13^C-NMR (125 MHz, CDCl_3_) *δ* 159.8, 134.9, 128.4, 128.3, 125.8, 80.9, 52.3, 17.4; HRMS (Q–TOF) *m*/*z* 178.0874 [(M + H)^+^, C_10_H_12_NO_2_ requires 178.0868].

### 3.3. Synthesis of Oxazolidin-2-one (***8a**–**8l***)

A solution of **7a**–**7l** (1.0 equiv.) in THF (0.1 M) was treated with Me_3_SiN_3_ (3.0 equiv.) and the resulting mixture was heated to reflux at 90 °C. After stirring for 5 h at 90 °C, the resulting mixture was cooled to 25 °C, quenched with the addition of H_2_O, and diluted with CH_2_Cl_2_. The layers were separated, and the aqueous layer was extracted with CH_2_Cl_2_. The combined organic layers were washed with saturated aqueous NaCl, dried over anhydrous Na_2_SO_4_, and concentrated in vacuo. The residue was purified by column chromatography (SiO_2_, gradient eluent: 25–75% EtOAc/hexane) to provide **8a**–**8l**.

*(4S,5R)-5-(4-chlorophenyl)-4-methyloxazolidin-2-one* (**8a**): A white solid: [α]_D_^25^ –124 (*c* 0.72, CHCl_3_); ^1^H-NMR (500 MHz, CDCl_3_) *δ* 7.37 (d, *J* = 8.5 Hz, 2H), 7.23 (d, *J* = 8.4 Hz, 2H), 6.32 (br s, 1H), 5.68 (d, *J* = 8.0 Hz, 1H), 4.17–4.23 (m, 1H), 0.81 (d, *J* = 6.5 Hz, 3H); ^13^C-NMR (125 MHz, CDCl_3_) *δ* 159.3, 134.4, 133.4, 128.8, 127.3, 80.3, 52.2, 17.5; HRMS (Q–TOF) *m*/*z* 212.0484 [(M + H)^+^, C_10_H_11_ClNO_2_ requires 212.0478].

*(4S,5R)-5-(4-fluorophenyl)-4-methyloxazolidin-2-one* (**8b**): A white solid: [α]_D_^25^ –104 (c 0.27, CHCl_3_); ^1^H-NMR (500 MHz, CDCl_3_) *δ* 7.26–7.30 (m, 2H), 7.09 (td, *J* = 8.7, 2.5 Hz, 2H), 5.87 (br s, 1H), 5.70 (d, *J* = 7.9 Hz, 1H), 4.17–4.23 (m, 1H), 0.81 (d, *J* = 6.5 Hz, 3H); ^13^C-NMR (125 MHz, CDCl_3_) *δ* 162.7 (d, *J* = 245.8 Hz), 159.1, 130.6 (d, *J* = 3.2 Hz), 127.7 (d, *J* = 8.2 Hz), 115.6 (d, *J* = 21.6 Hz), 80.4, 52.3, 17.5; HRMS (Q–TOF) m/z 196.0779 [(M + H)^+^, C_10_H_11_FNO_2_ requires 196.0774].

*(4S,5R)-4-methyl-5-(thiophen-2-yl)oxazolidin-2-one* (**8c**): A white oil: [α]_D_^25^ –33.3 (*c* 0.09, CHCl_3_); ^1^H-NMR (500 MHz, CDCl_3_) *δ* 7.34 (dd, *J* = 4.9, 1.3 Hz, 1H), 7.02–7.07 (m, 2H), 5.92 (d, *J* = 7.8 Hz, 1H), 5.57 (br s, 1H), 4.17–4.24 (m, 1H), 1.00 (d, *J* = 6.5 Hz, 3H); ^13^C-NMR (125 MHz, CDCl_3_) *δ* 158.5, 137.1, 126.9, 126.1, 125.9, 78.2, 52.6, 17.0; HRMS (Q–TOF) *m*/*z* 184.0439 [(M + H)^+^, C_8_H_10_NO_2_S requires 184.0432].

*(4S,5R)-5-(4-(benzyloxy)-3-methoxyphenyl)-4-methyloxazolidin-2-one* (**8d**): A white oil: [α]_D_^25^ –50.0 (*c* 0.20, CHCl_3_); ^1^H-NMR (500 MHz, CDCl_3_) *δ* 7.43 (d, *J* = 7.2 Hz, 2H), 7.35–7.39 (m, 2H), 7.29–7.33 (m, 1H), 6.89 (d, *J* = 8.3 Hz, 1H), 6.85 (d, *J* = 1.9 Hz, 1H), 6.74 (dd, *J* = 8.3, 1.9 Hz, 1H), 5.65 (d, *J* = 7.9 Hz, 1H), 5.44 (br s, 1H), 5.16 (s, 2H), 4.11–4.17 (m, 1H), 3.90 (s, 3H), 0.83 (d, *J* = 6.5 Hz, 3H); ^13^C-NMR (125 MHz, CDCl_3_) *δ* 159.0, 149.7, 148.2, 136.8, 128.6, 127.9, 127.7, 127.3, 118.4, 113.7, 109.4, 80.9, 71.0, 56.1, 52.5, 17.5; HRMS (Q–TOF) *m*/*z* 314.1393 [(M + H)^+^, C_18_H_20_NO_4_ requires 314.1392].

*(4S,5R)-5-(4-methoxyphenyl)-4-methyloxazolidin-2-one* (**8e**): A white solid: [α]_D_^25^ –87.4 (*c* 0.24, CHCl_3_); ^1^H-NMR (500 MHz, CDCl_3_) *δ* 7.21 (d, *J* = 8.7 Hz, 2H), 6.91 (d, *J* = 8.7 Hz, 2H), 5.79 (br s, 1H), 5.67 (d, *J* = 7.9 Hz, 1H), 4.13–4.20 (m, 1H), 3.82 (s, 3H), 0.82 (d, *J* = 6.5 Hz, 3H); ^13^C-NMR (125 MHz, CDCl_3_) *δ* 159.7, 159.4, 127.3, 126.8, 113.9, 80.9, 55.3, 52.5, 17.5; HRMS (Q–TOF) *m*/*z* 208.0980 [(M + H)^+^, C_11_H_14_NO_3_ requires 208.0974].

*(4S,5R)-5-(7-methoxybenzo[d][1,3]dioxol-5-yl)-4-methyloxazolidin-2-one* (**8f**): A colorless oil: [α]_D_^25^ –122 (*c* 0.24, CHCl_3_); ^1^H-NMR (500 MHz, CDCl_3_) *δ* 6.48 (d, *J* = 1.1 Hz, 1H), 6.45 (d, *J* = 1.1 Hz, 1H), 5.99 (s, 2H), 5.82 (br s, 1H), 6.11 (d, *J* = 7.9 Hz, 1H), 4.11–4.17 (m, 1H), 3.90 (s, 3H), 0.86 (d, *J* = 6.5 Hz, 3H); ^13^C-NMR (125 MHz, CDCl_3_) *δ* 159.1, 149.1, 143.7, 135.2, 129.2, 105.5, 101.7, 100.2, 80.8, 56.7, 52.5, 17.4; HRMS (Q–TOF) *m*/*z* 252.0876 [(M + H)^+^, C_12_H_14_NO_5_ requires 252.0872].

*(4S,5R)-4-methyl-5-(3,4,5-trimethoxyphenyl)oxazolidin-2-one* (**8g**): A colorless oil: [α]_D_^25^ –53.8 (*c* 0.39, CHCl_3_); ^1^H-NMR (500 MHz, CDCl_3_) *δ* 6.49 (s, 2H), 5.86 (br s, 1H), 5.65 (d, *J* = 7.9 Hz, 1H), 4.13–4.19 (m, 1H), 3.86 (s, 6H), 3.85 (s, 3H), 0.86 (d, *J* = 6.5 Hz, 3H); ^13^C-NMR (125 MHz, CDCl_3_) *δ* 159.2, 153.4, 137.8, 130.3, 102.8, 80.9, 60.9, 56.2, 52.5, 17.4; HRMS (Q–TOF) *m*/*z* 268.1186 [(M + H)^+^, C_13_H_18_NO_5_ requires 268.1185].

*4-((4S,5R)-4-methyl-2-oxooxazolidin-5-yl)benzonitrile* (**8h**): A white solid: [α]_D_^25^ –160 (*c* 0.05, CHCl_3_); ^1^H-NMR (500 MHz, CDCl_3_) *δ* 7.72 (d, *J* = 8.4 Hz, 2H), 7.44 (d, *J* = 8.2 Hz, 2H), 5.75 (d, *J* = 7.9 Hz, 1H), 5.61 (br s, 1H), 4.23–4.29 (m, 1H), 0.81 (d, *J* = 6.5 Hz, 3H); ^13^C-NMR (125 MHz, CDCl_3_) *δ* 158.3, 140.1, 132.4, 126.7, 118.2, 112.6, 79.9, 52.0, 17.6; HRMS (Q–TOF) *m*/*z* 203.0829 [(M + H)^+^, C_11_H_11_N_2_O_2_ requires 203.0821].

*(4S,5R)-4-methyl-5-(4-nitrophenyl)oxazolidin-2-one* (**8i**): A white solid: [α]_D_^25^ –225 (*c* 0.04, CHCl_3_); ^1^H-NMR (500 MHz, CDCl_3_) *δ* 8.27 (dd, *J* = 7.0, 1.8 Hz, 2H), 7.51 (d, *J* = 8.6 Hz, 2H), 5.81 (d, *J* = 8.0 Hz, 1H), 5.68 (br s, 1H), 4.26–4.33 (m, 1H), 0.83 (d, *J* = 6.5 Hz, 3H); ^13^C-NMR (125 MHz, CDCl_3_) *δ* 158.3, 148.1, 142.0, 126.9, 123.9, 79.7, 52.0, 17.7; HRMS (Q–TOF) *m*/*z* 223.0723 [(M + H)^+^, C_10_H_11_N_2_O_4_ requires 223.0719].

*(4S,5R)-4-methyl-5-(4-(trifluoromethyl)phenyl)oxazolidin-2-one* (**8j**): A white solid: [α]_D_^25^ –72.3 (c 0.65, CHCl_3_); ^1^H-NMR (500 MHz, CDCl_3_) *δ* 7.67 (d, *J* = 8.2 Hz, 2H), 7.44 (d, *J* = 8.2 Hz, 2H), 5.91 (br s, 1H), 5.77 (d, *J* = 7.7 Hz, 1H), 4.23–4.29 (m, 1H), 0.82 (d, *J* = 6.5 Hz, 3H); ^13^C-NMR (125 MHz, CDCl_3_) *δ* 158.9, 138.9, 130.8 (q, *J* = 32.5 Hz), 126.3, 125.6 (q, *J* = 3.8 Hz), 123.8 (q, *J* = 270.6 Hz), 80.1, 52.1, 17.6; HRMS (Q–TOF) m/z 246.0744 [(M + H)^+^, C_11_H_11_F_3_NO_2_ requires 246.0742].

*(4S,5R)-5-heptyl-4-methyloxazolidin-2-one* (**8k**): A colorless oil: [α]_D_^25^ +12.6 (*c* 0.34, CHCl_3_); ^1^H-NMR (500 MHz, CDCl_3_) *δ* 6.06 (br s, 1H), 4.52–4.57 (m, 1H), 3.85–3.92 (m, 1H), 1.68–1.76 (m, 1H), 1.46–1.52 (m, 2H), 1.26–1.33 (m, 9H), 1.14 (d, *J* = 6.5 Hz, 3H), 0.87 (t, *J* = 6.9 Hz, 3H); ^13^C-NMR (125 MHz, CDCl_3_) *δ* 159.8, 80.2, 51.1, 31.7, 29.3, 29.10, 29.05, 25.8, 22.6, 15.9, 14.0; HRMS (Q–TOF) *m*/*z* 208.1314 [(M+Na)^+^, C_10_H_19_NNaO_2_ requires 208.1313].

*(4S,5R)-5-butyl-4-methyloxazolidin-2-one* (**8l**): A colorless oil: [α]_D_^25^ +14.8 (*c* 0.27, CHCl_3_); ^1^H-NMR (500 MHz, CDCl_3_) *δ* 5.70 (br s, 1H), 4.53–4.58 (m, 1H), 3.86–3.92 (m, 1H), 1.70–1.77 (m, 1H), 1.48–1.56 (m, 2H), 1.31–1.41 (m, 3H), 1.16 (d, *J* = 6.5 Hz, 3H), 0.92 (t, *J* = 7.1 Hz, 3H); ^13^C-NMR (125 MHz, CDCl_3_) *δ* 159.6, 80.2, 51.1, 28.8, 27.9, 22.4, 15.9, 13.9; HRMS (Q–TOF) *m*/*z* 144.1032 [(M + H)^+^, C_7_H_14_NO_2_ requires 144.1025].

### 3.4. Synthesis of (–)-Cytoxazone

*(2R,3R)-1-((R)-4-benzyl-2-thioxothiazolidin-3-yl)-4-(benzyloxy)-3-hydroxy-2-(4-methoxyphenyl)butan-1-one* (**11**): A cooled (0 °C) solution of (*R*)-1-(4-benzyl-2-thioxothiazolidin-3-yl)-2-(4-methoxyphenyl)ethan-1-one **9** (118 mg, 0.330 mmol, 1.0 equiv.) in CH_2_Cl_2_ (3.3 mL, 0.1 M) was treated with titanium(IV) chloride (0.36 mL, 1.0 M in CH_2_Cl_2_, 0.36 mmol, 1.1 equiv.). After stirring for 30 min at 0 °C, *i*-Pr_2_NEt (0.14 mL, 0.83 mmol, 2.5 equiv.) was added dropwise and the resulting mixture was stirred for 2 h at 0 °C. 1-methyl-2-pyrrolidinone (NMP, 64 μL, 0.66 mmol, 2.0 equiv.) was added, and the resulting mixture was stirred for an additional 1 h and then cooled to −20 °C. 2-(benzyloxy)acetaldehyde **10** (148 mg, 0.990 mmol, 3 equiv.) in CH_2_Cl_2_ (2 mL) was added to the above enolate, and the resulting mixture was stirred for 1 h before it was quenched with the addition of saturated aqueous NH_4_Cl, and diluted with CH_2_Cl_2_. The layers were separated, and the aqueous layer was extracted with CH_2_Cl_2_. The combined organic layers were washed with saturated aqueous NaCl, dried over anhydrous Na_2_SO_4_, and concentrated in vacuo. The residue was purified by column chromatography (SiO_2_, 20% EtOAc/hexane) to provide an Evans *syn* aldol adduct **11** (124 mg, 74%) and a non-Evans *syn* aldol adduct (40 mg, 24%) as yellow oils: For **11**: [α]_D_^25^ +8.3 (*c* 0.44, CHCl_3_); ^1^H-NMR (500 MHz, CDCl_3_) *δ* 7.38–7.42 (m, 2H), 7.28–7.36 (m, 6H), 7.23–7.27 (m, 2H), 7.18–7.21 (m, 2H), 6.86–6.90 (m, 2H), 5.86 (d, *J* = 6.7 Hz, 1H), 5.25 (ddd, *J* = 10.5, 6.6, 3.8 Hz, 1H), 4.53 (s, 2H), 4.43 (dd, *J* = 12.2, 5.6 Hz, 1H), 3.80 (s, 3H), 3.57 (dd, *J* = 9.8, 5.3 Hz, 1H), 3.48 (dd, *J* = 9.8, 5.7 Hz, 1H), 3.08 (dd, *J* = 11.5, 7.3 Hz, 1H), 3.04 (dd, *J* = 13.5, 3.8 Hz, 1H), 2.83 (dd, *J* = 13.5, 10.7 Hz, 1H), 2.73 (dd, *J* = 11.5, 0.6 Hz, 1H); ^13^C-NMR (125 MHz, CDCl_3_) *δ* 200.7, 173.6, 159.1, 137.8, 136.4, 131.4, 129.3, 128.9, 128.4, 127.78, 127.76, 127.2, 125.5, 113.9, 73.5, 72.5, 71.9, 68.7, 55.2, 51.7, 36.6, 31.7; HRMS (Q–TOF) *m*/*z* 506.1458 [(M – H)^+^, C_28_H_28_NO_4_S_2_ requires 506.1460].

*(4R,5R)-5-((benzyloxy)methyl)-4-phenyloxazolidin-2-one* (**12**): A solution of **11** (28.5 mg, 0.056 mmol, 1.0 equiv.) in THF (1.4 mL, 0.04 M) was treated with Me_3_SiN_3_ (22.0 μL, 0.168 mmol, 3.0 equiv.) and the resulting mixture was heated to reflux at 90 °C. After stirring for 5 h at 90 °C, the resulting mixture was cooled to 25 °C, quenched with the addition of H_2_O and diluted with CH_2_Cl_2_. The layers were separated, and the aqueous layer was extracted with CH_2_Cl_2_. The combined organic layers were washed with saturated aqueous NaCl, dried over anhydrous Na_2_SO_4_, and concentrated in vacuo. The residue was purified by column chromatography (SiO_2_, gradient eluent: 25–50% EtOAc/hexane) to provide **12** (15.2 mg, 87%) as white oil: [α]_D_^25^ −13.3 (*c* 0.15, MeOH); ^1^H-NMR (500 MHz, CDCl_3_) *δ* 7.24–7.32 (m, 4H), 7.16–7.21 (m, 4H), 6.87–6.91 (m, 2H), 5.36 (br s, 1H), 4.98 (dt, *J* = 8.1, 5.8 Hz, 1H), 4.94 (d, *J* = 8.2 Hz, 1H), 4.25 (ABX, 2H, *J* = 11.7 Hz, Δν = 60.0 Hz), 3.83 (s, 3H), 3.37 (dd, *J* = 10.3, 6.1 Hz, 1H), 3.13 (dd, *J* = 10.3, 5.6 Hz, 1H); ^13^C-NMR (125 MHz, CDCl_3_) *δ* 160.0, 158.8, 137.4, 128.4, 128.3, 127.9, 127.8, 127.6, 114.1, 78.9, 73.4, 68.6, 57.8, 55.3; HRMS (Q–TOF) *m*/*z* 312.1225 [(M − H)^+^, C_18_H_18_NO_4_ requires 312.1236].

(*−*)*-Cytoxazone* (**1**): A solution of **12** (13 mg, 0.041 mmol, 1 equiv.) in EtOH (1.0 mL, 0.04 M) was treated with Pd/C (10%, 65 mg) and hydrogenated at 1 atm. After stirring for 1 h at 25 °C, the resulting mixture was filtered through a pad of Celite and concentrated in vacuo. The residue was purified by column chromatography (SiO_2_, 75% EtOAc/hexane) to provide (−)-cytoxazone (**1**, 8.7 mg, 95%) as a white oil whose spectral data were identical to those of the known synthetic **1** [40,41]: [α]_D_^25^ −72.6 (*c* 0.11, MeOH) vs [α]_D_^25^ −70.5 (*c* 0.8, MeOH) [40] and [α]_D_^25^ −70.9 (*c* 0.4, MeOH) [41]; ^1^H-NMR (500 MHz, DMSO-*d_6_*); *δ* 8.07 (br s, 1H), 7.14–7.16 (m, 2H), 6.92–6.94 (m, 2H), 5.90 (d, *J* = 8.3 Hz, 1H), 4.85 (t, *J* = 5.2 Hz, 1H), 4.70 (td, *J* = 8.0, 4.1 Hz, 1H), 3.74 (s, 3H), 2.91–3.00 (m, 2H); ^13^C-NMR (125 MHz, DMSO-d_6_) *δ* 159.1, 158.9, 129.3, 128.1, 113.7, 80.1, 61.1, 56.2, 55.2; HRMS (Q–TOF) *m*/*z* 246.0740 [(M + Na)^+^, C_11_H_13_NNaO_4_ requires 246.0742].

## 4. Conclusions

In conclusion, we investigated the scope and utility of a combination of the asymmetric aldol/Curtius protocol in the context of the synthesis of oxazolidin-2-one scaffolds bearing 4,5-two vicinal functionalities and their necessary stereogenic centers, due to its interesting structural motifs in natural products and diversity of pharmacological properties. The developed strategy also allows for a straightforward and concise asymmetric total synthesis of (−)-cytoxazone in only three steps, making it one of the shortest syntheses reported to date. Continued examination of the synthetic applications of the developed strategy in the synthesis of bioactive natural products and pharmaceuticals is in progress and will be disclosed in due course.

## Data Availability

The data presented in this study are available in Supplementary Material.

## Supplementary Materials

The following are available online. Copies of ^1^H and ^13^C-NMR spectra of **1**,**4**,**8a**–**8l**,**11**,**12**.

## Sample Availability

Samples of the compounds **4**,**8a**–**8l** are available from the authors.
