# Supplementary material for: Stereoselective Synthesis of Oxazolidin-2-ones via an Asymmetric Aldol/Curtius Reaction: Concise Total Synthesis of (−)-Cytoxazone
Source: Molecules. 2021 Jan 23;26(3):597. doi: 10.3390/molecules26030597 (PMC7865922; doi:10.3390/molecules26030597)

Supplementary Material

Stereoselective Synthesis of Oxazolidin-2-ones via an Asymmetric Aldol/Curtius Reaction: Concise Total Synthesis of (–)-Cytoxazone

Hosam Choi, Hanho Jang, Joohee Choi and Kiyoun Lee *

*Correspondence: kiyoun@catholic.ac.kr; Tel.: +82-2-2164-5528; Fax: +82-2-2164-4764

Figure S1: Chromatographs of racemic (±)-4 and synthetic (+)-4

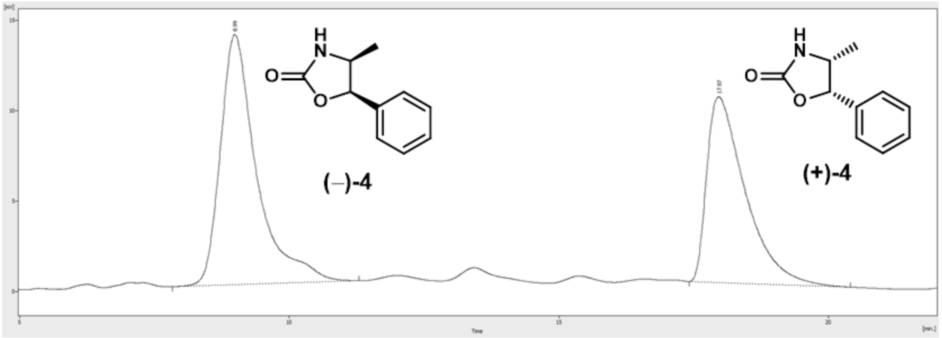

	Reten. Time [min]	Area [mV.s]	Height [mV]	Area [%]	Height [%]
1	8.993	648.795	13.837	56.0	57.4
2	17.970	510.079	10.281	44.0	42.6
	Total	1158.874	24.118	100.0	100.0

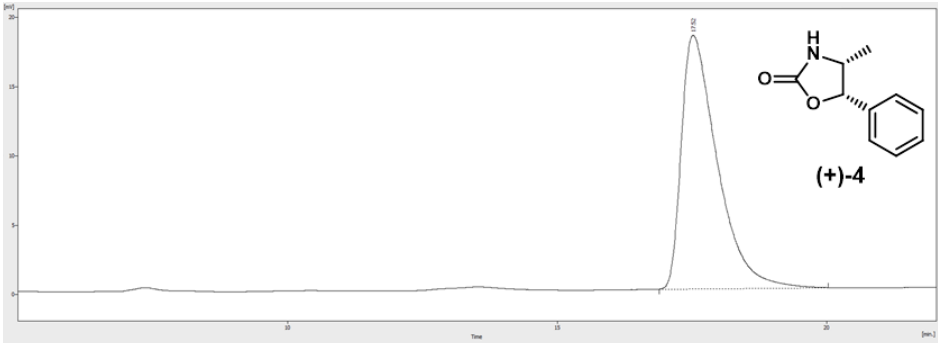

	Reten. Time [min]	Area [mV.s]	Height [mV]	Area [%]	Height [%]
1	17.523	847.859	18.309	100.0	100.0
	Total	847.859	18.309	100.0	100.0

Chiral HPLC (Analytical ChiralCel OD-H (4.6 x 250 mm), flow rate: 1.0 mL/min, isocratic 10% *i*-PrOH–Hexane). For enantiomer and *ent*-(–)-**4** (t_R: 9.00 min) and (+)-**4** (t_R: 17.5 min, $[\alpha]_D^{25}$ +165 (*c* 0.60, $CHCl_3$))

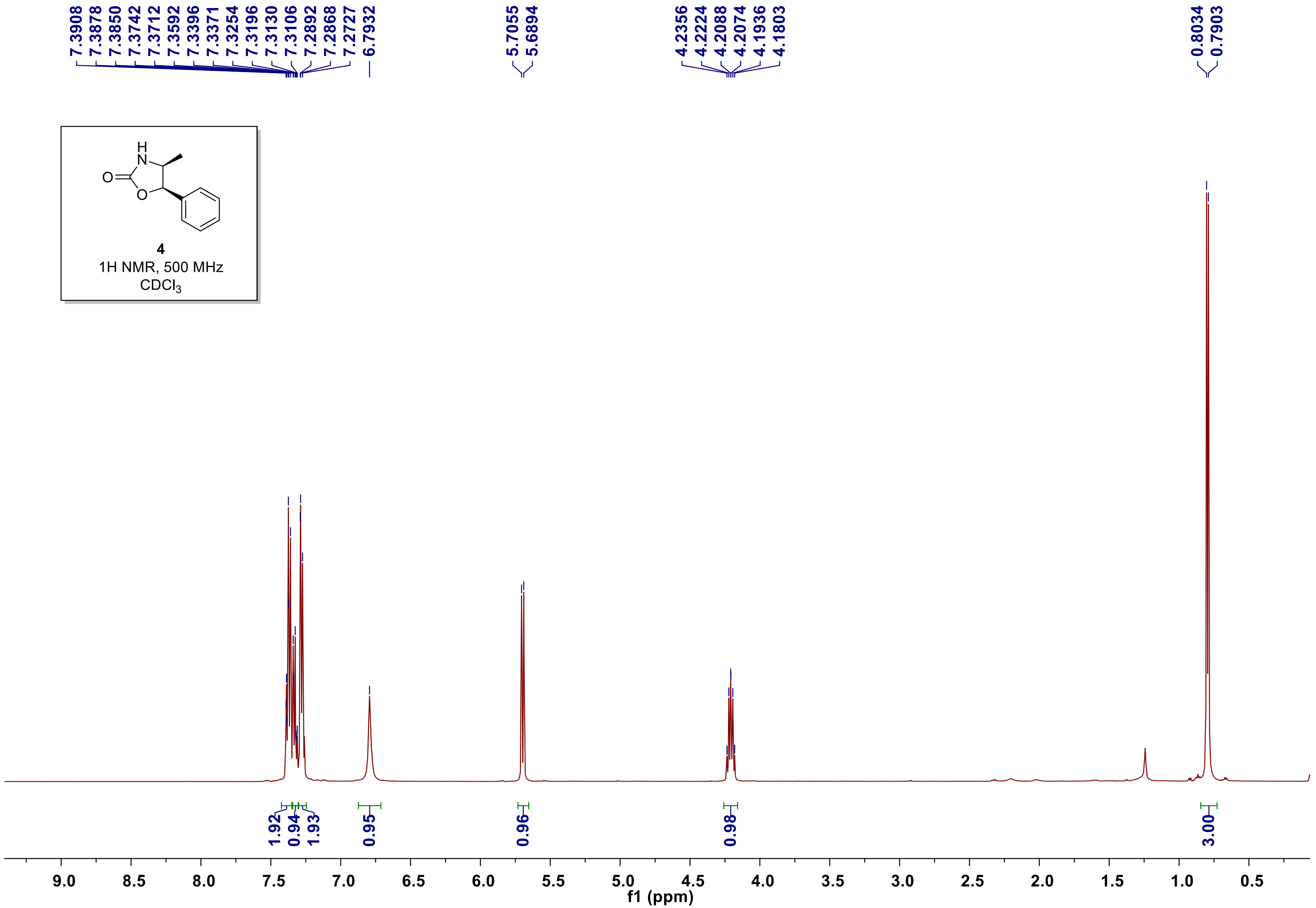

7.3908
7.3878
7.3850
7.3742
7.3712
7.3592
7.3396
7.3371
7.3254
7.3196
7.3130
7.3106
7.2892
7.2868
7.2727
6.7932
5.7055
5.6894
4.2356
4.2224
4.2088
4.2074
4.1936
4.1803
0.8034
0.7903
H
N
O
O
4
1H NMR, 500 MHz
CDCl3
1.92
0.94
1.93
0.95
0.96
0.98
3.00
9.0
8.5
8.0
7.5
7.0
6.5
6.0
5.5
5.0
4.5
4.0
3.5
3.0
2.5
2.0
1.5
1.0
0.5
f1 (ppm)

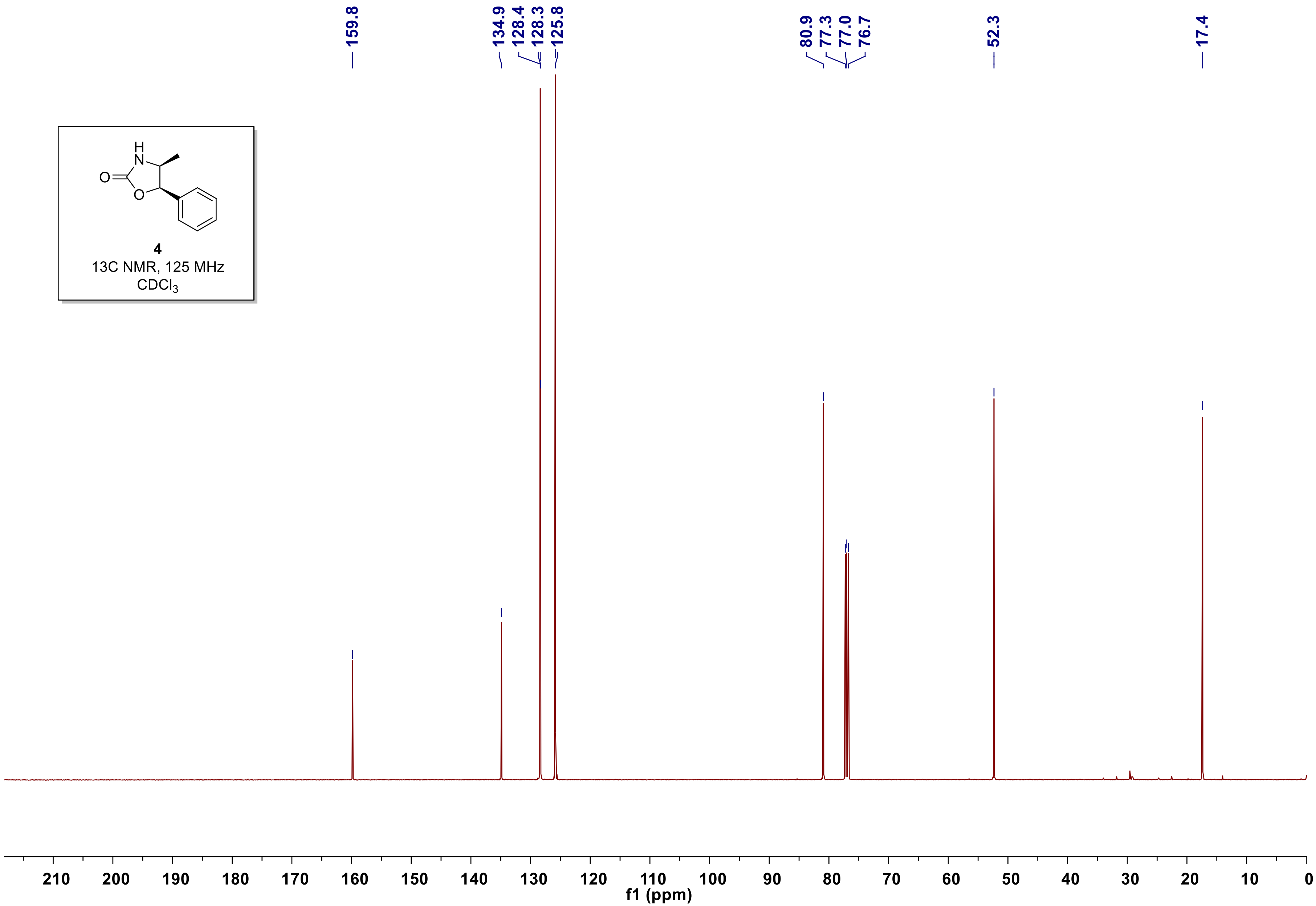
159.8
134.9
128.4
128.3
125.8
80.9
77.3
77.0
76.7
52.3
17.4
4
13C NMR, 125 MHz
CDCl3
210 200 190 180 170 160 150 140 130 120 110 100 90 80 70 60 50 40 30 20 10 0
f1 (ppm)

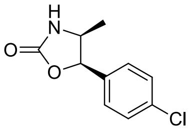

8a
1H NMR, 500 MHz
$CDCl_3$

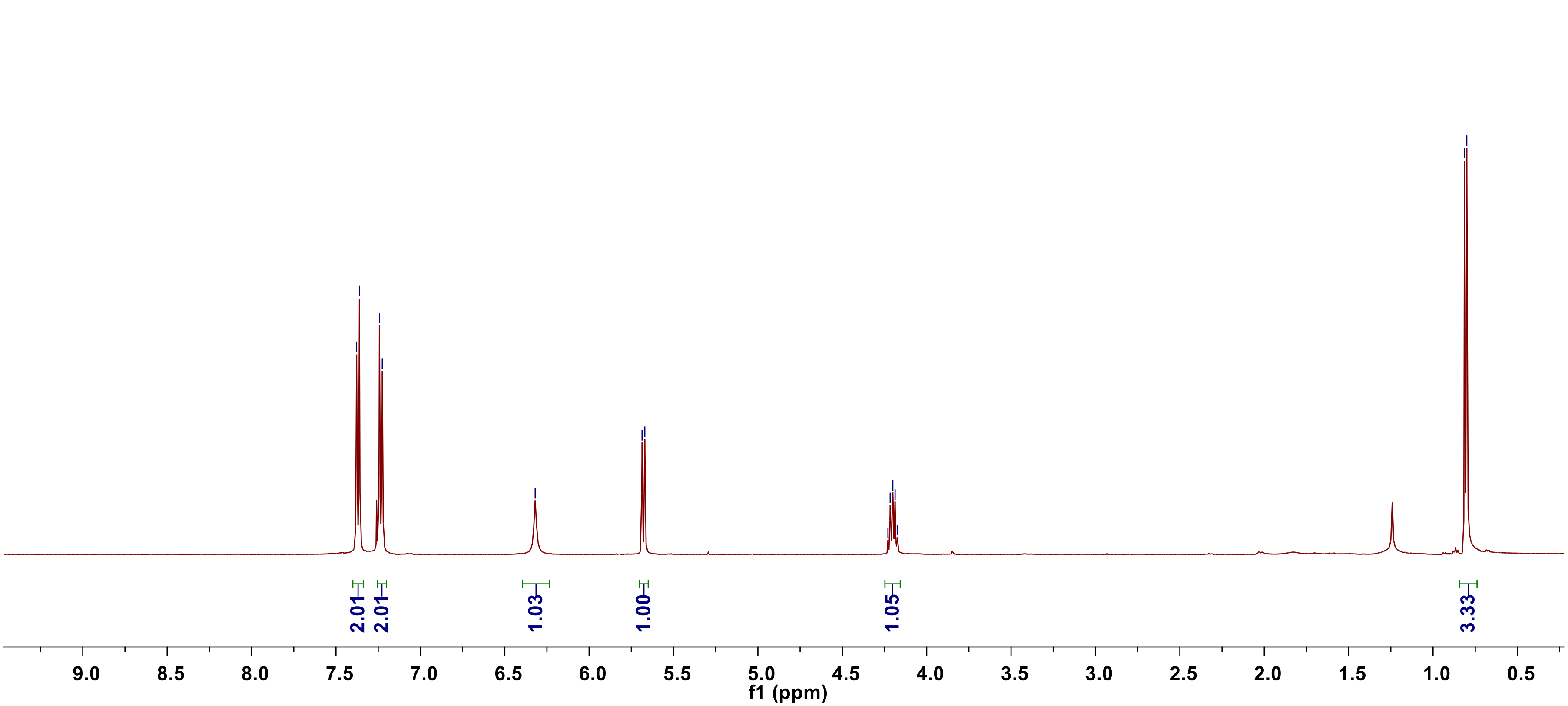

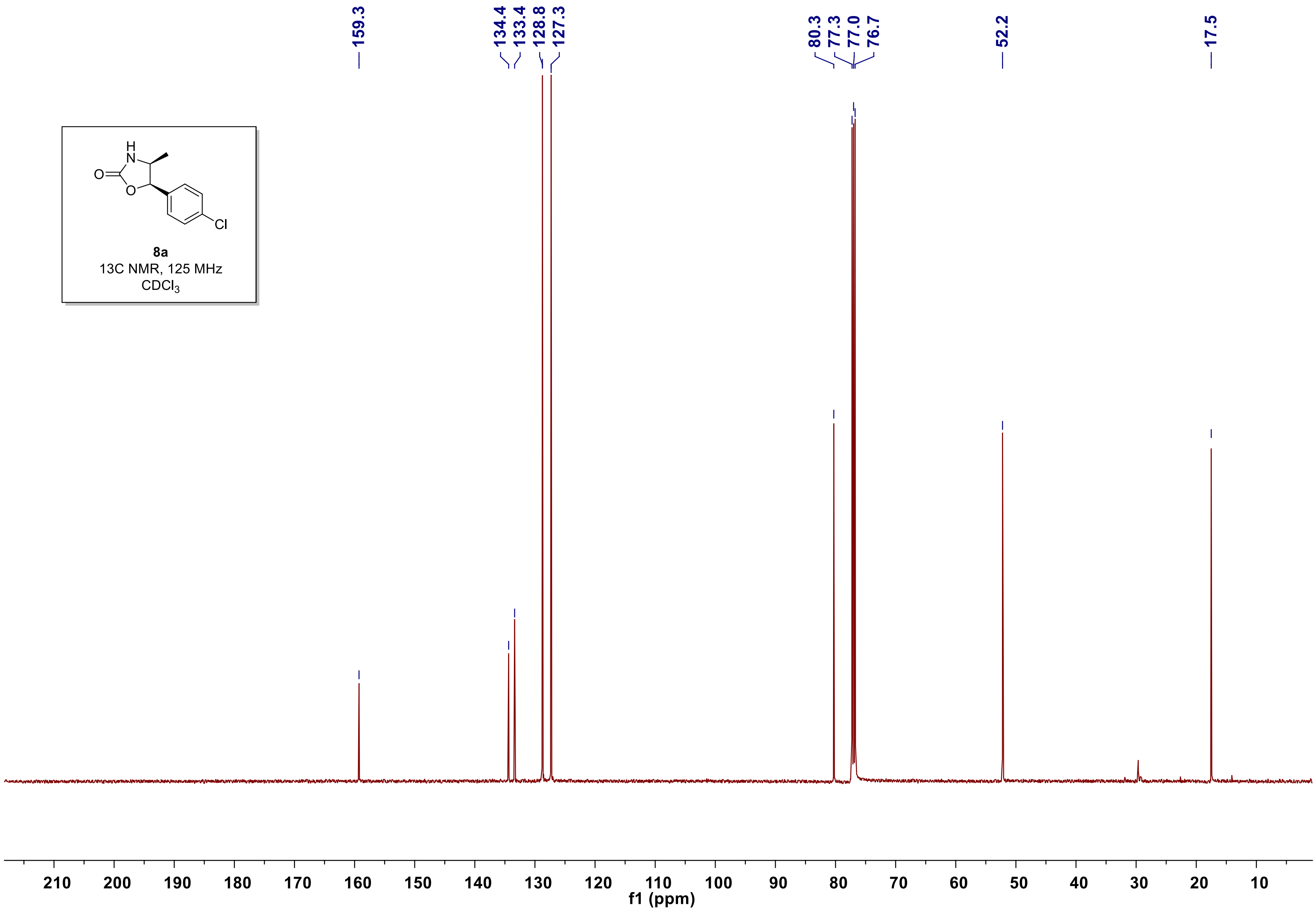
159.3
134.4
133.4
128.8
127.3
80.3
77.3
77.0
76.7
52.2
17.5
8a
13C NMR, 125 MHz
CDCl3
f1 (ppm)

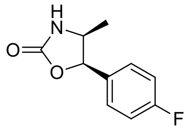

8b
1H NMR, 500 MHz
$CDCl_3$

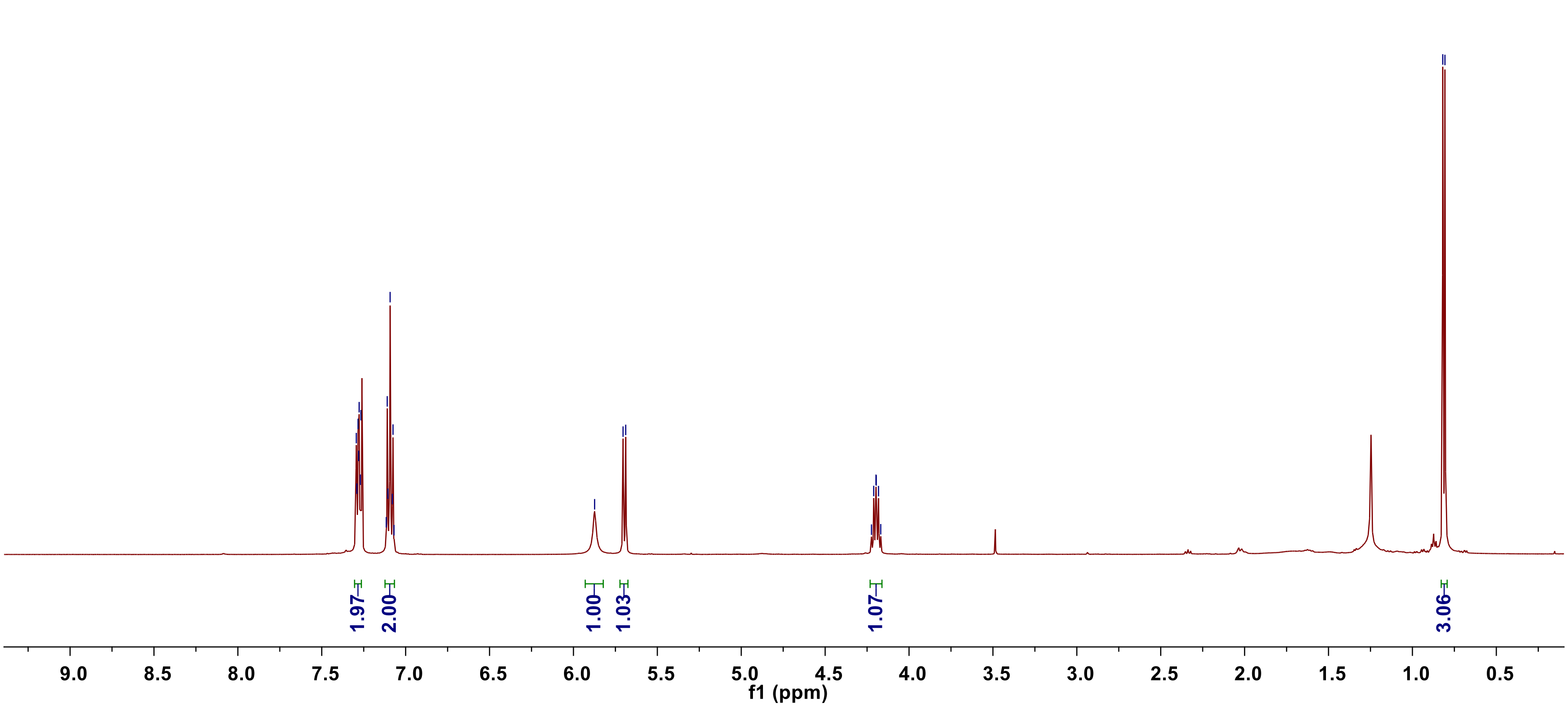

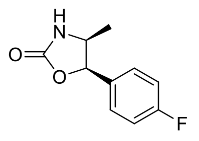
8b
13C NMR, 125 MHz
$CDCl_3$

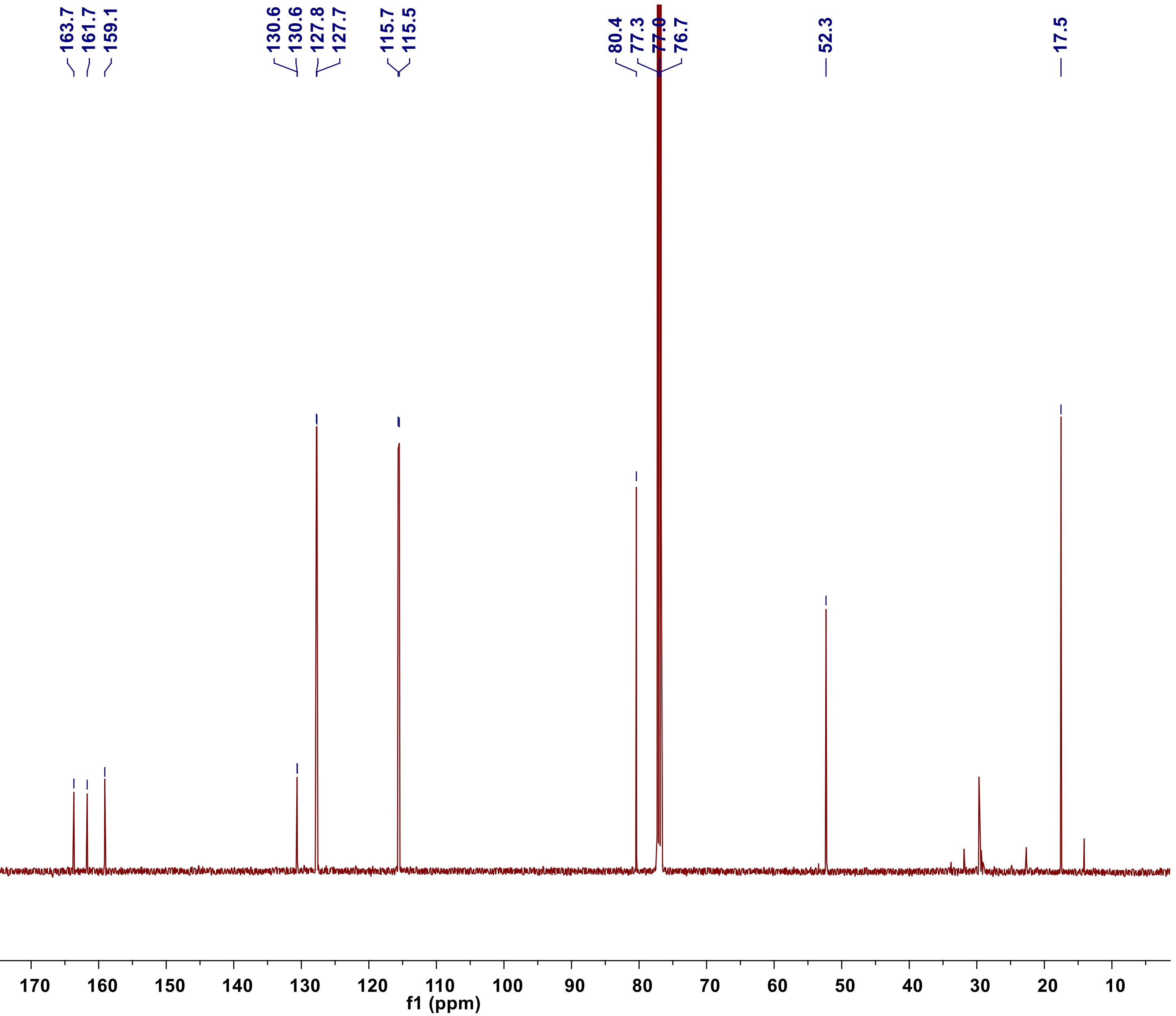
163.7
161.7
159.1
130.6
130.6
127.8
127.7
115.7
115.5
80.4
77.3
77.0
76.7
52.3
17.5
210
200
190
180
170
160
150
140
130
120
110
100
90
80
70
60
50
40
30
20
10
f1 (ppm)

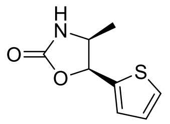

8c
1H NMR, 500 MHz
$CDCl_3$

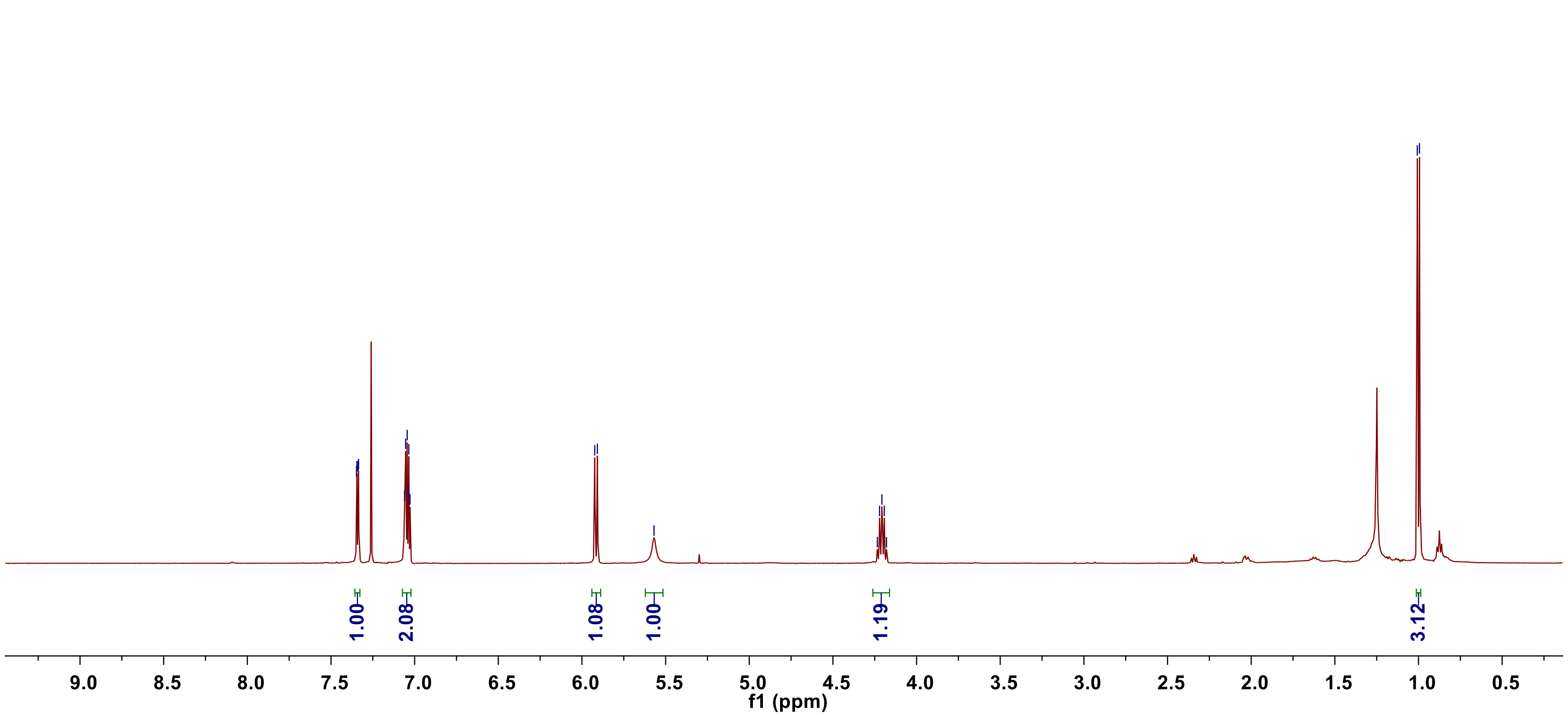

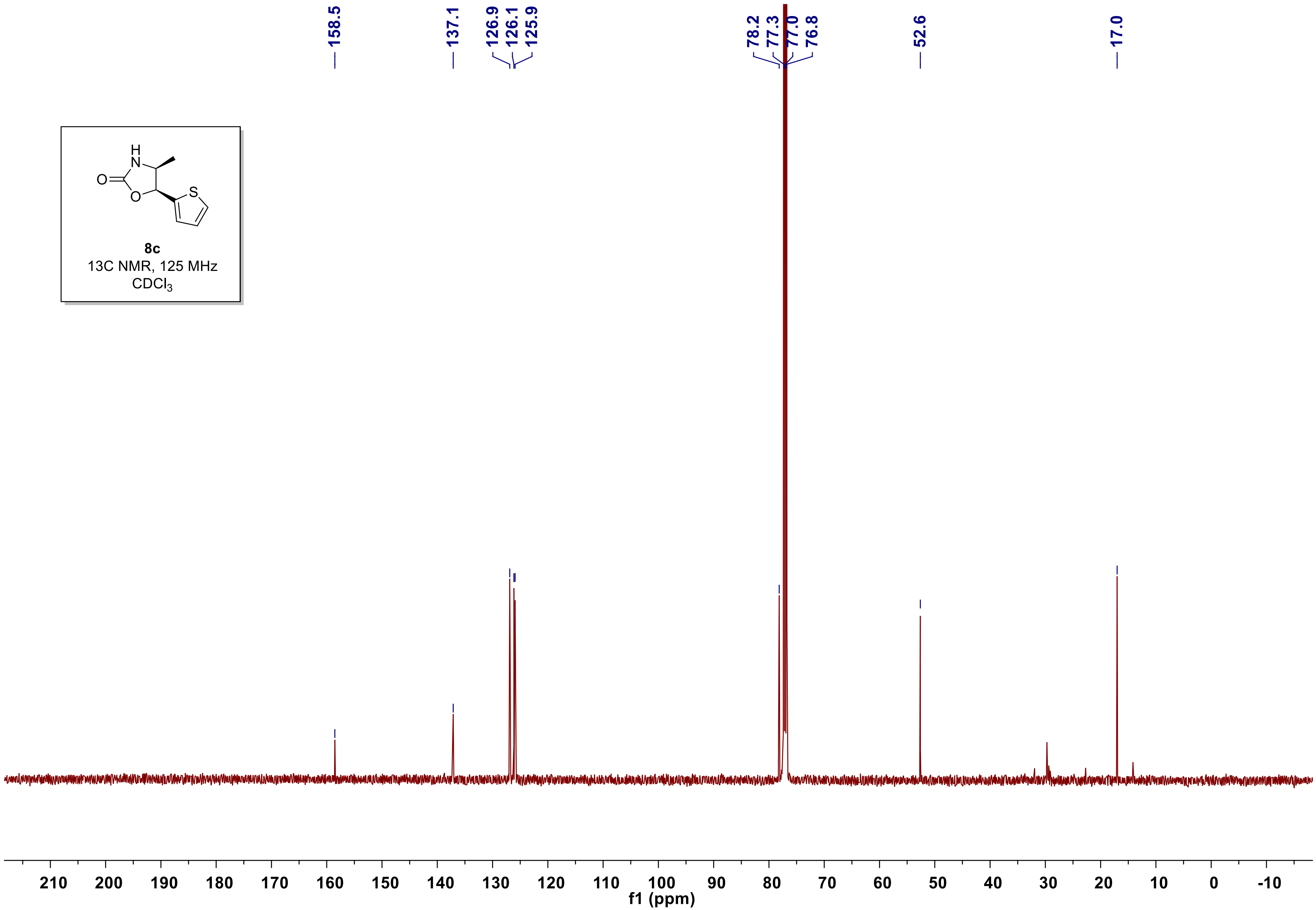

8c
13C NMR, 125 MHz
$CDCl_3$

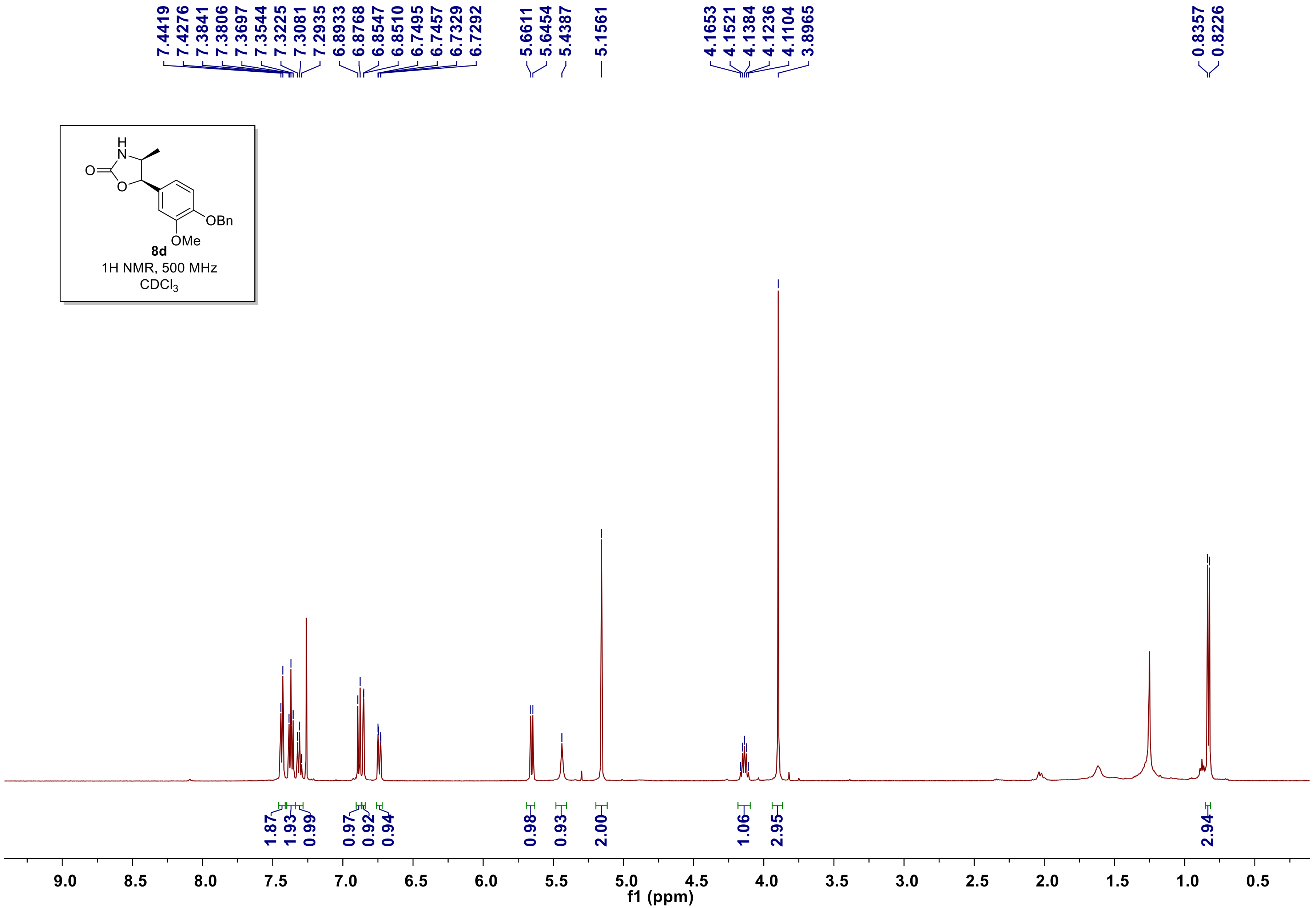

7.4419
7.4276
7.3841
7.3806
7.3697
7.3544
7.3225
7.3081
7.2935
6.8933
6.8768
6.8547
6.8510
6.7495
6.7457
6.7329
6.7292
5.6611
5.6454
5.4387
5.1561
4.1653
4.1521
4.1384
4.1236
4.1104
3.8965
0.8357
0.8226
8d
1H NMR, 500 MHz
CDCl3
1.87
1.93
0.99
0.97
0.92
0.94
0.98
0.93
2.00
1.06
2.95
2.94
f1 (ppm)

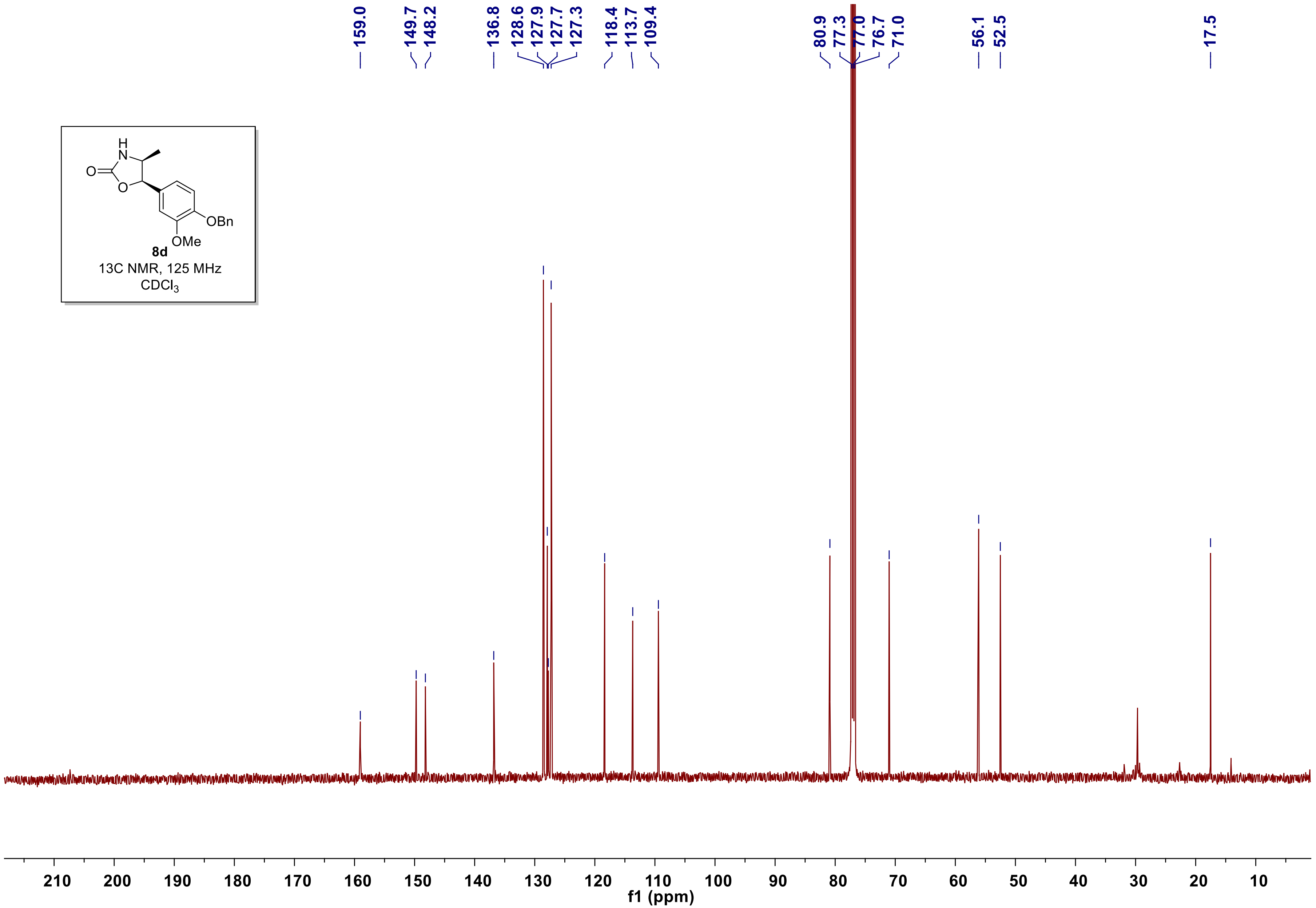
159.0
149.7
148.2
136.8
128.6
127.9
127.7
127.3
118.4
113.7
109.4
80.9
77.3
77.0
76.7
71.0
56.1
52.5
17.5
O
H
N
O
OBn
OMe
8d
13C NMR, 125 MHz
CDCl3
210 200 190 180 170 160 150 140 130 120 110 100 90 80 70 60 50 40 30 20 10
f1 (ppm)

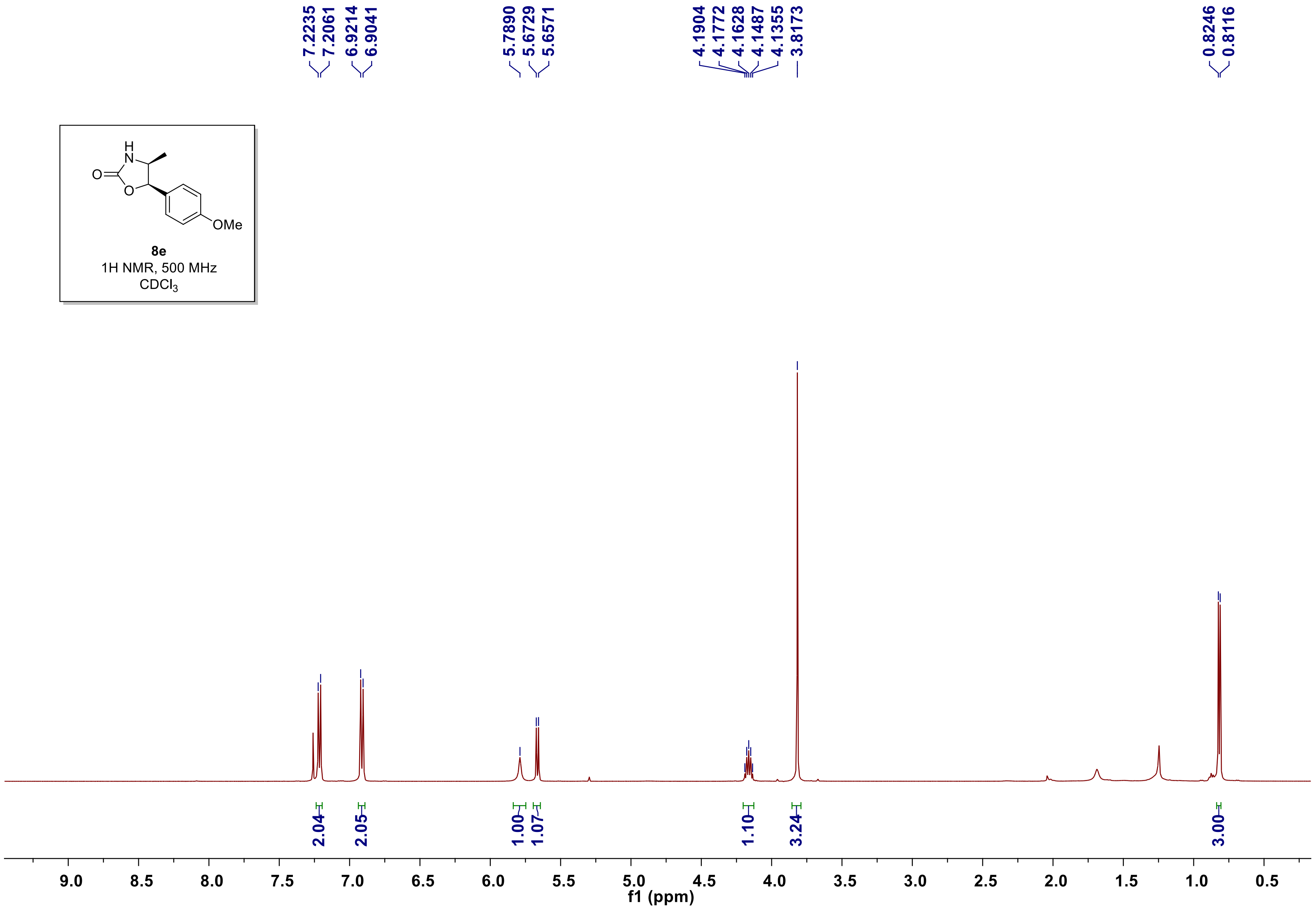
7.2235
7.2061
6.9214
6.9041
5.7890
5.6729
5.6571
4.1904
4.1772
4.1628
4.1487
4.1355
3.8173
0.8246
0.8116
H
N
O
O
OMe
8e
1H NMR, 500 MHz
CDCl3
2.04
2.05
1.00
1.07
1.10
3.24
3.00
9.0
8.5
8.0
7.5
7.0
6.5
6.0
5.5
5.0
4.5
4.0
3.5
3.0
2.5
2.0
1.5
1.0
0.5
f1 (ppm)

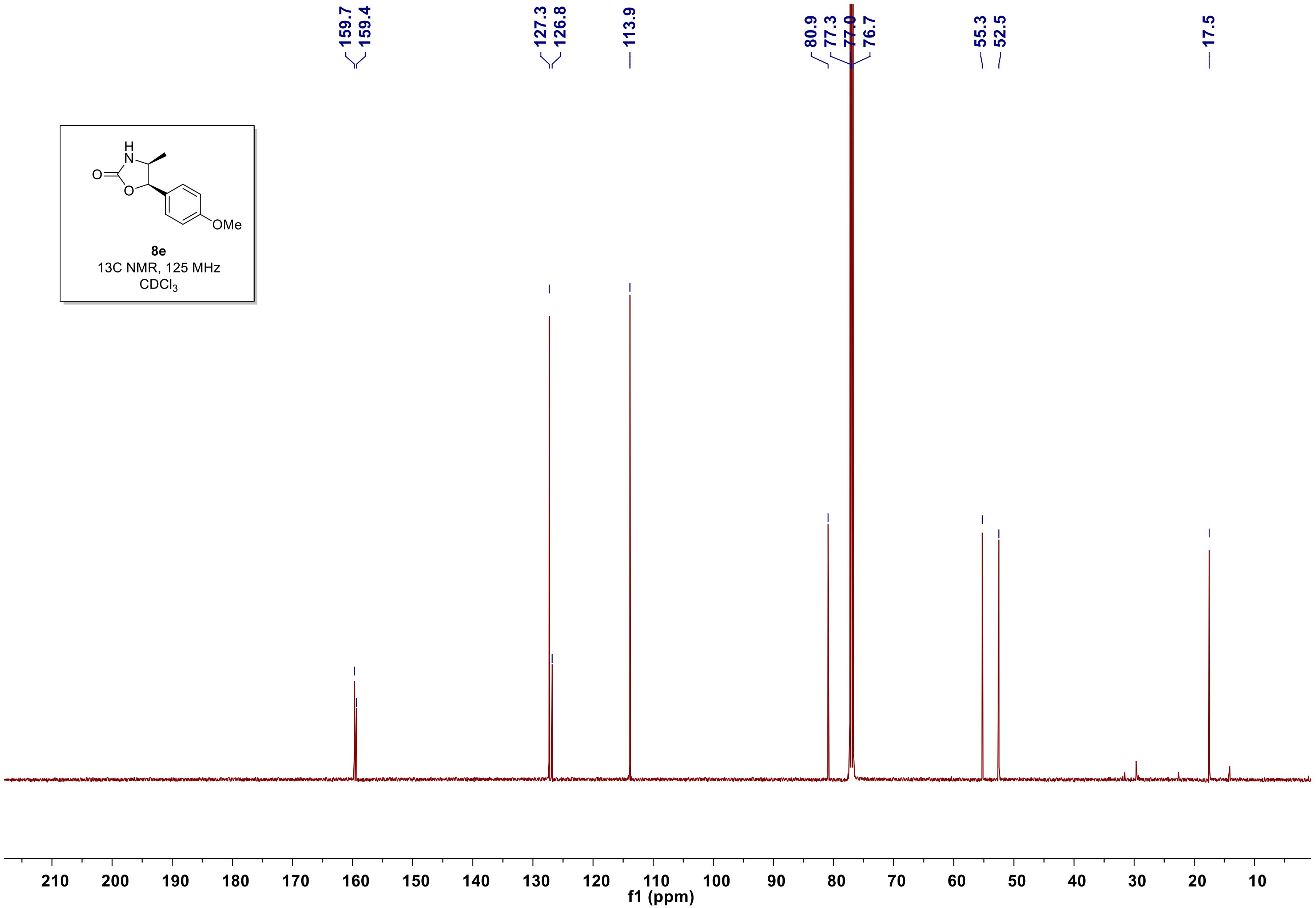
159.7
159.4
127.3
126.8
113.9
80.9
77.3
77.0
76.7
55.3
52.5
17.5
H
N
O
O
OMe
8e
13C NMR, 125 MHz
CDCl3
210 200 190 180 170 160 150 140 130 120 110 100 90 80 70 60 50 40 30 20 10
f1 (ppm)

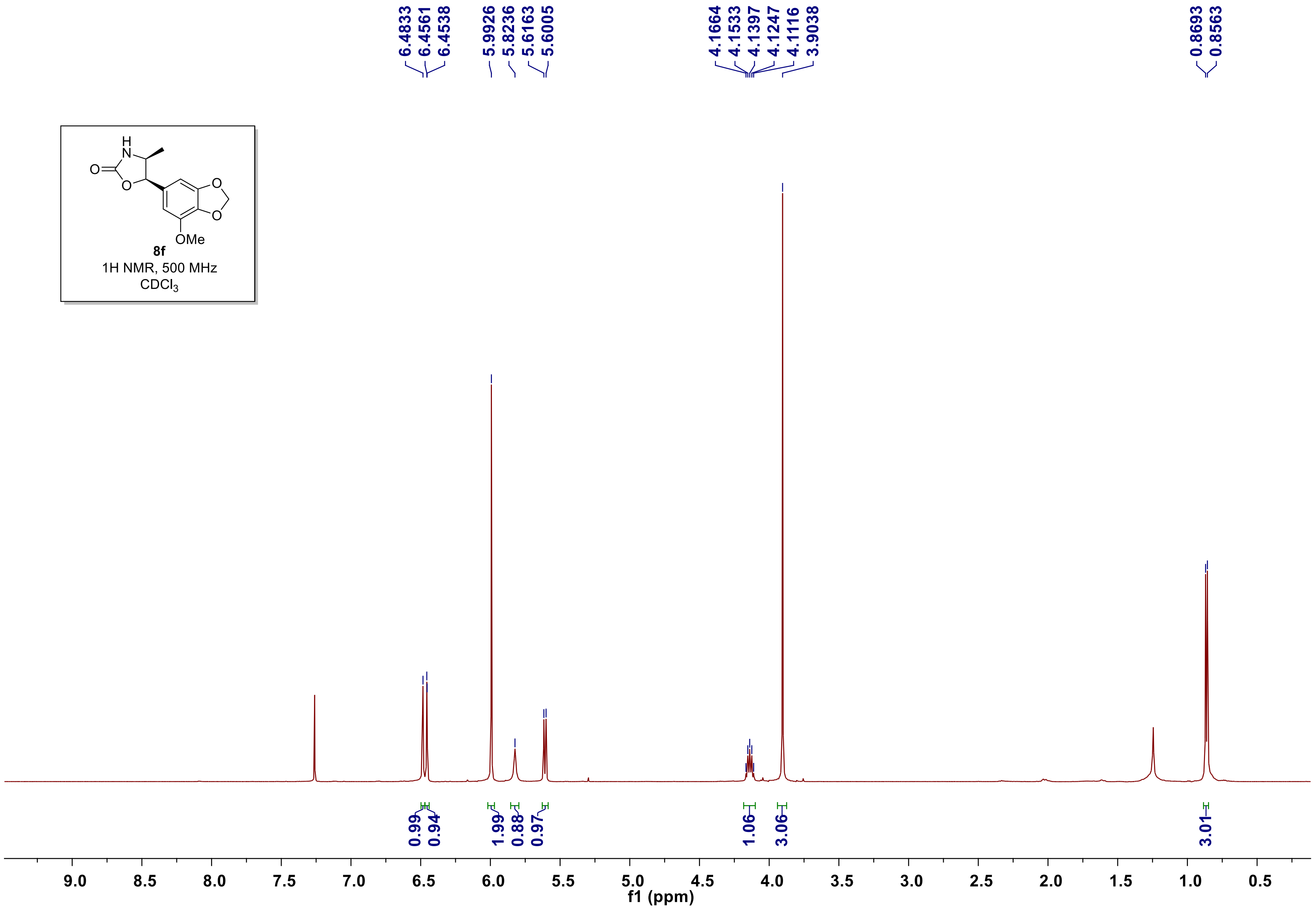
6.4833
6.4561
6.4538
5.9926
5.8236
5.6163
5.6005
4.1664
4.1533
4.1397
4.1247
4.1116
3.9038
0.8693
0.8563
8f
1H NMR, 500 MHz
CDCl3
0.99
0.94
1.99
0.88
0.97
1.06
3.06
3.01
9.0
8.5
8.0
7.5
7.0
6.5
6.0
5.5
5.0
4.5
4.0
3.5
3.0
2.5
2.0
1.5
1.0
0.5
f1 (ppm)

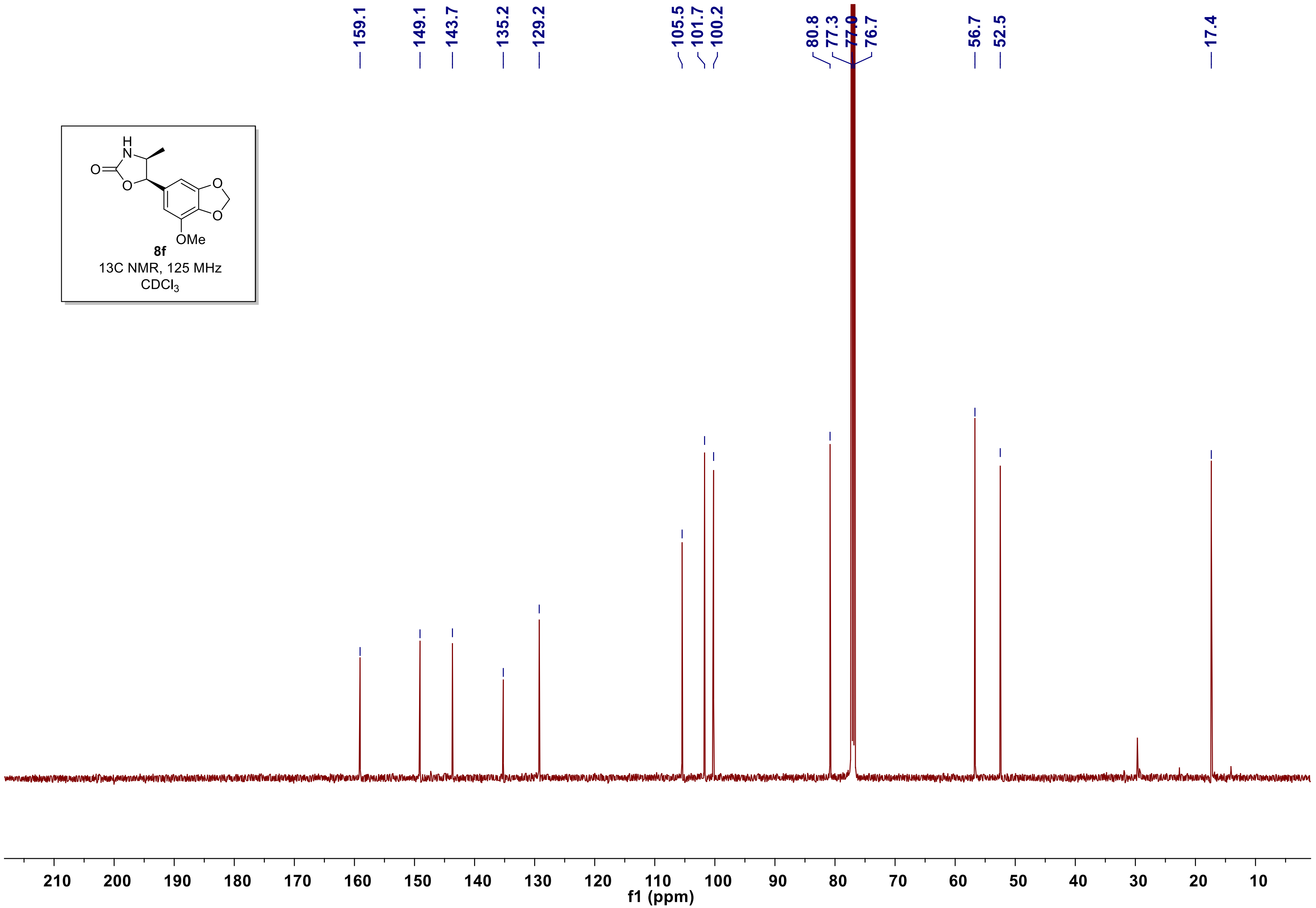
159.1
149.1
143.7
135.2
129.2
105.5
101.7
100.2
80.8
77.3
77.0
76.7
56.7
52.5
17.4
8f
13C NMR, 125 MHz
CDCl3
f1 (ppm)

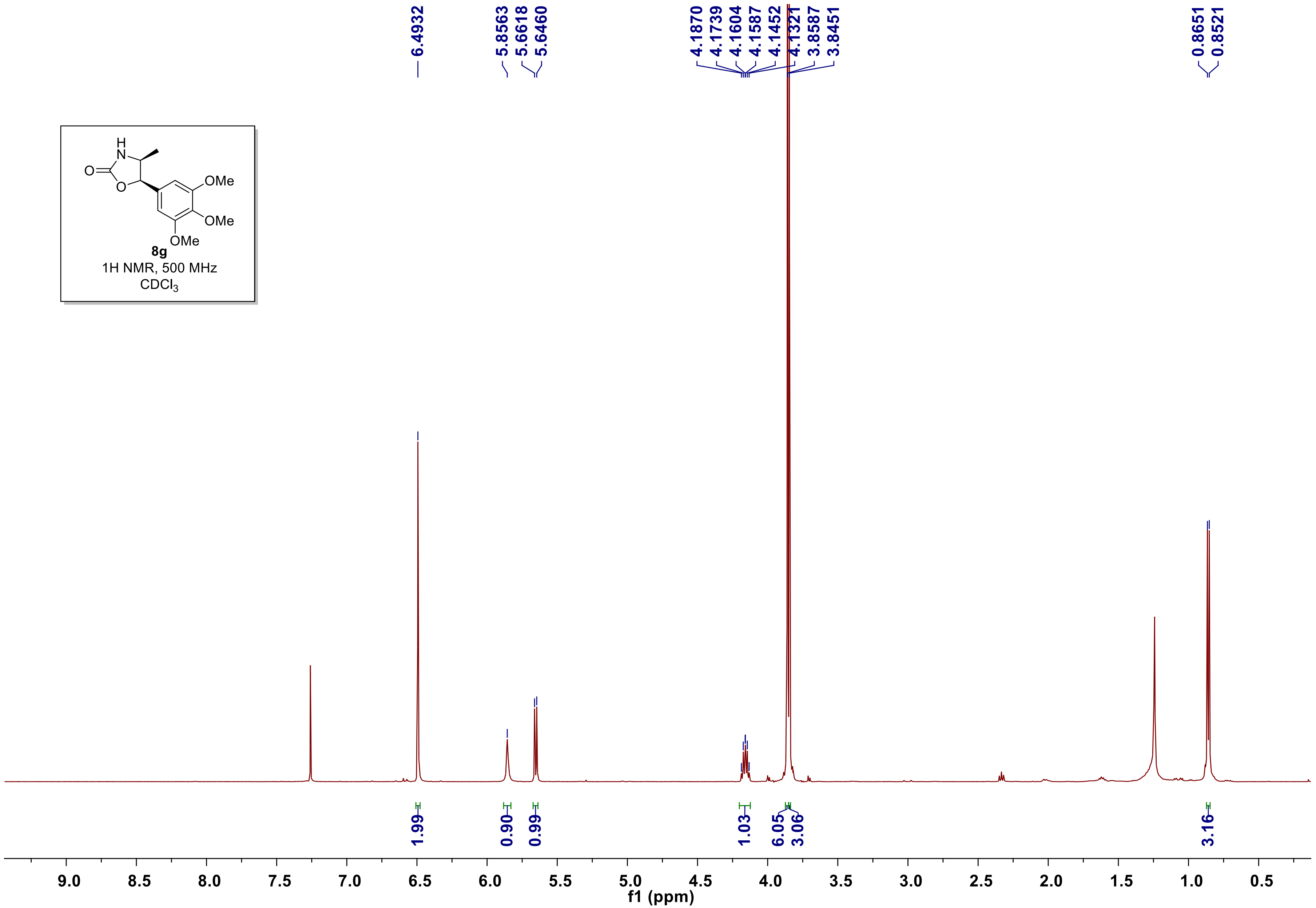
6.4932
5.8563
5.6618
5.6460
4.1870
4.1739
4.1604
4.1587
4.1452
4.1321
3.8587
3.8451
0.8651
0.8521
8g
1H NMR, 500 MHz
CDCl3
OMe
OMe
OMe
1.99
0.90
0.99
1.03
6.05
3.06
3.16
9.0
8.5
8.0
7.5
7.0
6.5
6.0
5.5
5.0
4.5
4.0
3.5
3.0
2.5
2.0
1.5
1.0
0.5
f1 (ppm)

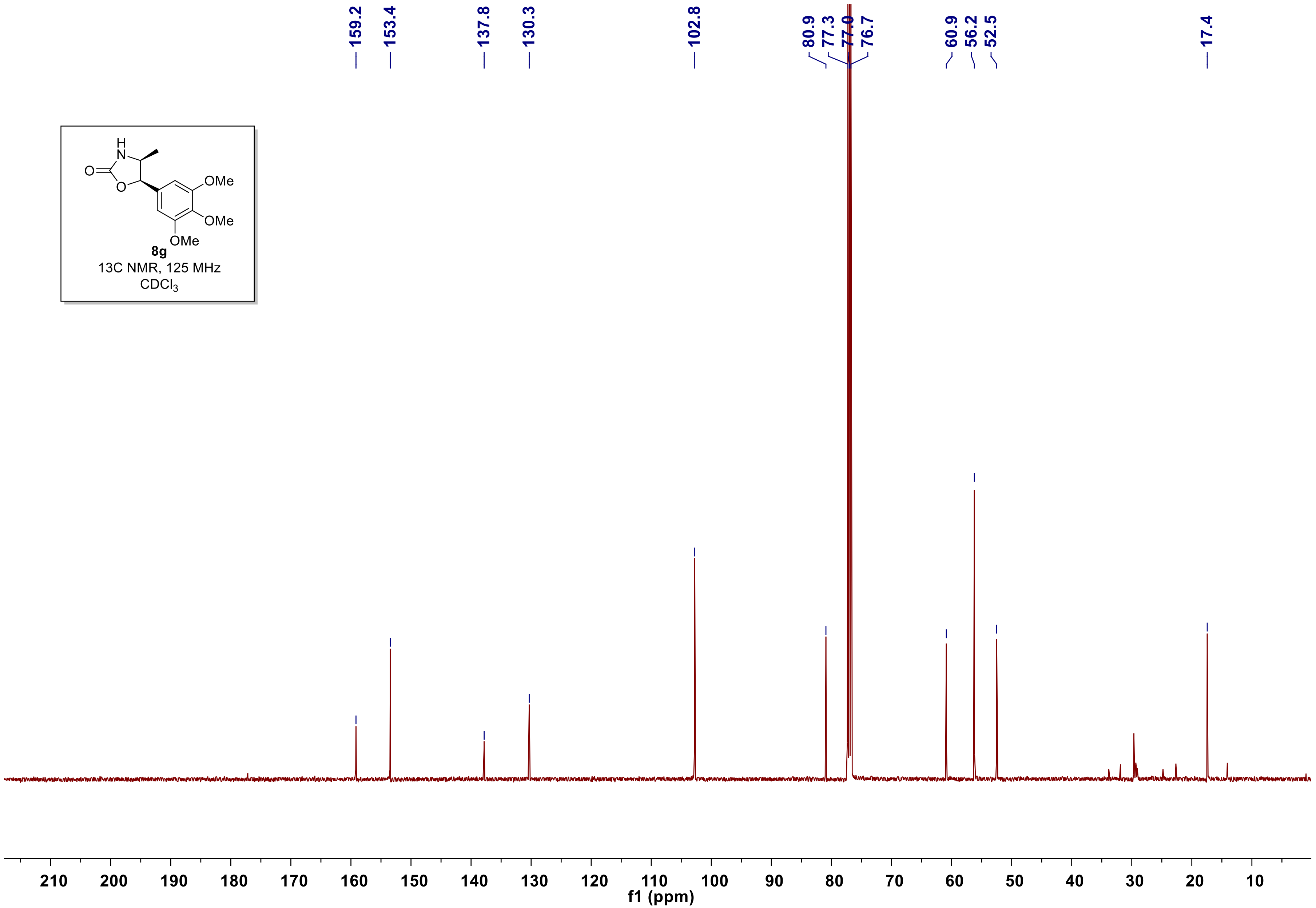
159.2
153.4
137.8
130.3
102.8
80.9
77.3
77.0
76.7
60.9
56.2
52.5
17.4
OMe
OMe
OMe
8g
13C NMR, 125 MHz
CDCl3
210 200 190 180 170 160 150 140 130 120 110 100 90 80 70 60 50 40 30 20 10
f1 (ppm)

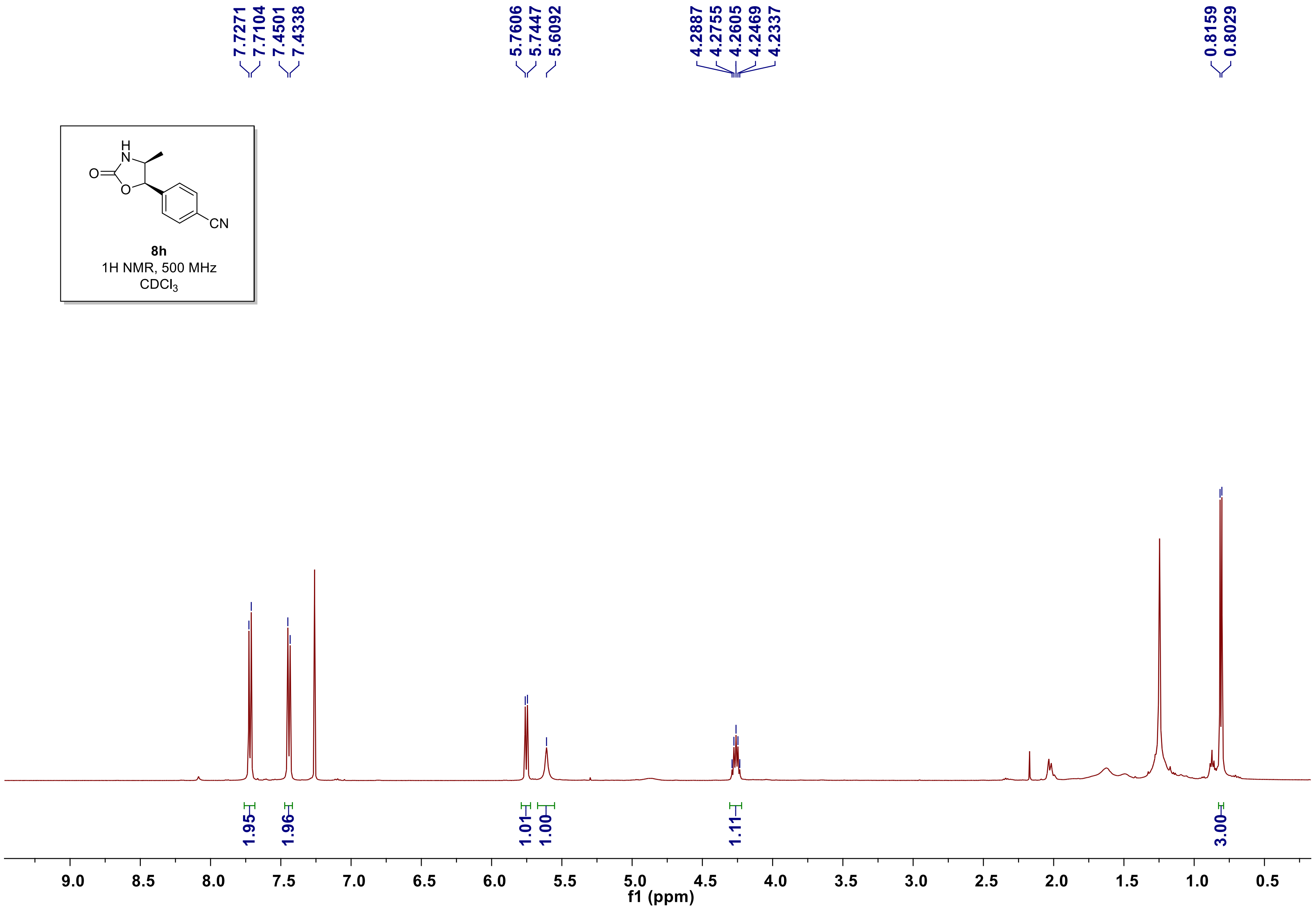

7.7271
7.7104
7.4501
7.4338
5.7606
5.7447
5.6092
4.2887
4.2755
4.2605
4.2469
4.2337
0.8159
0.8029
H
N
O
O
CN
8h
1H NMR, 500 MHz
CDCl3
1.95
1.96
1.01
1.00
1.11
3.00
9.0
8.5
8.0
7.5
7.0
6.5
6.0
5.5
5.0
4.5
4.0
3.5
3.0
2.5
2.0
1.5
1.0
0.5
f1 (ppm)

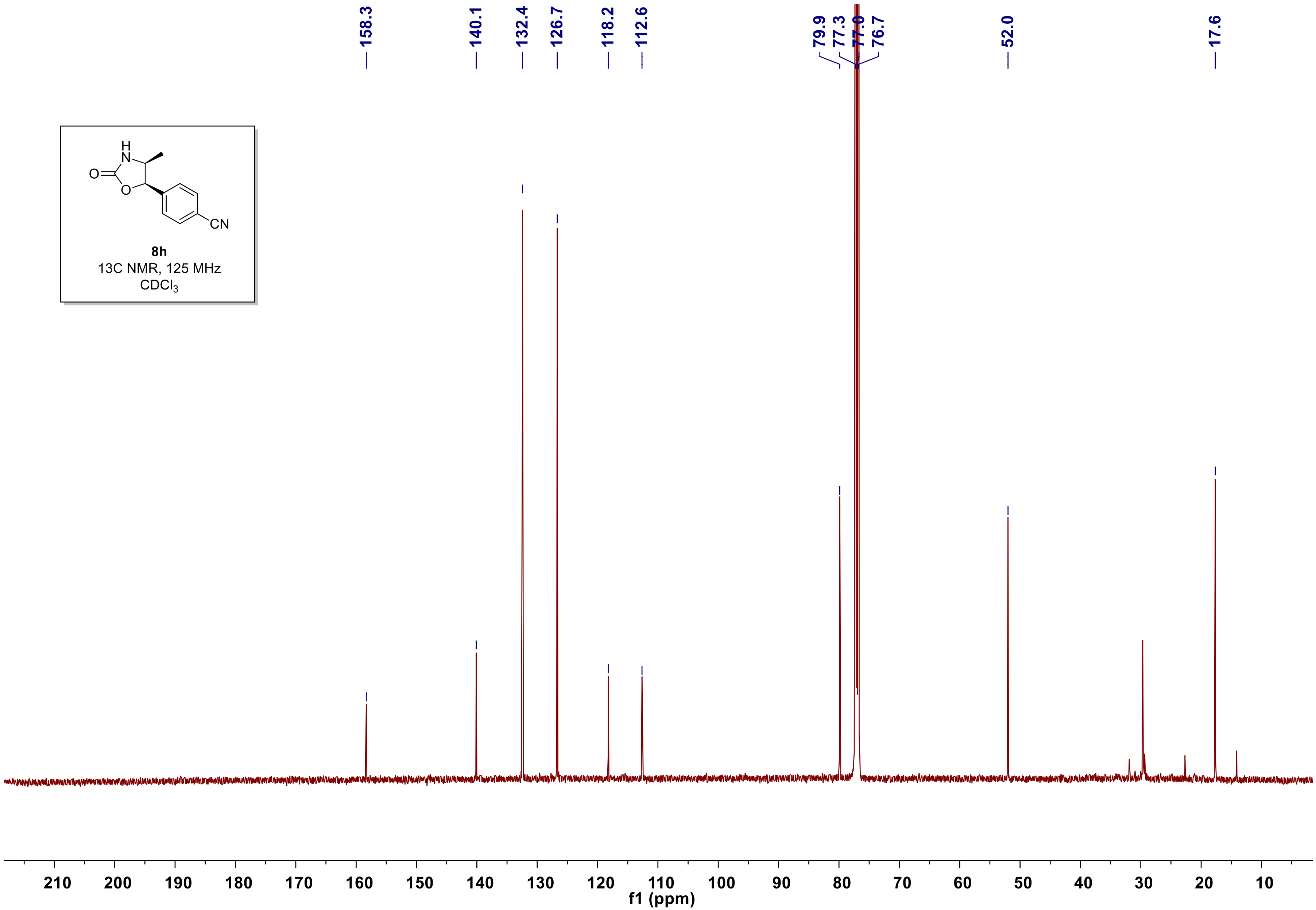

8h
13C NMR, 125 MHz
$CDCl_3$
158.3
140.1
132.4
126.7
118.2
112.6
79.9
77.3
77.0
76.7
52.0
17.6
210 200 190 180 170 160 150 140 130 120 110 100 90 80 70 60 50 40 30 20 10
f1 (ppm)

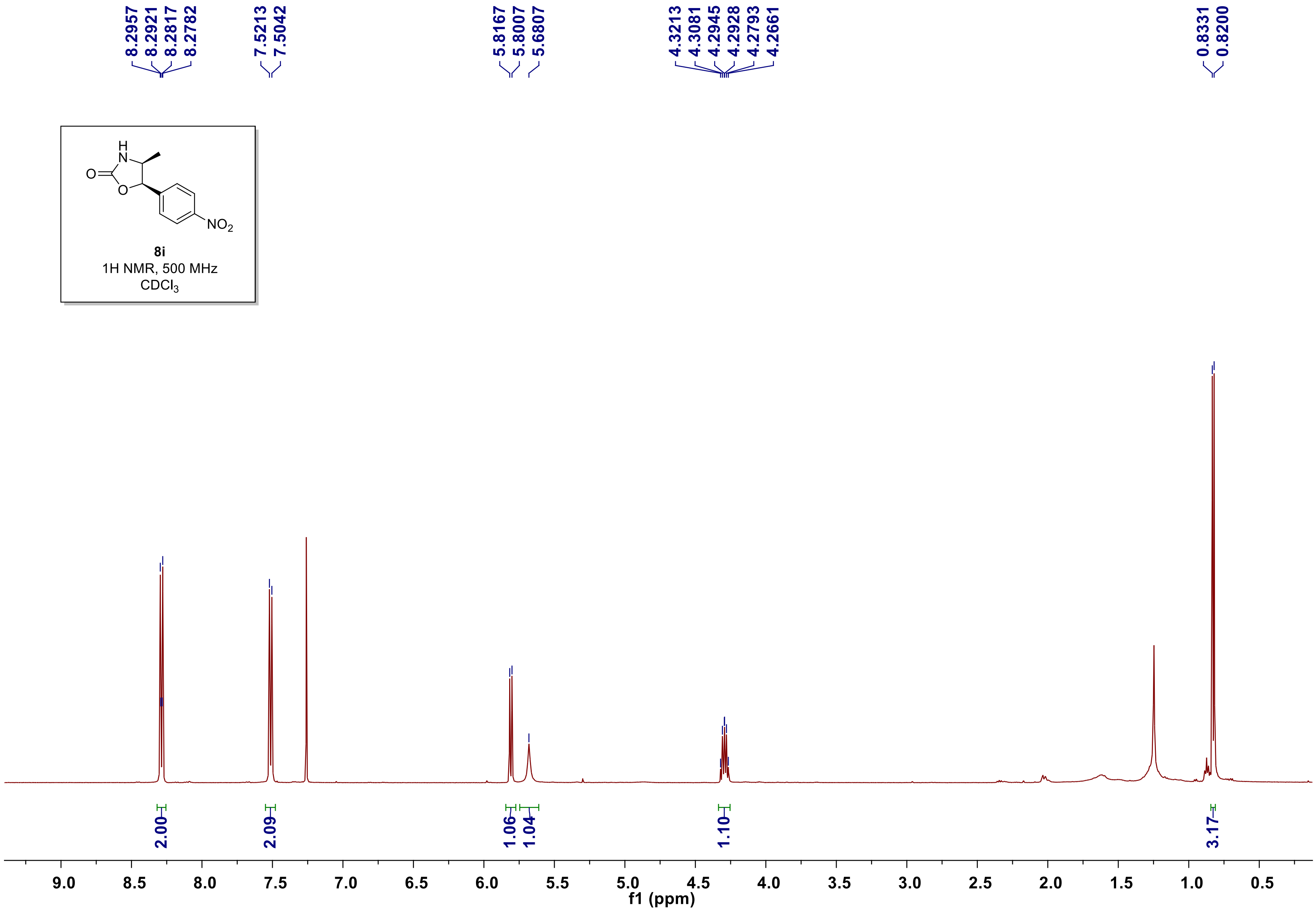
8.2957
8.2921
8.2817
8.2782
7.5213
7.5042
5.8167
5.8007
5.6807
4.3213
4.3081
4.2945
4.2928
4.2793
4.2661
0.8331
0.8200
8i
1H NMR, 500 MHz
CDCl3
2.00
2.09
1.06
1.04
1.10
3.17
9.0 8.5 8.0 7.5 7.0 6.5 6.0 5.5 5.0 4.5 4.0 3.5 3.0 2.5 2.0 1.5 1.0 0.5
f1 (ppm)

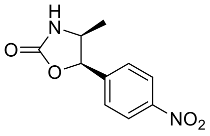

8i
13C NMR, 125 MHz
$CDCl_3$

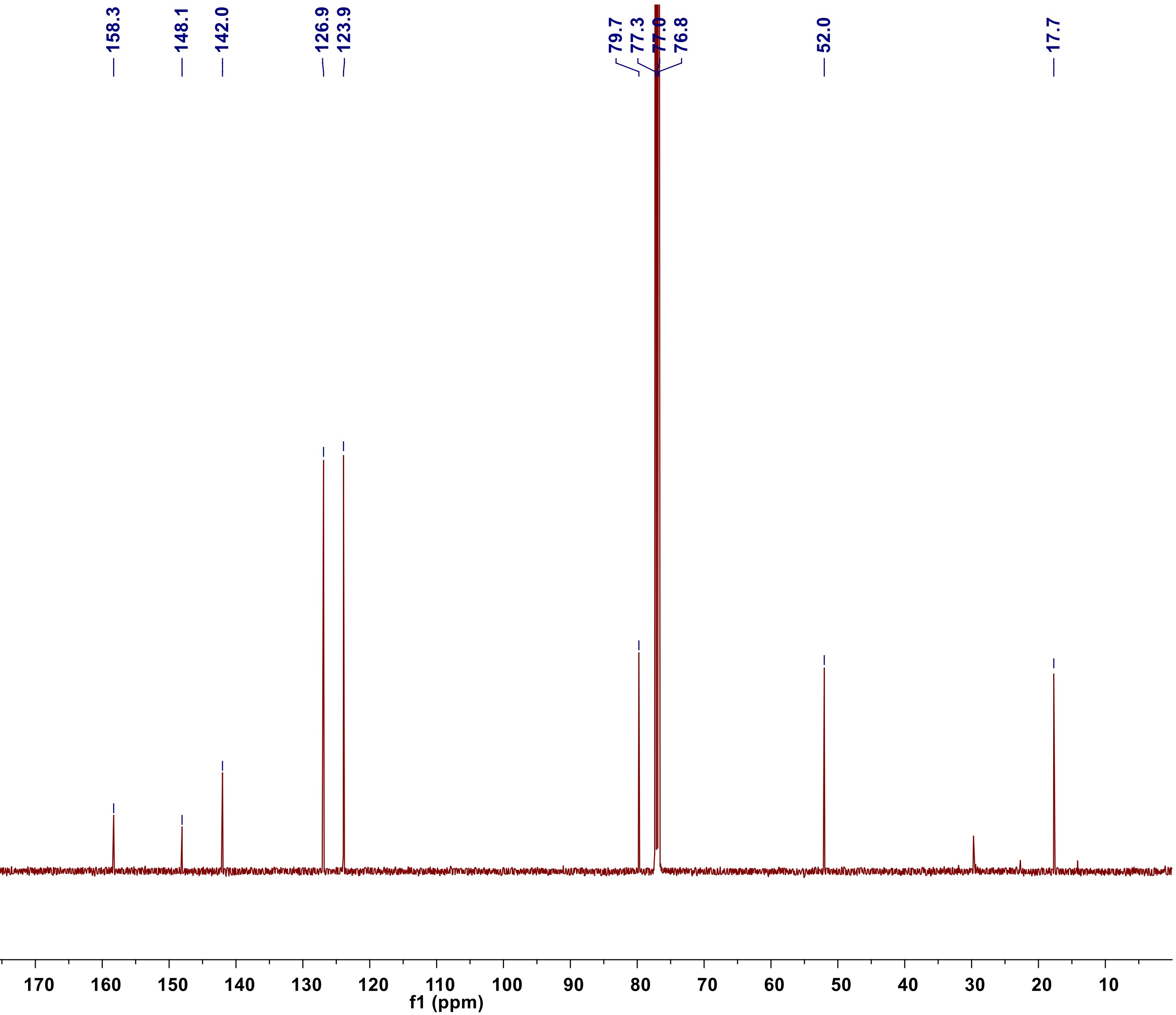

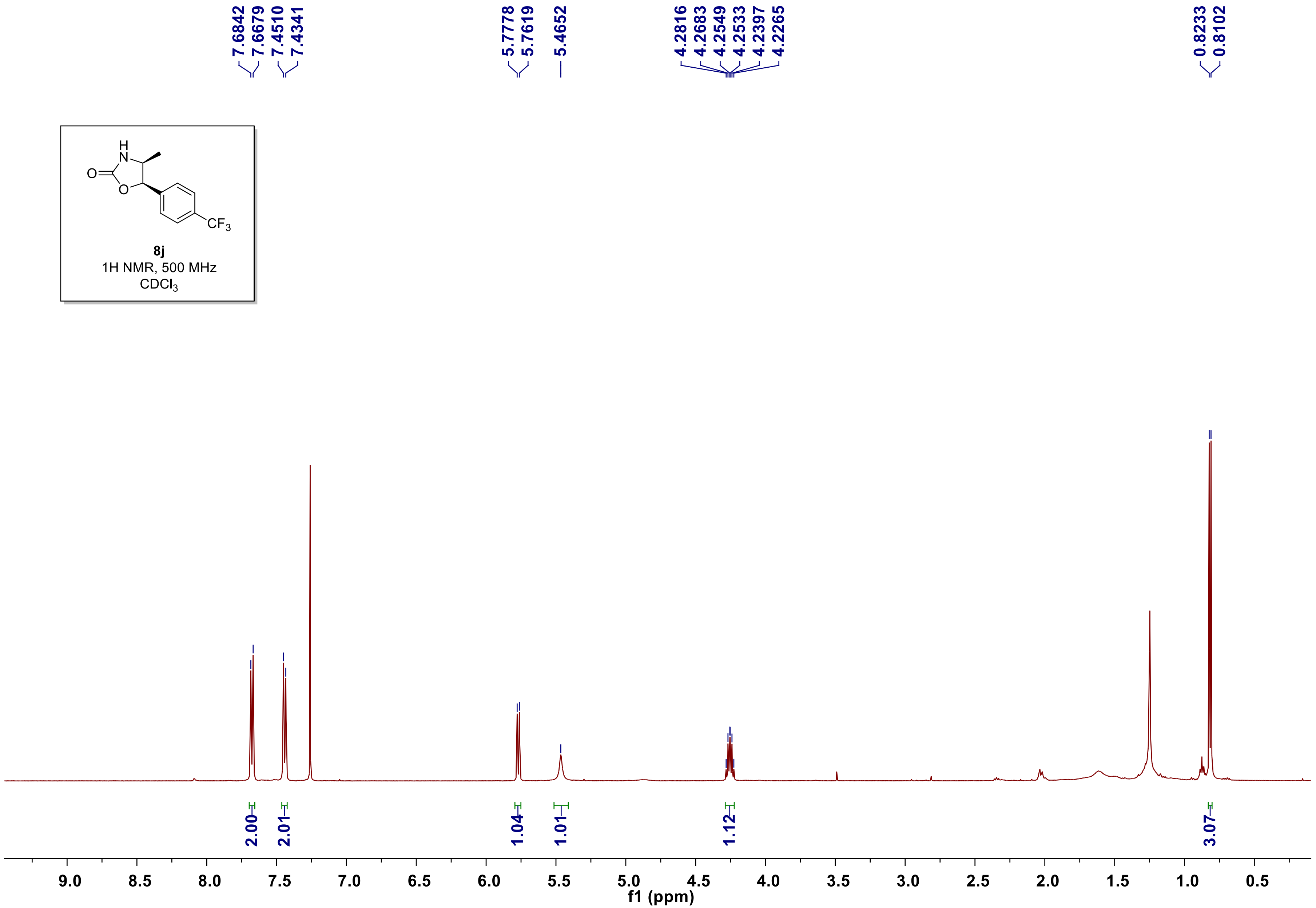
7.6842
7.6679
7.4510
7.4341
5.7778
5.7619
5.4652
4.2816
4.2683
4.2549
4.2533
4.2397
4.2265
0.8233
0.8102
8j
1H NMR, 500 MHz
CDCl3
2.00
2.01
1.04
1.01
1.12
3.07
9.0
8.5
8.0
7.5
7.0
6.5
6.0
5.5
5.0
4.5
4.0
3.5
3.0
2.5
2.0
1.5
1.0
0.5
f1 (ppm)

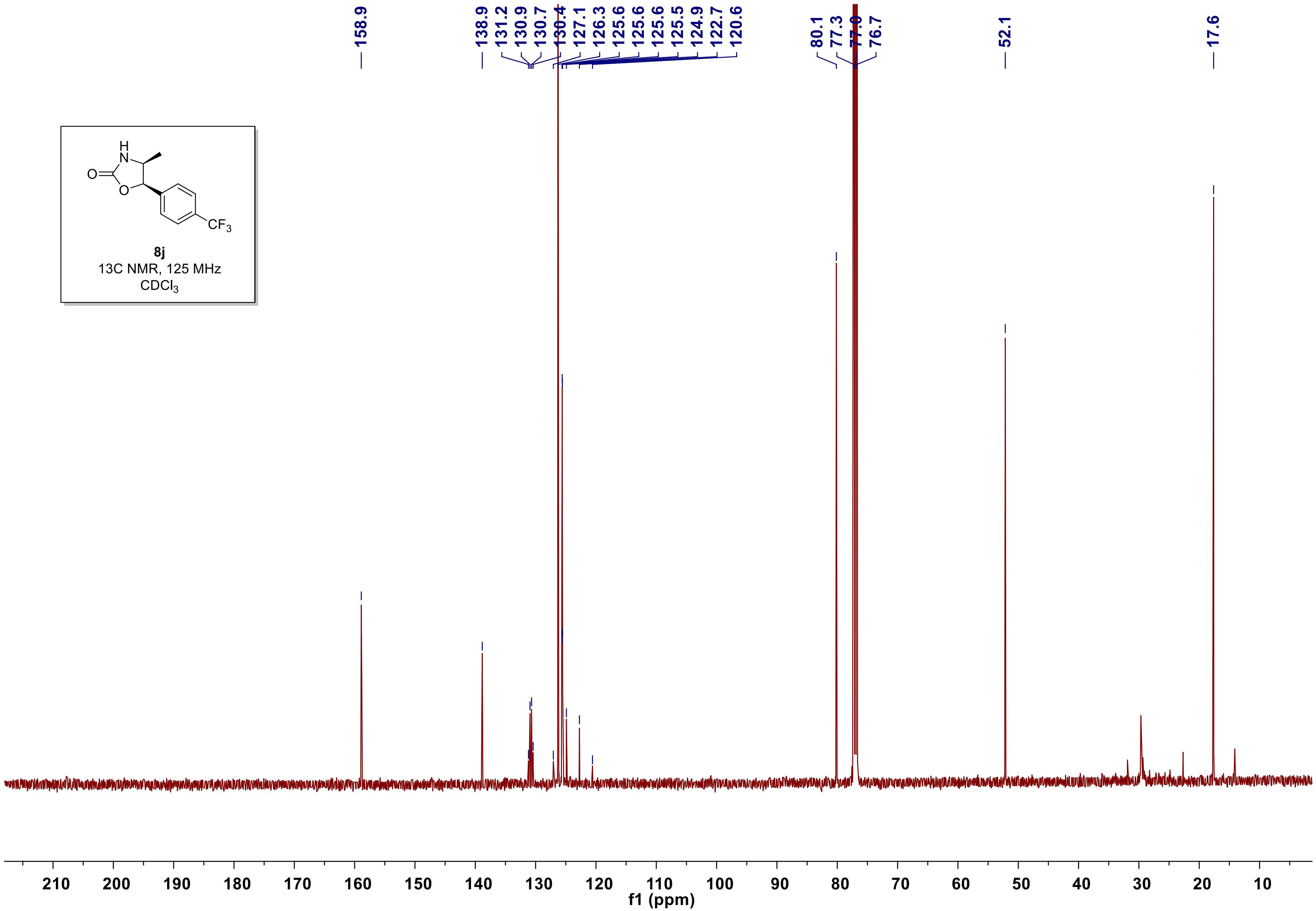

158.9
138.9
131.2
130.9
130.7
130.4
127.1
126.3
125.6
125.6
125.6
125.5
124.9
122.7
120.6
80.1
77.3
77.0
76.7
52.1
17.6
8j
13C NMR, 125 MHz
$CDCl_3$
210 200 190 180 170 160 150 140 130 120 110 100 90 80 70 60 50 40 30 20 10
f1 (ppm)

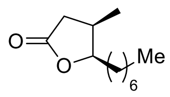

8k
1H NMR, 500 MHz
$CDCl_3$

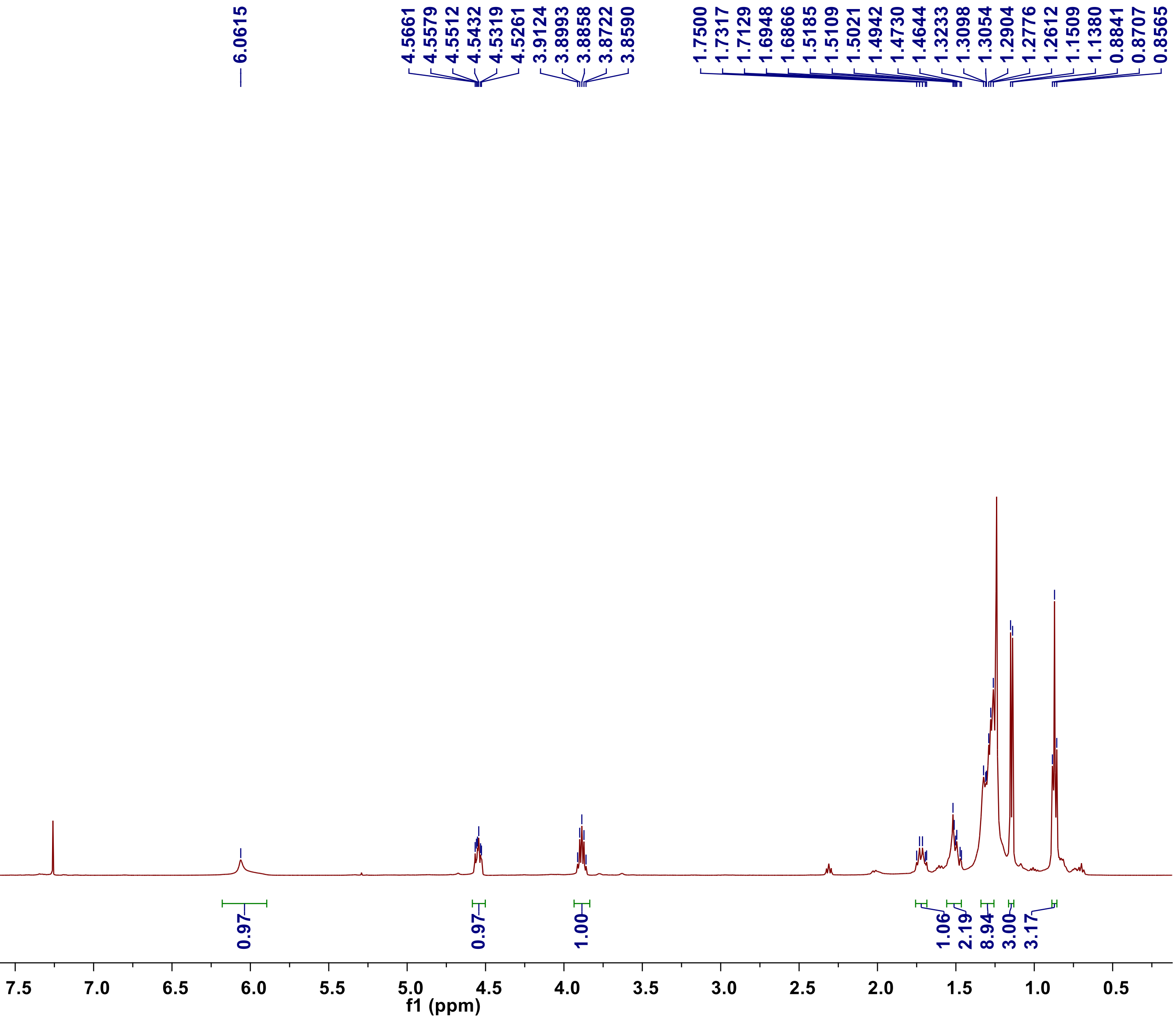

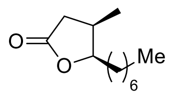

8k
13C NMR, 125 MHz
$CDCl_3$

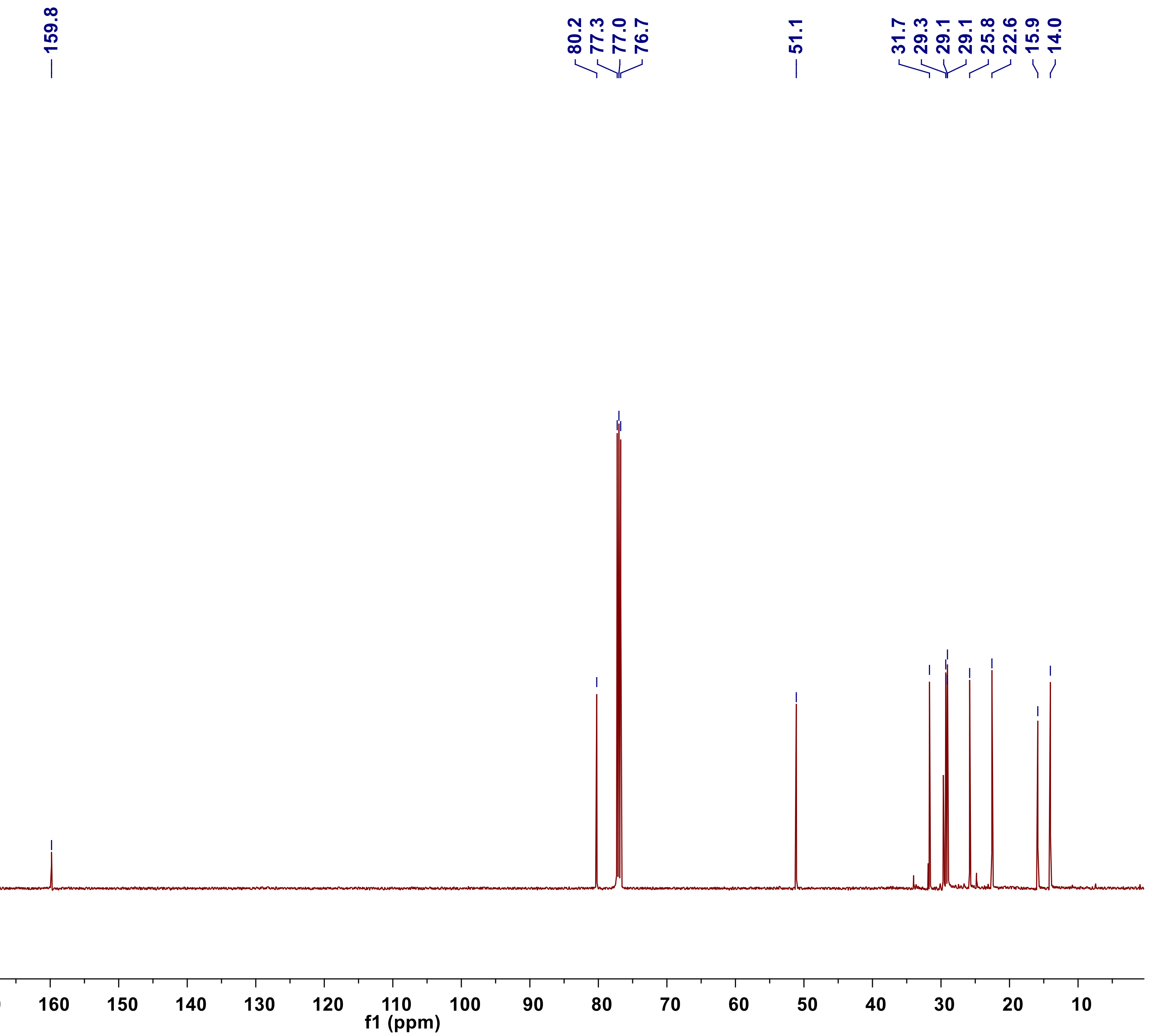

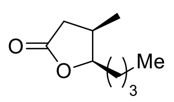

8l
1H NMR, 500 MHz
$CDCl_3$

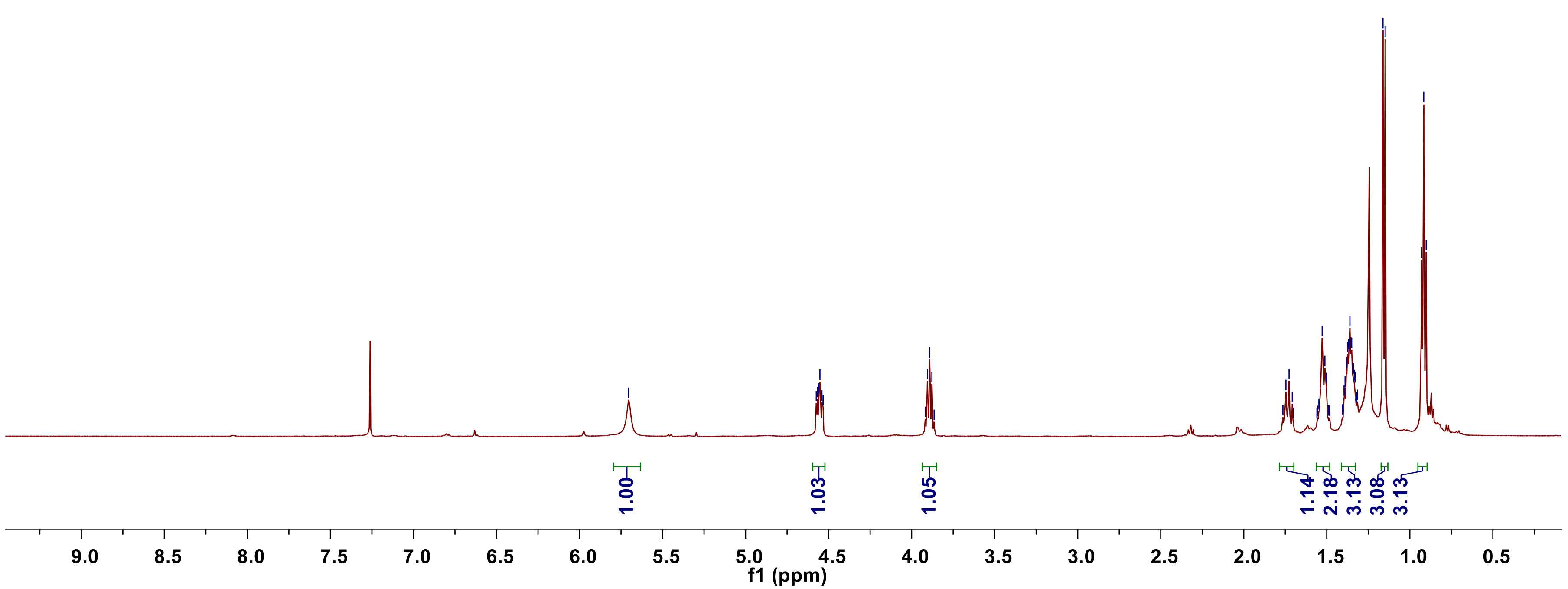

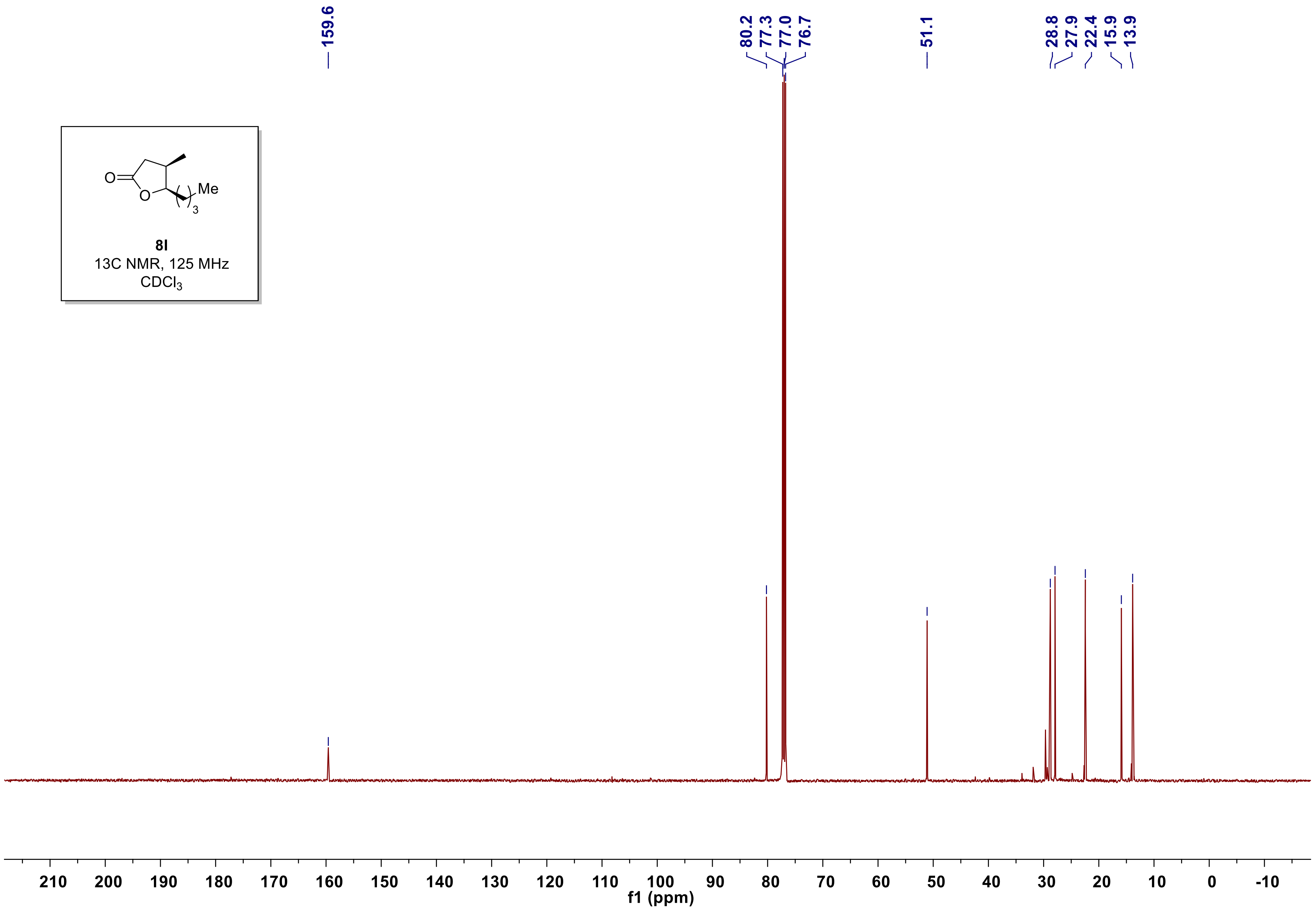

159.6
80.2
77.3
77.0
76.7
51.1
28.8
27.9
22.4
15.9
13.9
O
O
Me
3
8l
13C NMR, 125 MHz
CDCl3
210 200 190 180 170 160 150 140 130 120 110 100 90 80 70 60 50 40 30 20 10 0 -10
f1 (ppm)

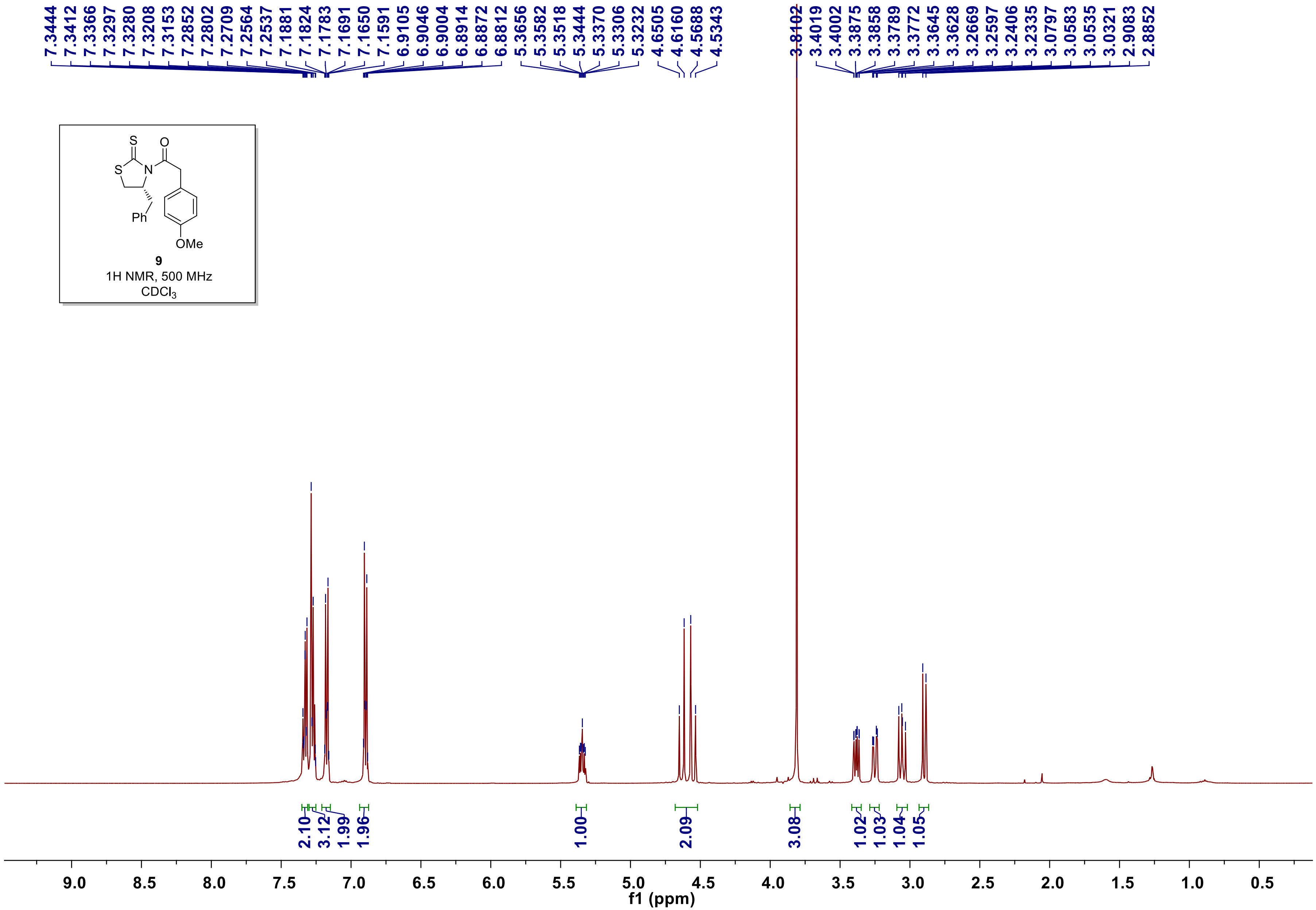

7.3444
7.3412
7.3366
7.3297
7.3280
7.3208
7.3153
7.2852
7.2802
7.2709
7.2564
7.2537
7.1881
7.1824
7.1783
7.1691
7.1650
7.1591
6.9105
6.9046
6.9004
6.8914
6.8872
6.8812
5.3656
5.3582
5.3518
5.3444
5.3370
5.3306
5.3232
4.6505
4.6160
4.5688
4.5343
3.8102
3.4019
3.4002
3.3875
3.3858
3.3789
3.3772
3.3645
3.3628
3.2669
3.2597
3.2406
3.2335
3.0797
3.0583
3.0535
3.0321
2.9083
2.8852
S
O
S
N
Ph
OMe
9
1H NMR, 500 MHz
$CDCl_3$
2.10
3.12
1.99
1.96
1.00
2.09
3.08
1.02
1.03
1.04
1.05
9.0
8.5
8.0
7.5
7.0
6.5
6.0
5.5
5.0
4.5
4.0
3.5
3.0
2.5
2.0
1.5
1.0
0.5
f1 (ppm)

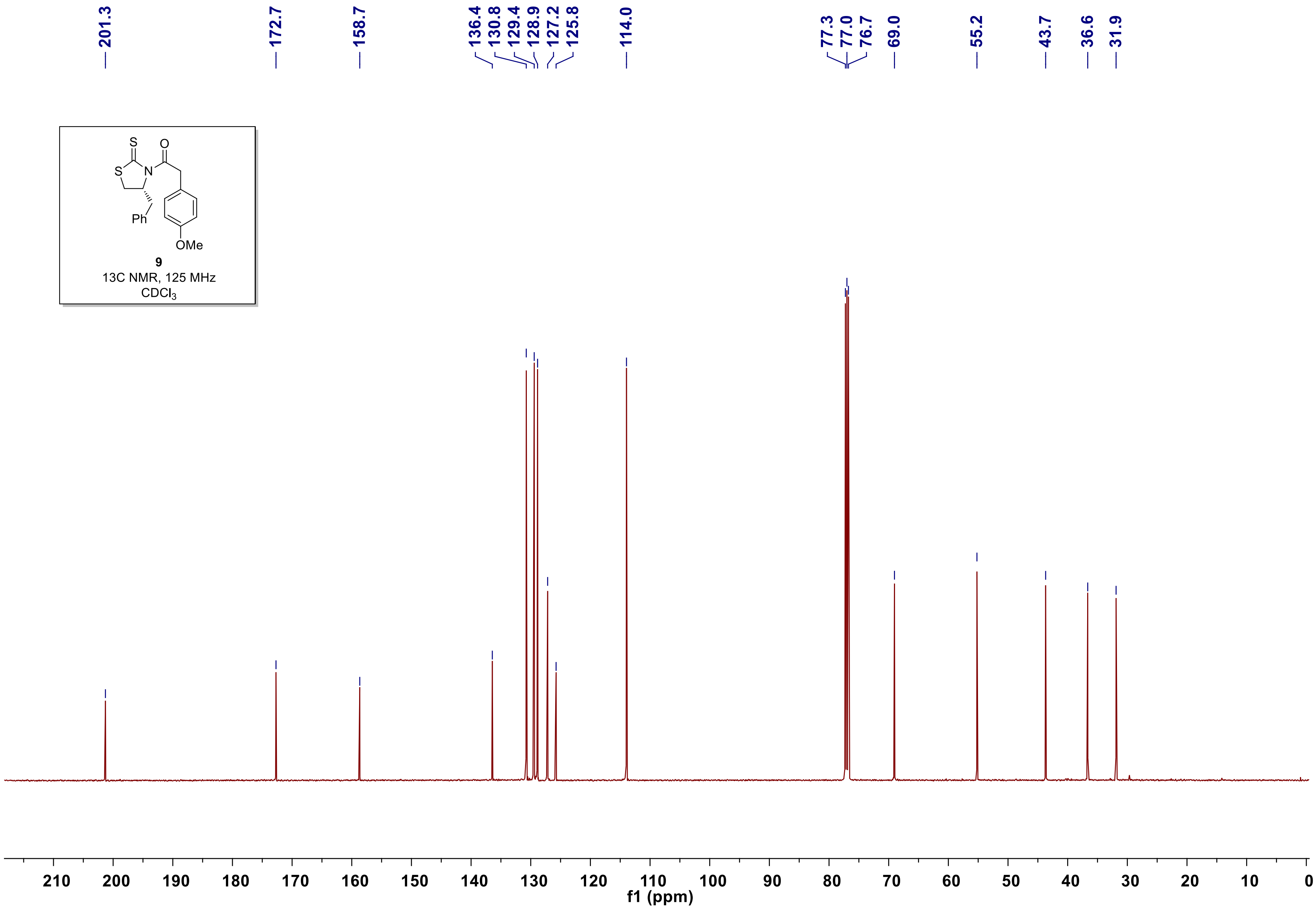
201.3
172.7
158.7
136.4
130.8
129.4
128.9
127.2
125.8
114.0
77.3
77.0
76.7
69.0
55.2
43.7
36.6
31.9
S
O
S
N
Ph
OMe
9
13C NMR, 125 MHz
CDCl3
210 200 190 180 170 160 150 140 130 120 110 100 90 80 70 60 50 40 30 20 10 0
f1 (ppm)

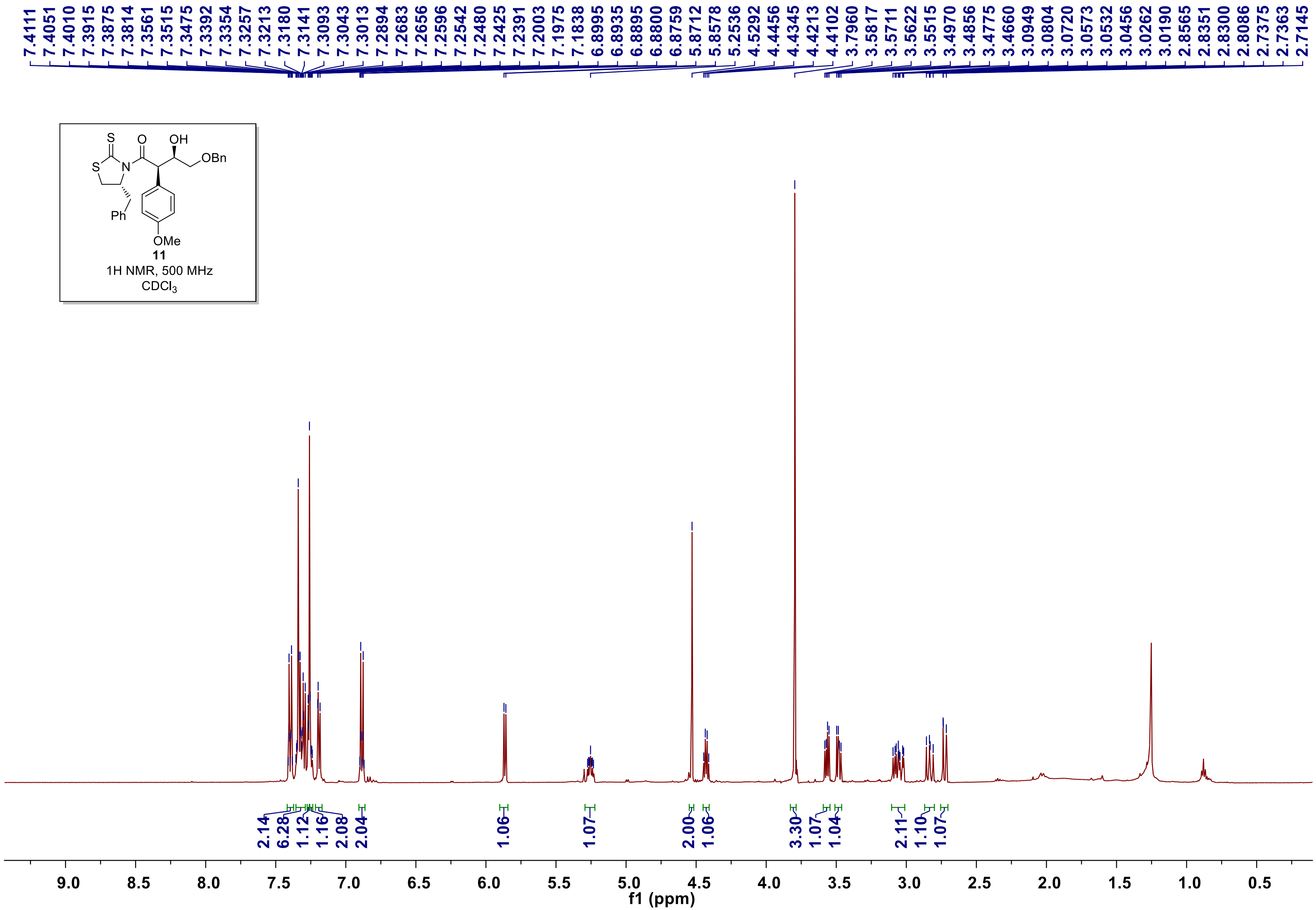
7.4111 7.4051 7.4010 7.3915 7.3875 7.3814 7.3561 7.3515 7.3475 7.3392 7.3354 7.3257 7.3213 7.3180 7.3141 7.3093 7.3043 7.3013 7.2894 7.2683 7.2656 7.2596 7.2542 7.2480 7.2425 7.2391 7.2003 7.1975 7.1838 6.8995 6.8935 6.8895 6.8800 6.8759 5.8712 5.8578 5.2536 4.5292 4.4456 4.4345 4.4213 4.4102 3.7960 3.5817 3.5711 3.5622 3.5515 3.4970 3.4856 3.4775 3.4660 3.0949 3.0804 3.0720 3.0573 3.0532 3.0456 3.0262 3.0190 2.8565 2.8351 2.8300 2.8086 2.7375 2.7363 2.7145
S
O
OH
OBn
S
N
Ph
OMe
11
1H NMR, 500 MHz
$CDCl_3$
2.14 6.28 1.12 1.16 2.08 2.04 1.06 1.07 2.00 1.06 3.30 1.07 1.04 2.11 1.10 1.07
9.0 8.5 8.0 7.5 7.0 6.5 6.0 5.5 5.0 4.5 4.0 3.5 3.0 2.5 2.0 1.5 1.0 0.5
f1 (ppm)

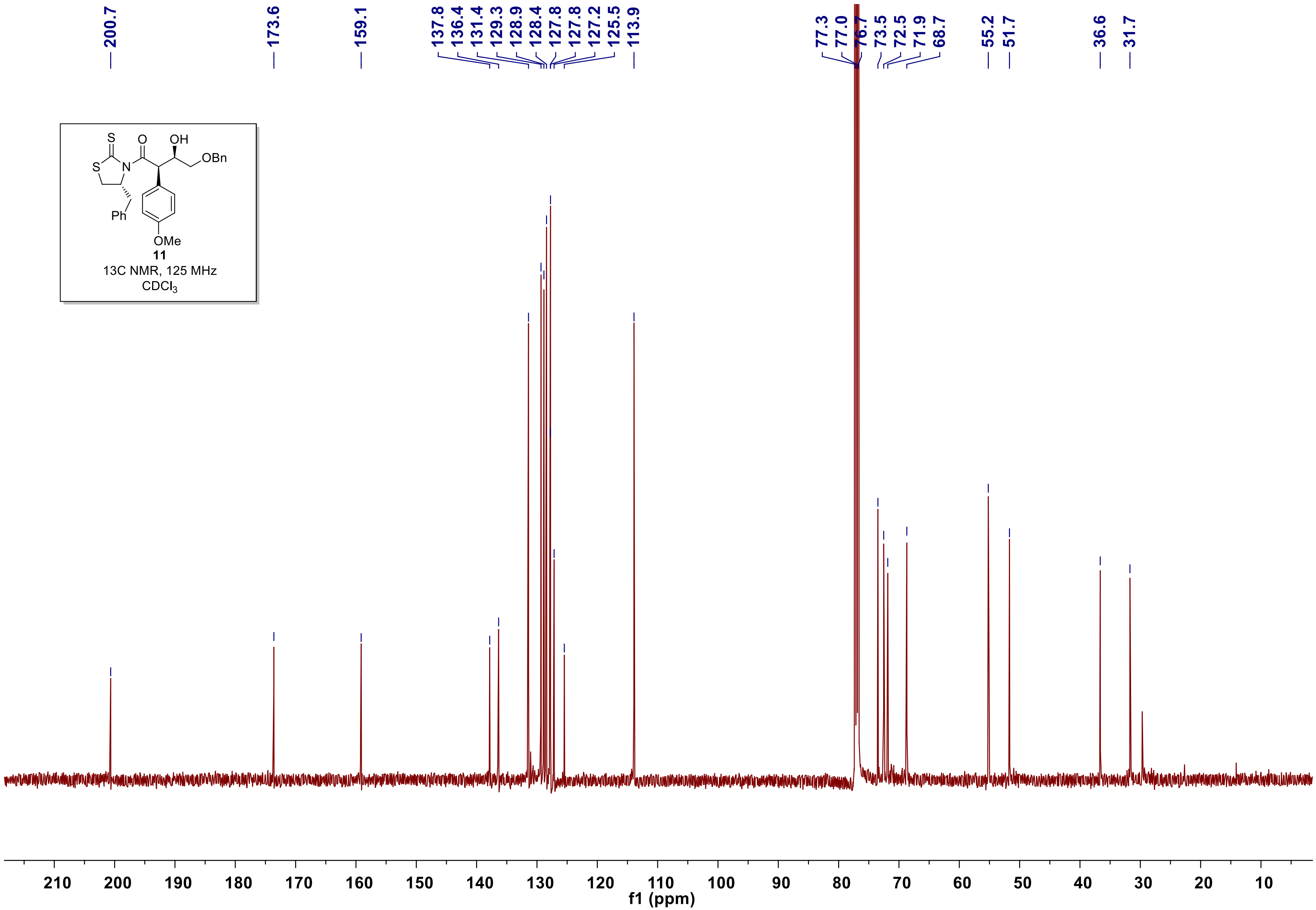
200.7
173.6
159.1
137.8
136.4
131.4
129.3
128.9
128.4
127.8
127.8
127.2
125.5
113.9
77.3
77.0
76.7
73.5
72.5
71.9
68.7
55.2
51.7
36.6
31.7
S
O
OH
OBn
S
N
Ph
OMe
11
13C NMR, 125 MHz
$CDCl_3$
210
200
190
180
170
160
150
140
130
120
110
100
90
80
70
60
50
40
30
20
10
f1 (ppm)

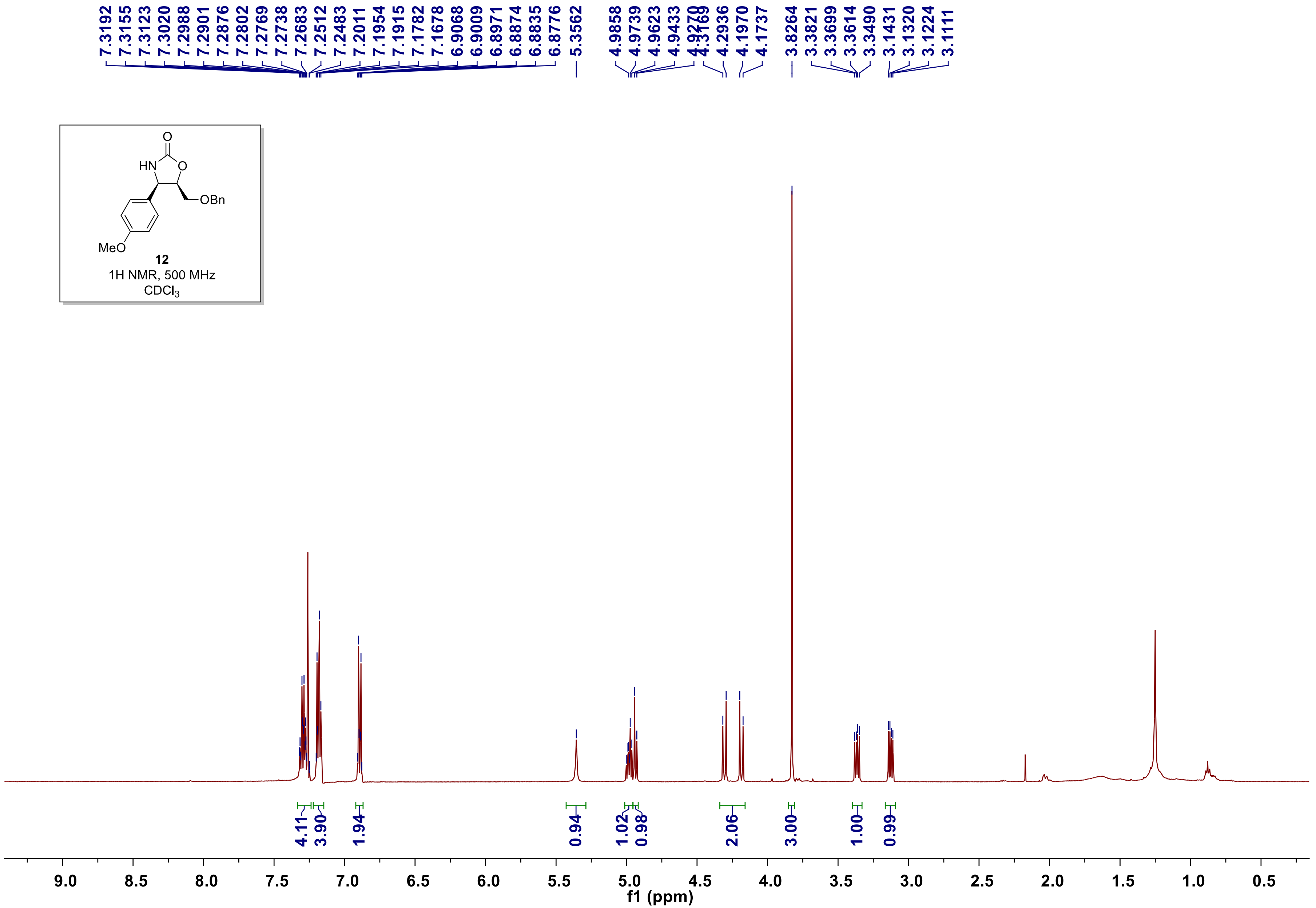

HN
O
O
OBn
MeO
12
1H NMR, 500 MHz
CDCl3
f1 (ppm)

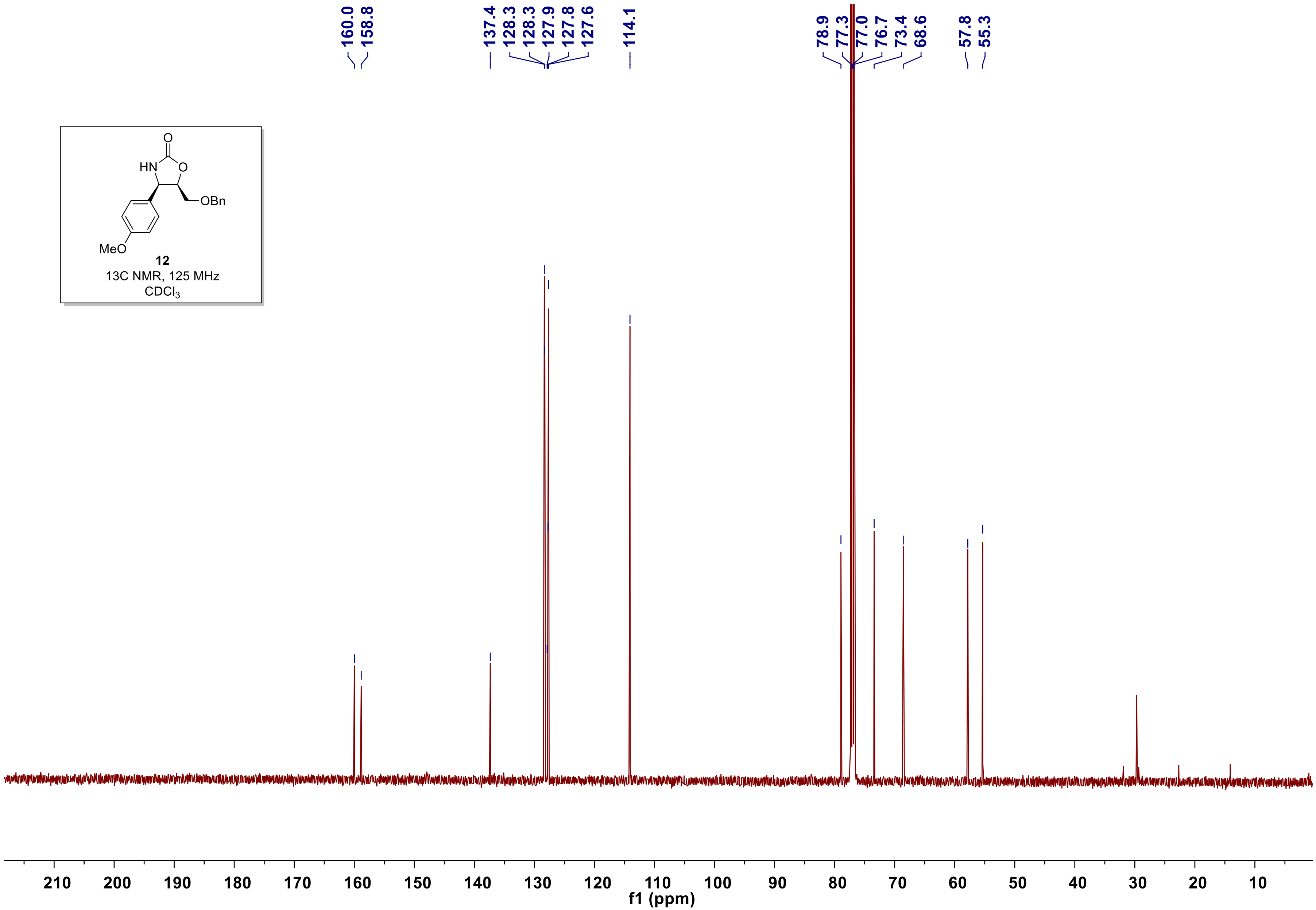
160.0
158.8
137.4
128.3
128.3
127.9
127.8
127.6
114.1
78.9
77.3
77.0
76.7
73.4
68.6
57.8
55.3
O
HN
O
OBn
MeO
12
13C NMR, 125 MHz
CDCl3
210
200
190
180
170
160
150
140
130
120
110
100
90
80
70
60
50
40
30
20
10
f1 (ppm)

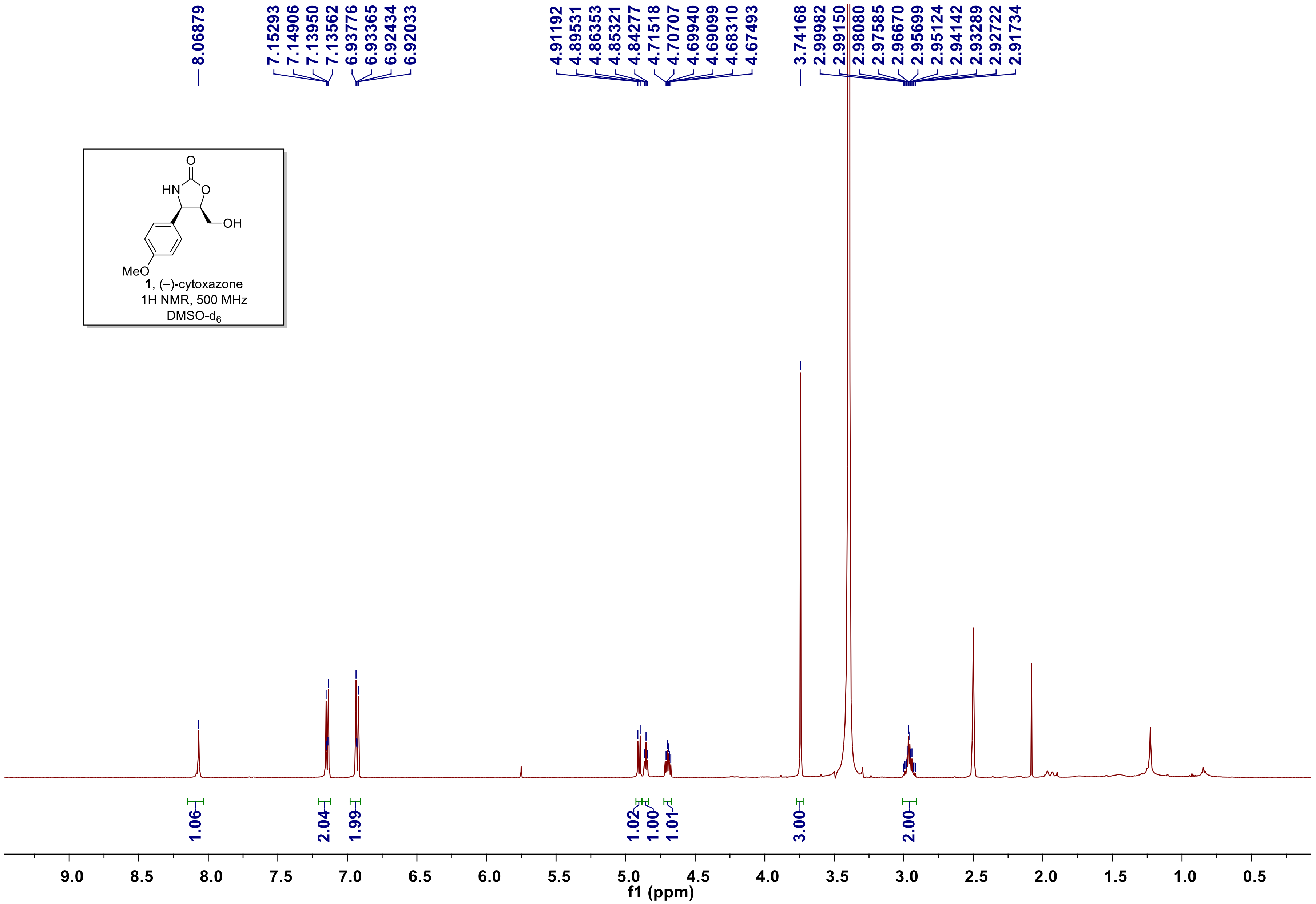

8.06879
7.15293
7.14906
7.13950
7.13562
6.93776
6.93365
6.92434
6.92033
4.91192
4.89531
4.86353
4.85321
4.84277
4.71518
4.70707
4.69940
4.69099
4.68310
4.67493
3.74168
2.99982
2.99150
2.98080
2.97585
2.96670
2.95699
2.95124
2.94142
2.93289
2.92722
2.91734
HN
O
O
OH
MeO
1, (−)-cytoxazone
1H NMR, 500 MHz
DMSO-d6
1.06
2.04
1.99
1.02
1.00
1.01
3.00
2.00
9.0
8.5
8.0
7.5
7.0
6.5
6.0
5.5
5.0
4.5
4.0
3.5
3.0
2.5
2.0
1.5
1.0
0.5
f1 (ppm)

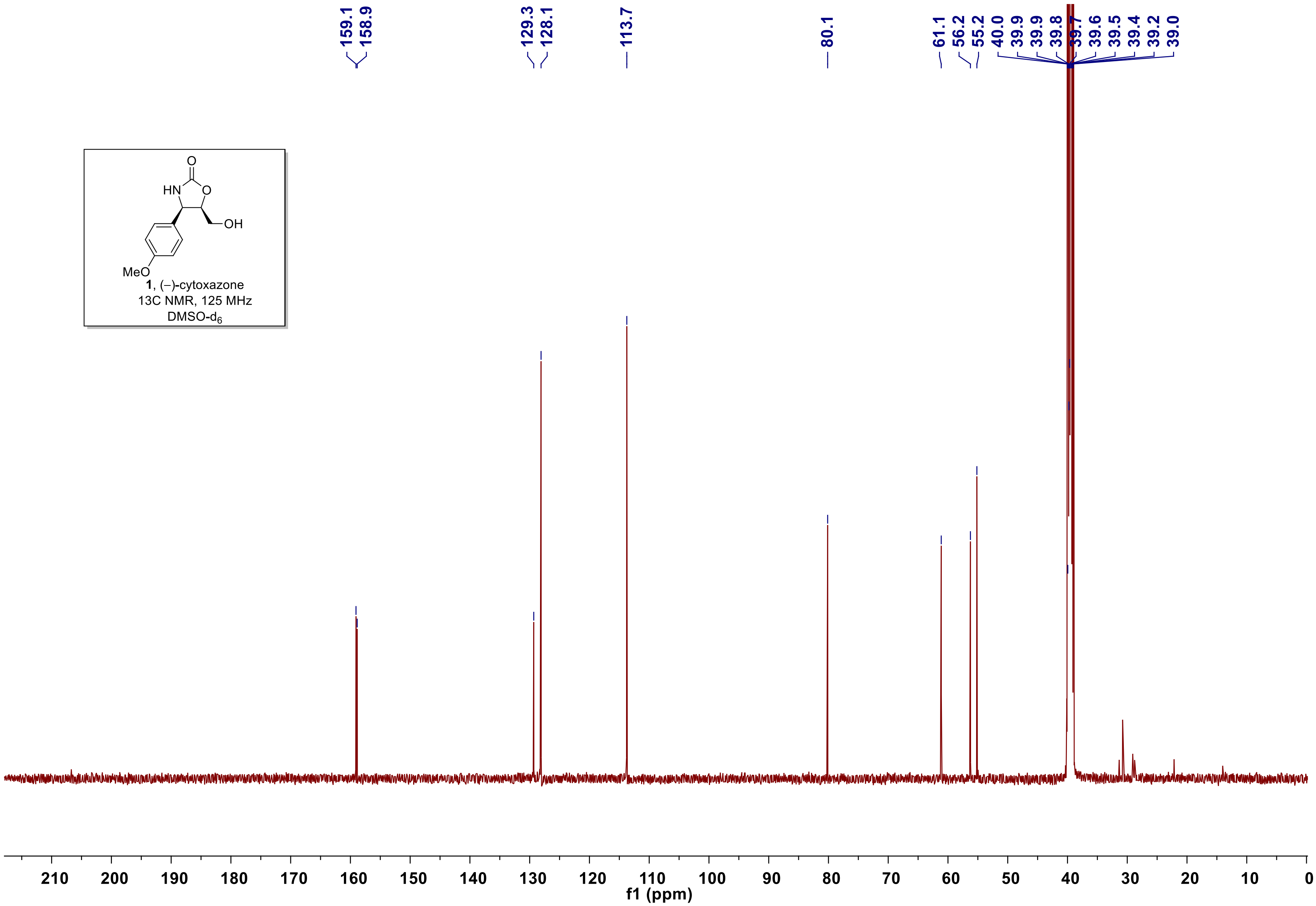

159.1
158.9
129.3
128.1
113.7
80.1
61.1
56.2
55.2
40.0
39.9
39.9
39.8
39.7
39.6
39.5
39.4
39.2
39.0
O
HN
O
OH
MeO
1, (−)-cytoxazone
13C NMR, 125 MHz
DMSO-d6
210
200
190
180
170
160
150
140
130
120
110
100
90
80
70
60
50
40
30
20
10
0
f1 (ppm)

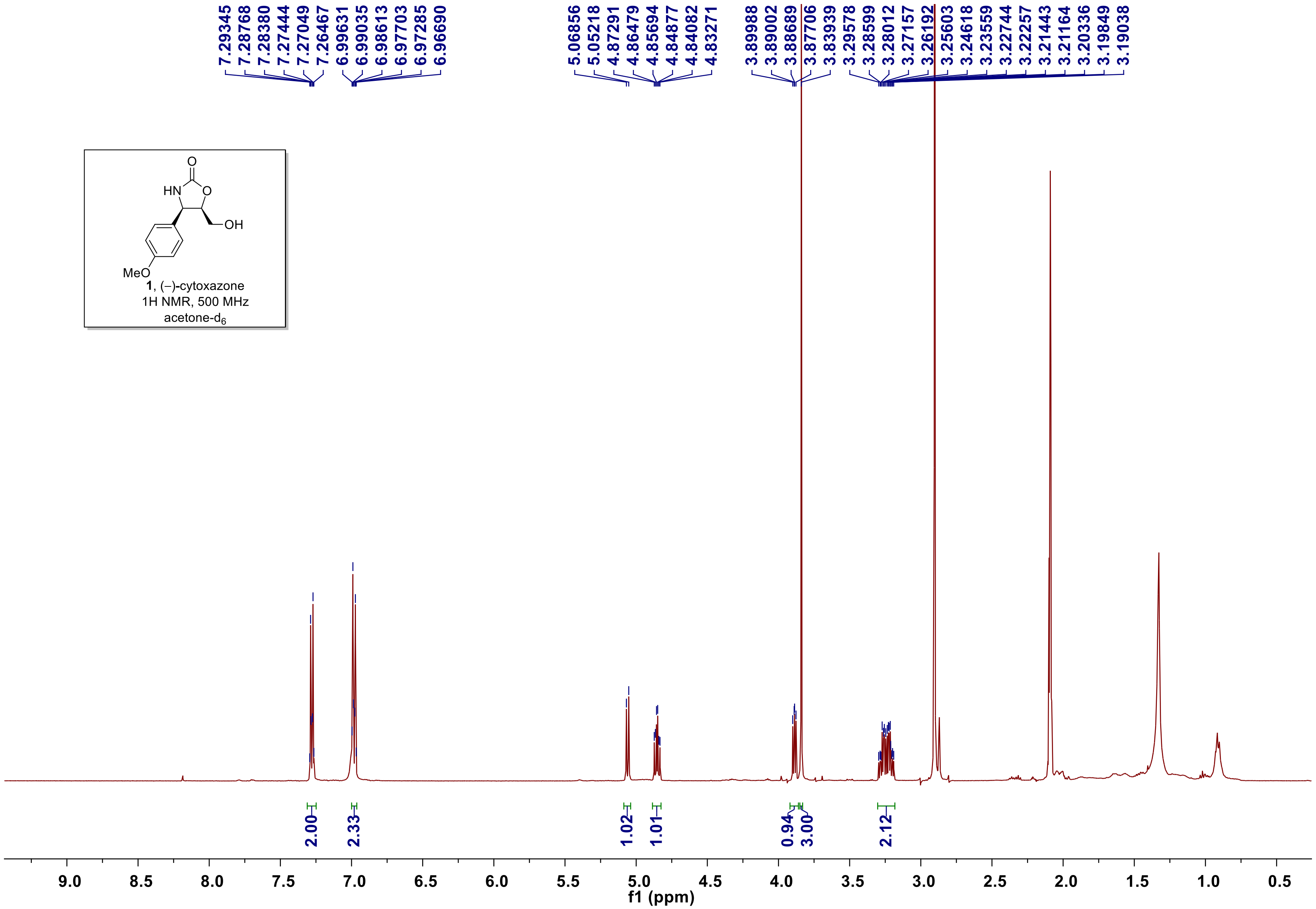
7.29345
7.28768
7.28380
7.27444
7.27049
7.26467
6.99631
6.99035
6.98613
6.97703
6.97285
6.96690
5.06856
5.05218
4.87291
4.86479
4.85694
4.84877
4.84082
4.83271
3.89988
3.89002
3.88689
3.87706
3.83939
3.29578
3.28599
3.28012
3.27157
3.26192
3.25603
3.24618
3.23559
3.22744
3.22257
3.21443
3.21164
3.20336
3.19849
3.19038
HN
O
O
OH
MeO
1, (–)-cytoxazone
1H NMR, 500 MHz
acetone-d6
2.00
2.33
1.02
1.01
0.94
3.00
2.12
9.0 8.5 8.0 7.5 7.0 6.5 6.0 5.5 5.0 4.5 4.0 3.5 3.0 2.5 2.0 1.5 1.0 0.5
f1 (ppm)

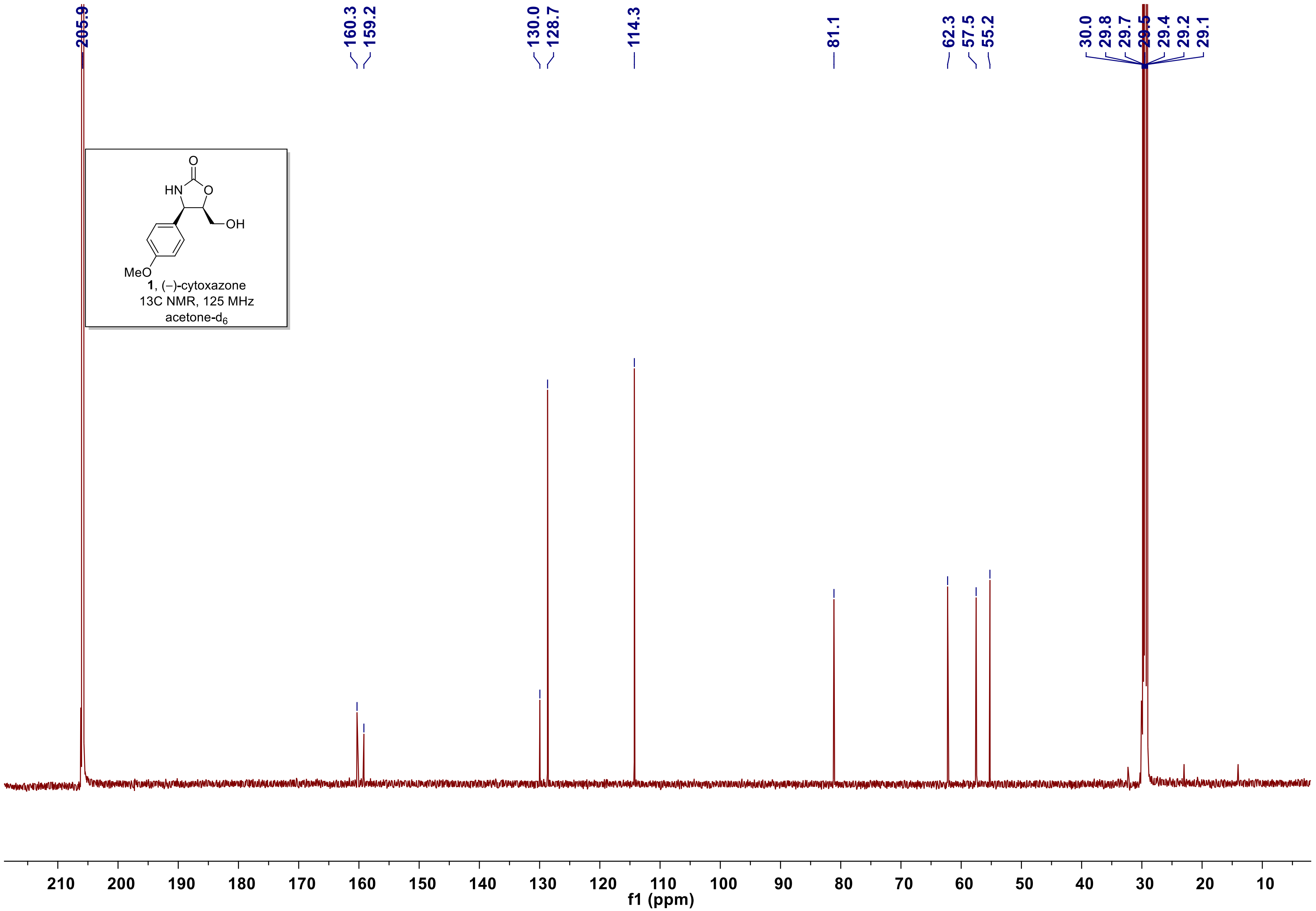
205.9
160.3
159.2
130.0
128.7
114.3
81.1
62.3
57.5
55.2
30.0
29.8
29.7
29.5
29.4
29.2
29.1
O
HN
O
OH
MeO
1, (–)-cytoxazone
13C NMR, 125 MHz
acetone-d6
210 200 190 180 170 160 150 140 130 120 110 100 90 80 70 60 50 40 30 20 10
f1 (ppm)

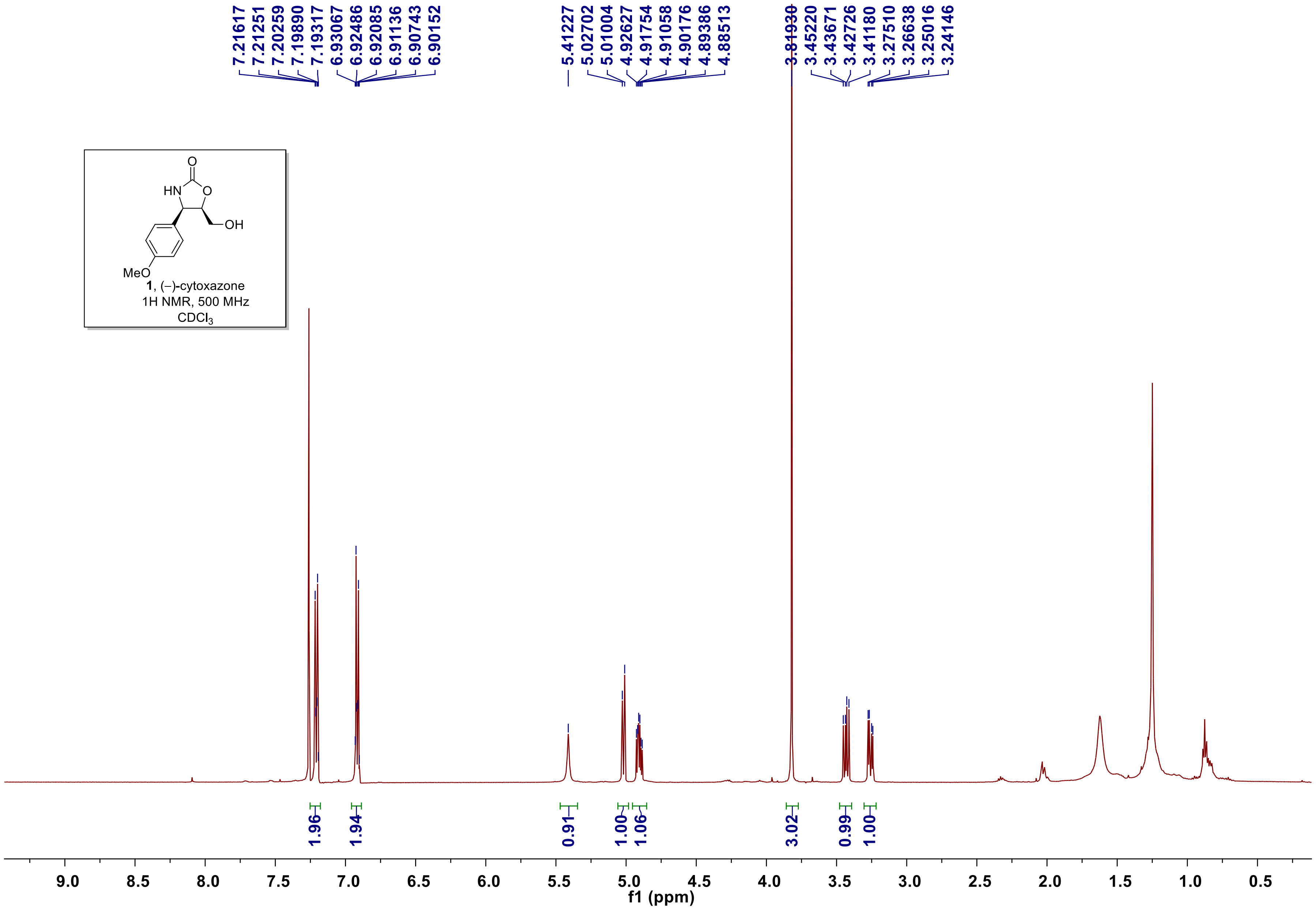
7.21617
7.21251
7.20259
7.19890
7.19317
6.93067
6.92486
6.92085
6.91136
6.90743
6.90152
5.41227
5.02702
5.01004
4.92627
4.91754
4.91058
4.90176
4.89386
4.88513
3.81930
3.45220
3.43671
3.42726
3.41180
3.27510
3.26638
3.25016
3.24146
HN
O
O
OH
MeO
1, (−)-cytoxazone
1H NMR, 500 MHz
CDCl3
1.96
1.94
0.91
1.00
1.06
3.02
0.99
1.00
9.0
8.5
8.0
7.5
7.0
6.5
6.0
5.5
5.0
4.5
4.0
3.5
3.0
2.5
2.0
1.5
1.0
0.5
f1 (ppm)

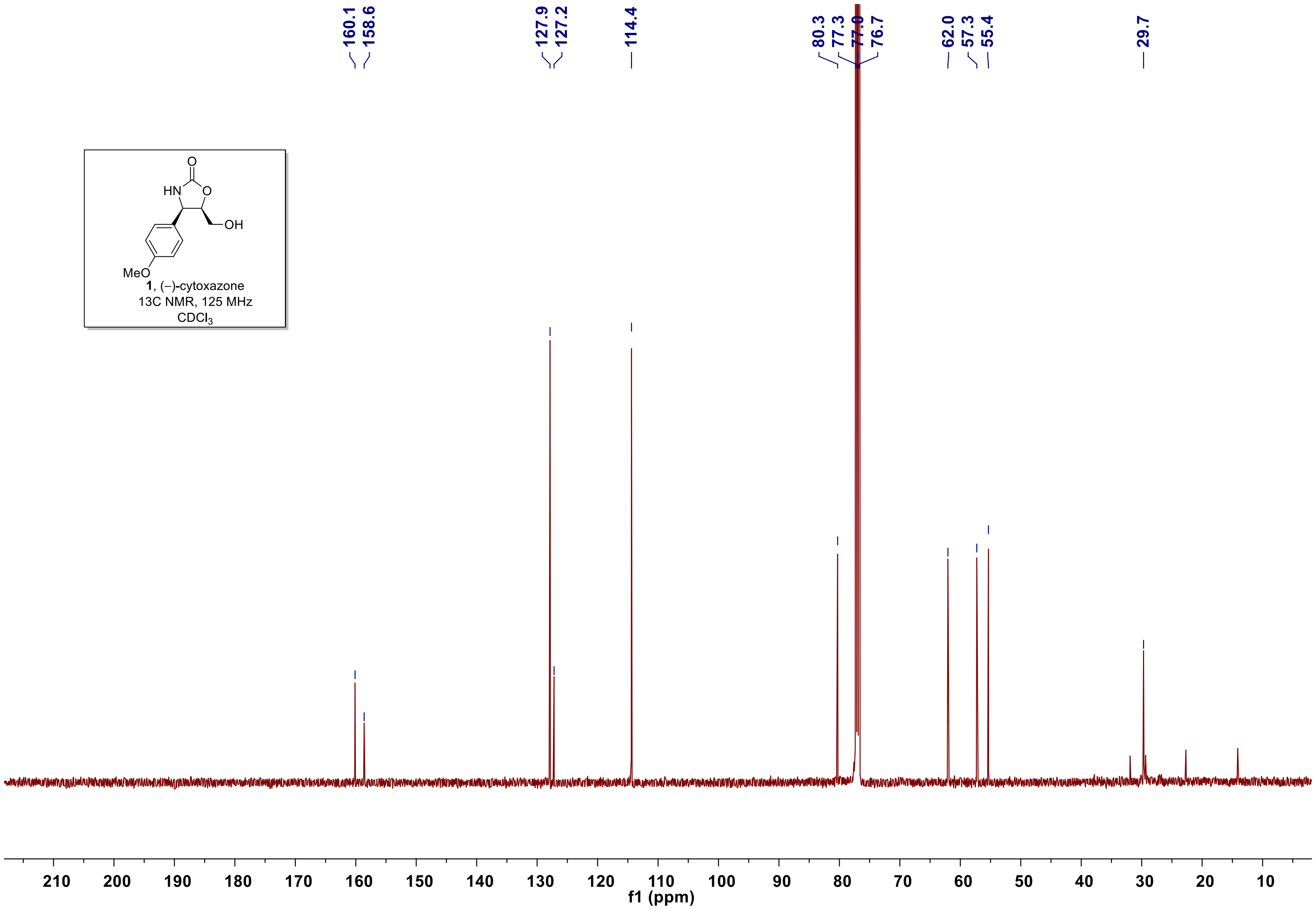
160.1
158.6
127.9
127.2
114.4
80.3
77.3
77.0
76.7
62.0
57.3
55.4
29.7
HN
O
O
OH
MeO
1, (–)-cytoxazone
13C NMR, 125 MHz
$CDCl_3$
210 200 190 180 170 160 150 140 130 120 110 100 90 80 70 60 50 40 30 20 10
f1 (ppm)

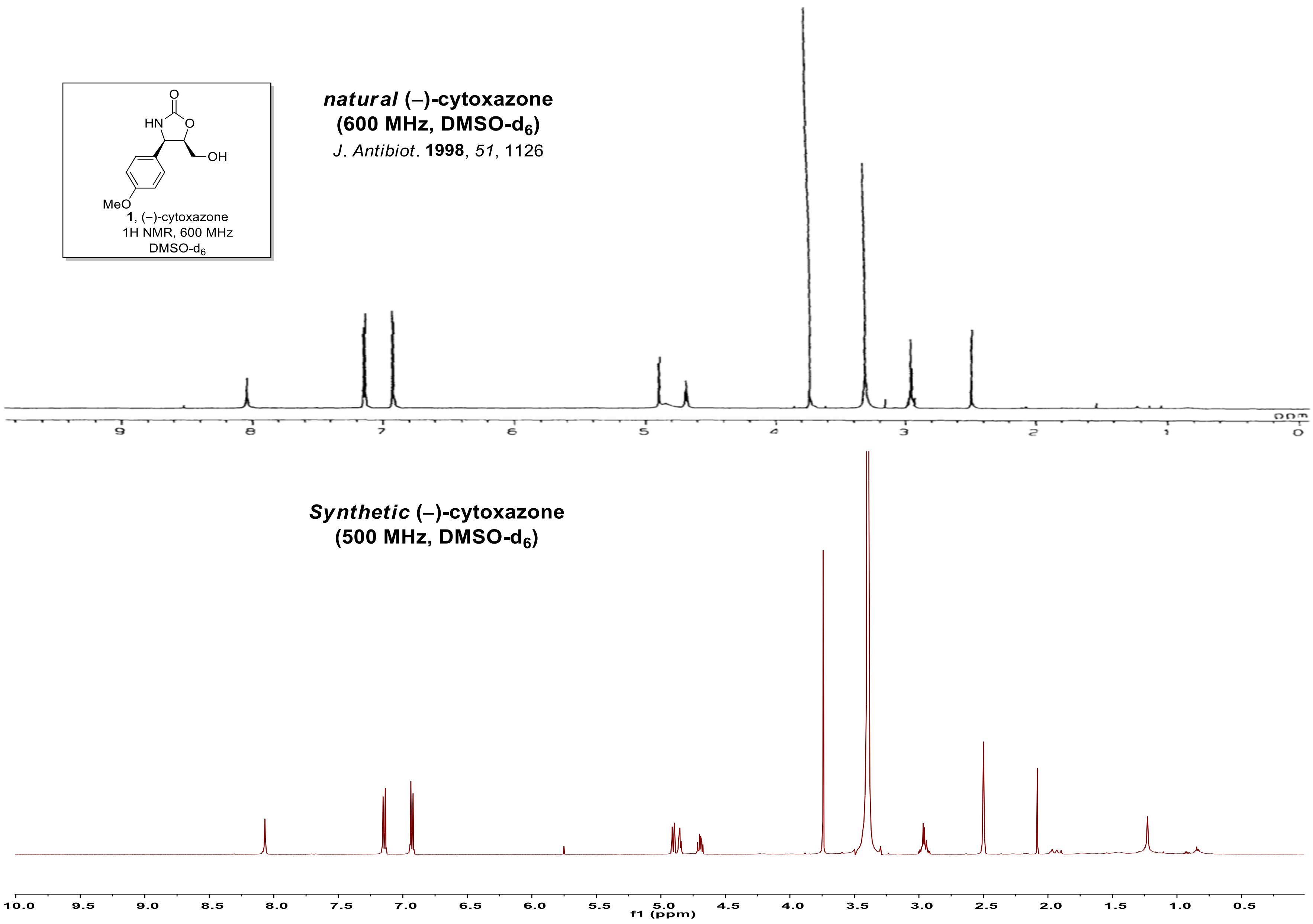
HN
O
O
OH
MeO
1, (–)-cytoxazone
1H NMR, 600 MHz
DMSO-d_6
natural (–)-cytoxazone
(600 MHz, DMSO-d_6)
J. Antibiot. 1998, 51, 1126
ppm
Synthetic (–)-cytoxazone
(500 MHz, DMSO-d_6)
f1 (ppm)

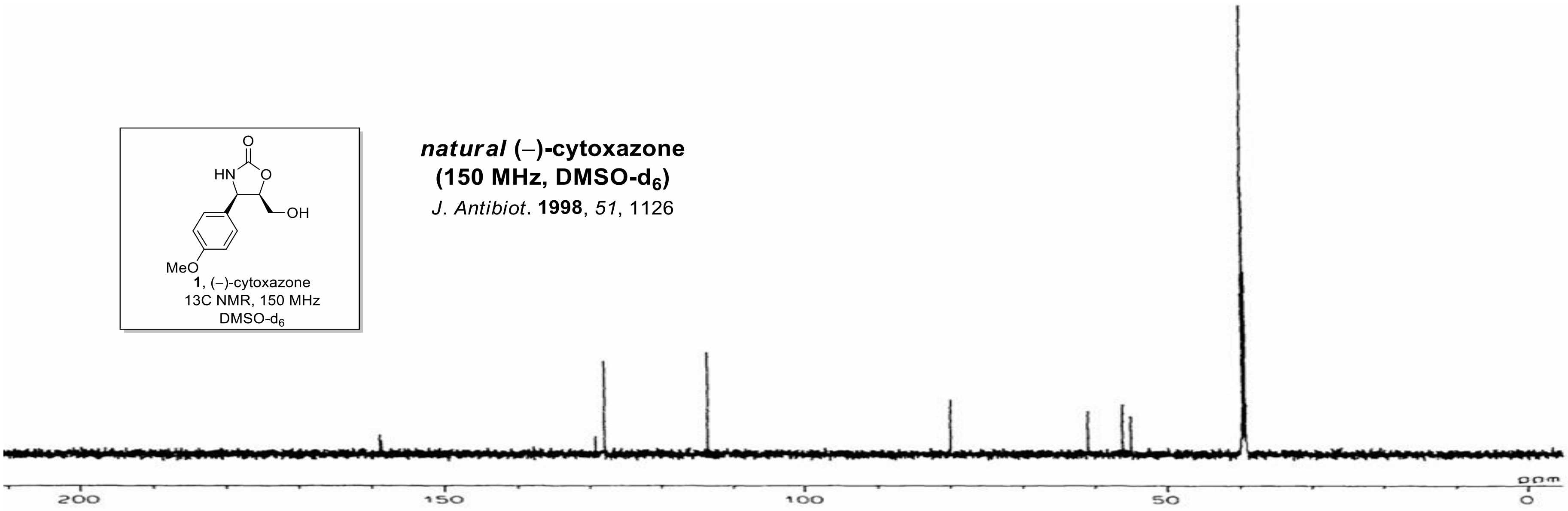

HN
O
O
OH
MeO
1, (–)-cytoxazone
13C NMR, 150 MHz
DMSO-d6
natural (–)-cytoxazone
(150 MHz, DMSO-d6)
J. Antibiot. 1998, 51, 1126
ppm
200
150
100
50
0

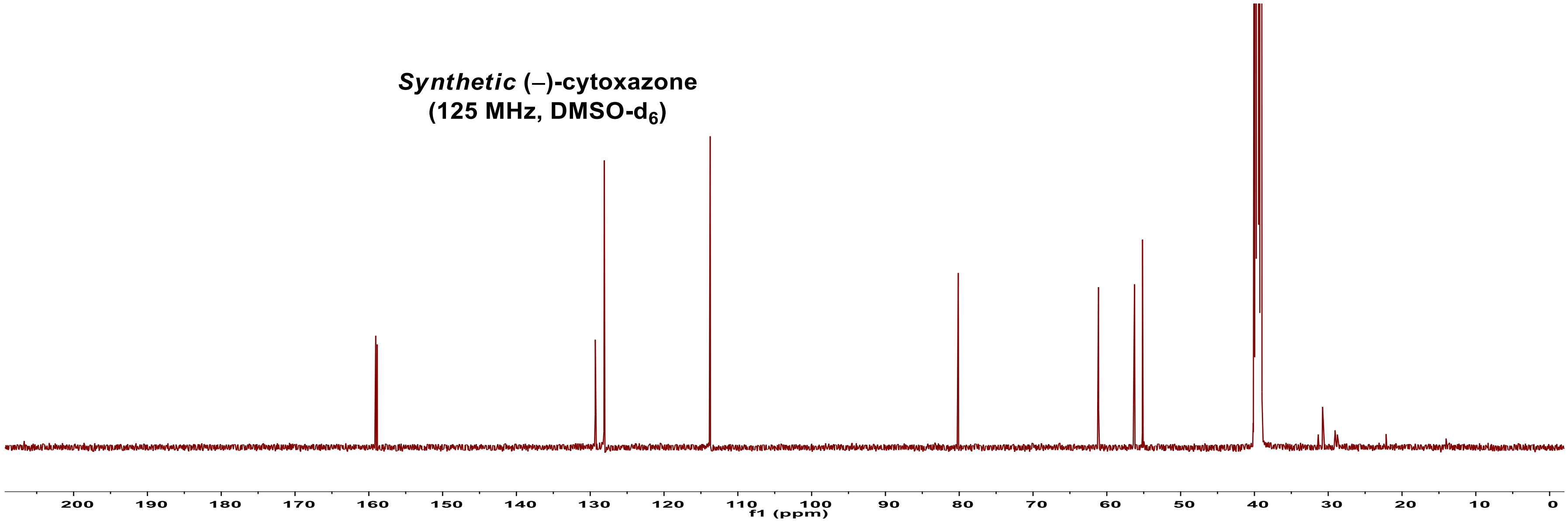

Synthetic (–)-cytoxazone
(125 MHz, DMSO-d6)
200
190
180
170
160
150
140
130
120
110
100
90
80
70
60
50
40
30
20
10
0
f1 (ppm)

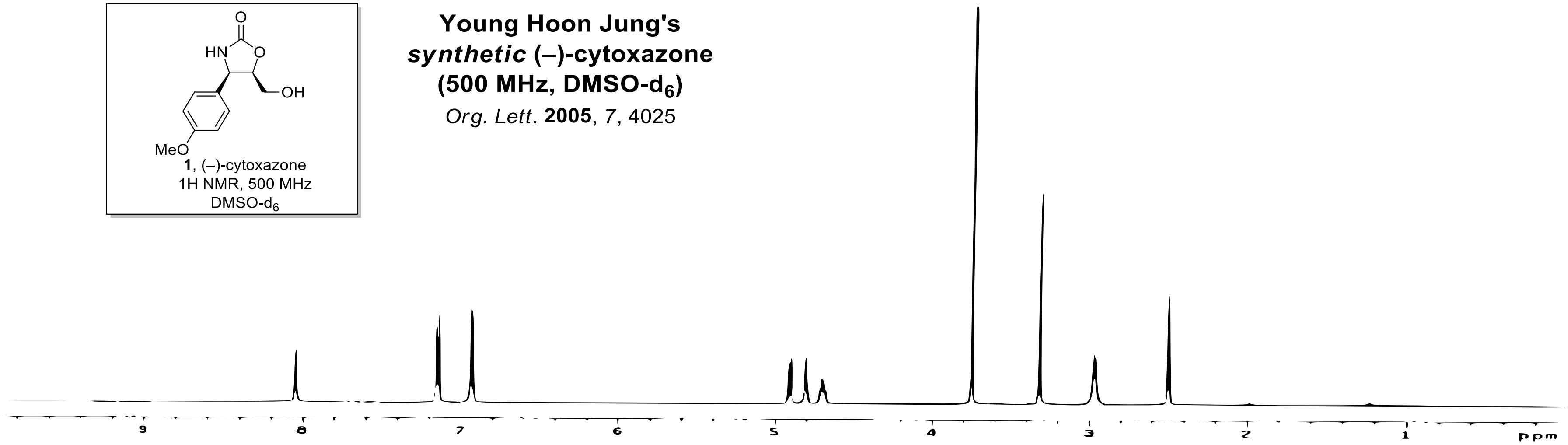
Young Hoon Jung's
synthetic (–)-cytoxazone
(500 MHz, DMSO-d_6)
Org. Lett. **2005**, *7*, 4025
O
HN
O
OH
MeO
1, (–)-cytoxazone
1H NMR, 500 MHz
DMSO-d_6
9
8
7
6
5
4
3
2
1
ppm

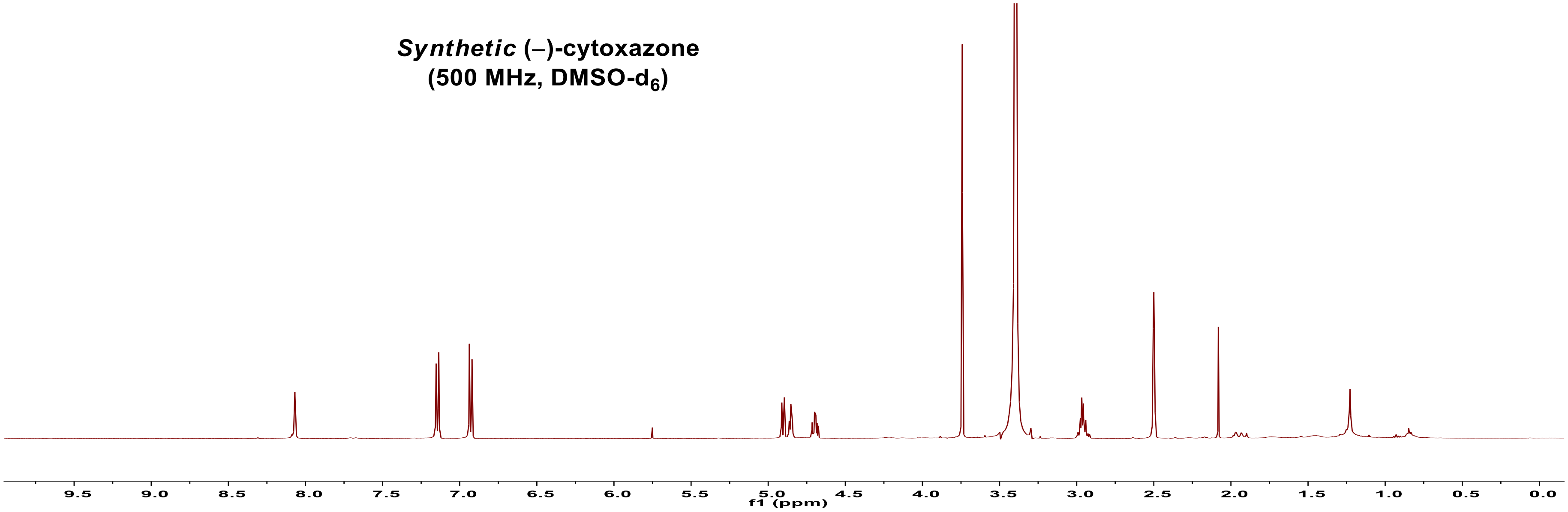
Synthetic (–)-cytoxazone
(500 MHz, DMSO-d_6)
9.5
9.0
8.5
8.0
7.5
7.0
6.5
6.0
5.5
5.0
4.5
4.0
3.5
3.0
2.5
2.0
1.5
1.0
0.5
0.0
f1 (ppm)

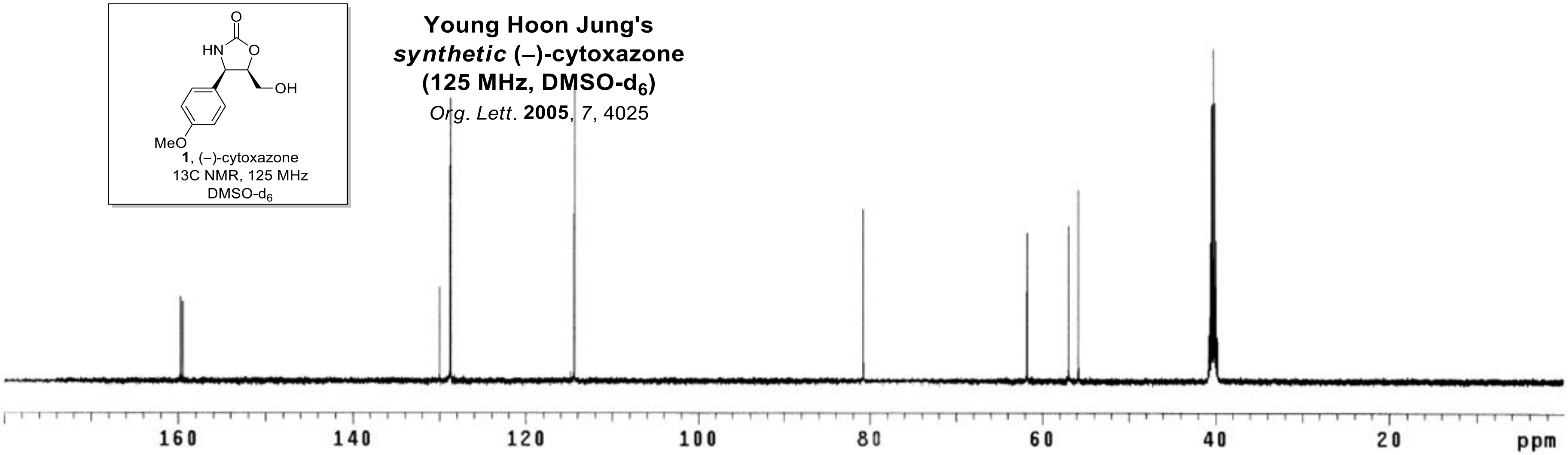

1, (–)-cytoxazone
13C NMR, 125 MHz
DMSO-d_6

Young Hoon Jung's
***synthetic* (–)-cytoxazone**
(125 MHz, DMSO-d_6)
Org. Lett. **2005**, *7*, 4025

***Synthetic* (–)-cytoxazone**
(125 MHz, DMSO-d_6)

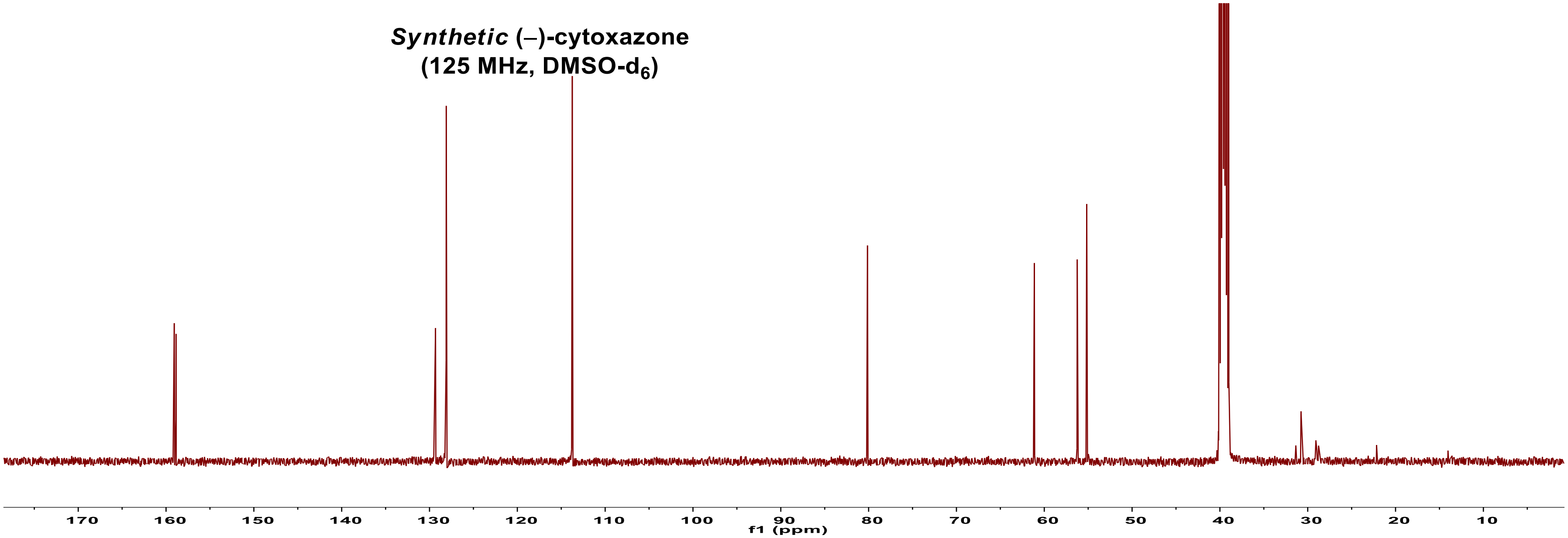

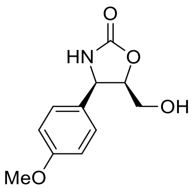

1, (–)-cytoxazone
1H NMR, 500 MHz
DMSO-d_6

Takashi Ohshima and Masakatsu Shibasaki's
***synthetic* (–)-cytoxazone**
(500 MHz, DMSO-d_6)
J. Am. Chem. Soc. **2005**, *127*, 2147

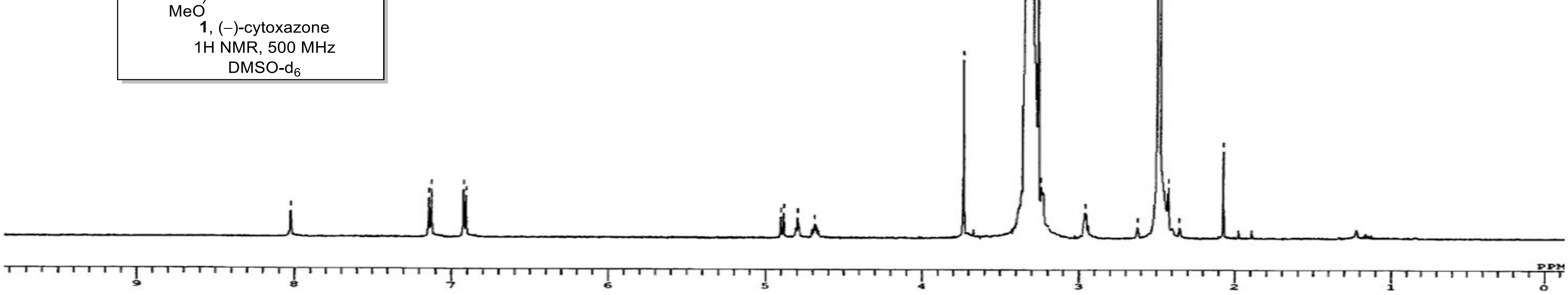

***Synthetic* (–)-cytoxazone**
(500 MHz, DMSO-d_6)

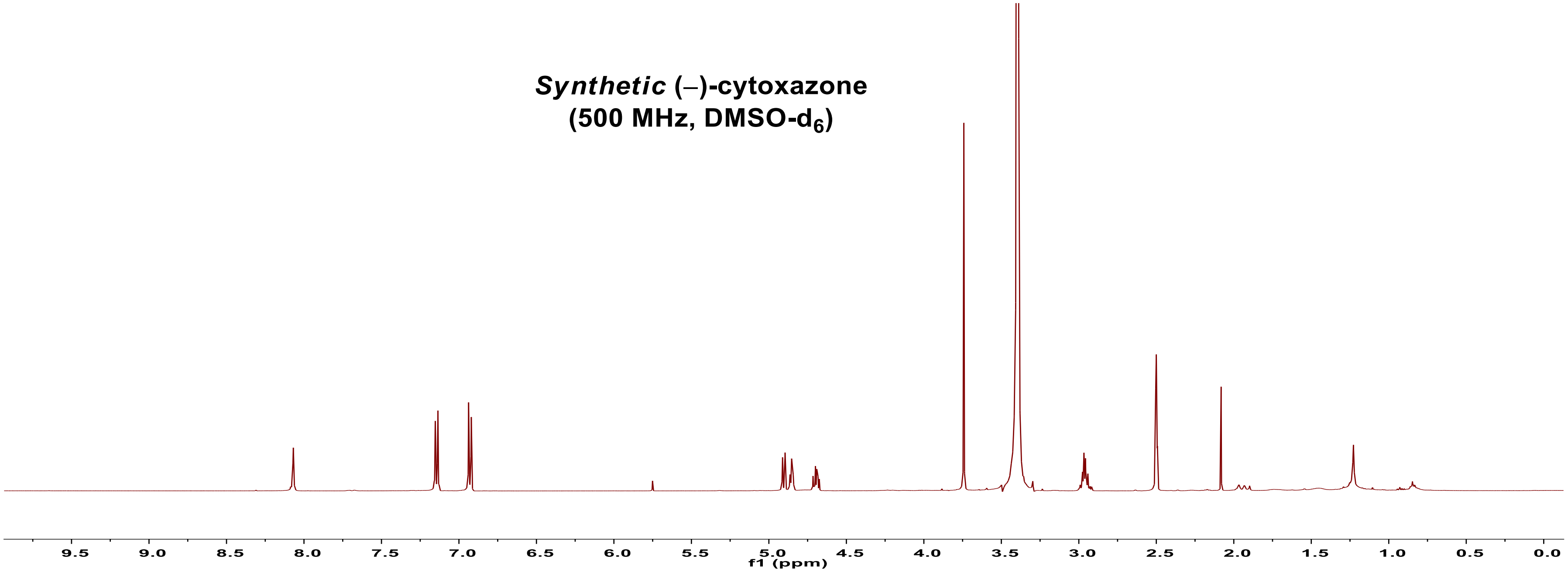

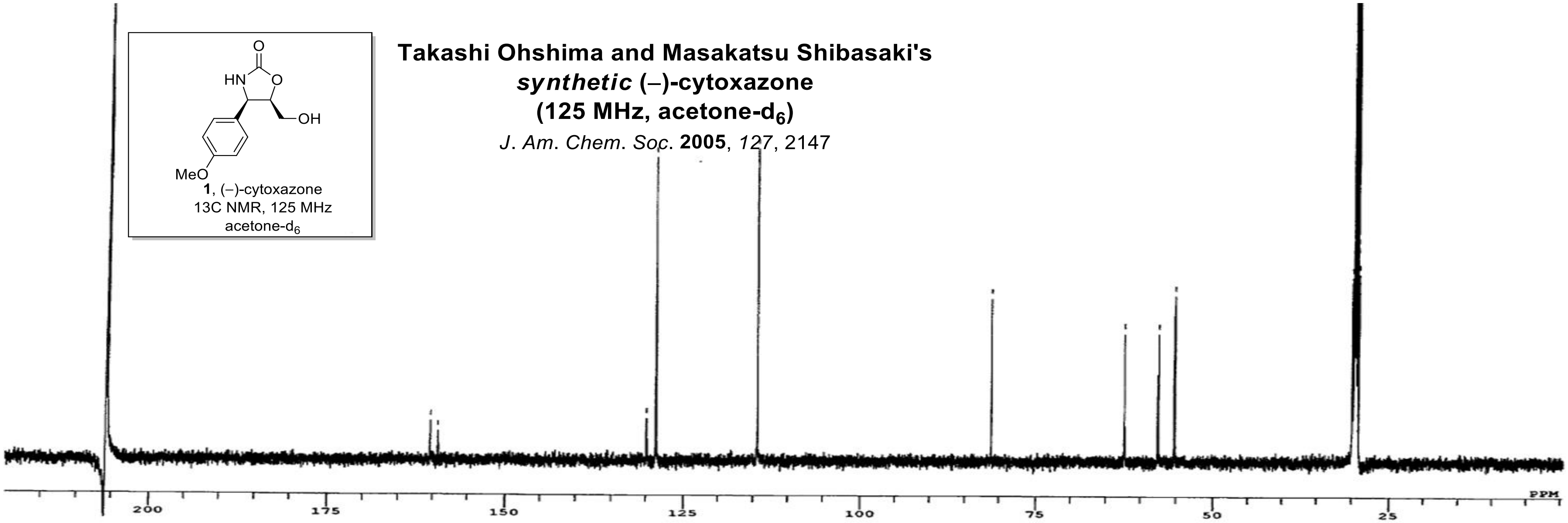
HN
O
O
OH
MeO
1, (–)-cytoxazone
13C NMR, 125 MHz
acetone-d6
Takashi Ohshima and Masakatsu Shibasaki's
synthetic (–)-cytoxazone
(125 MHz, acetone-d6)
J. Am. Chem. Soc. 2005, 127, 2147
200
175
150
125
100
75
50
25
PPM

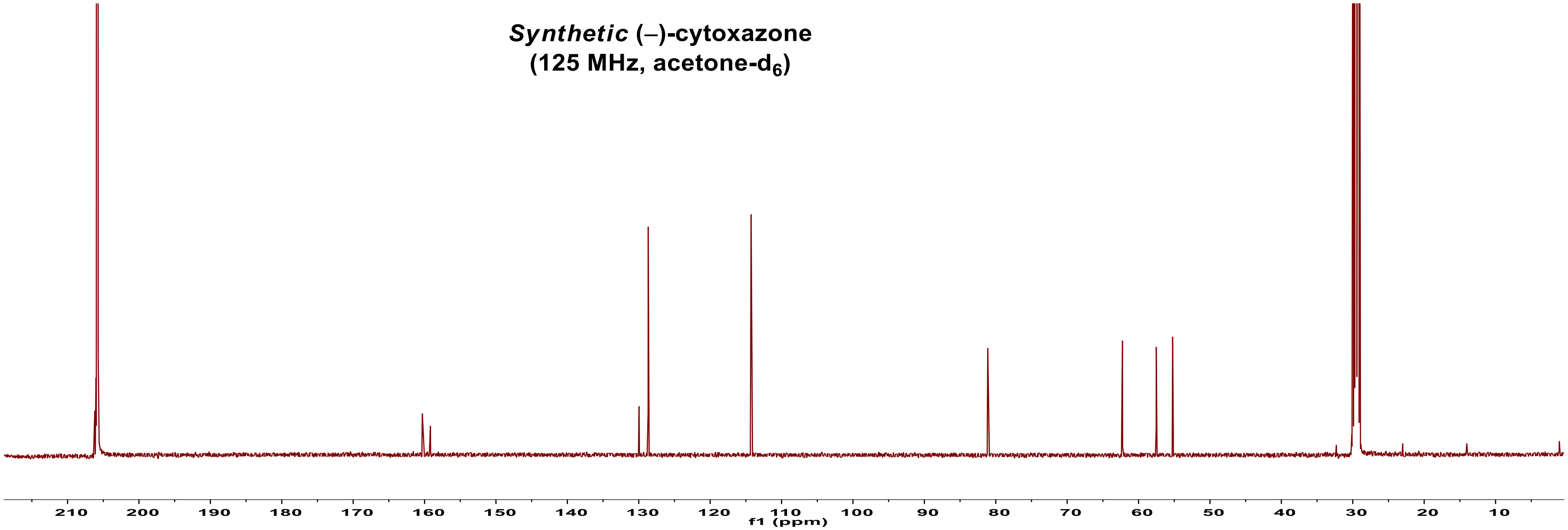
Synthetic (–)-cytoxazone
(125 MHz, acetone-d6)
210
200
190
180
170
160
150
140
130
120
110
100
90
80
70
60
50
40
30
20
10
f1 (ppm)

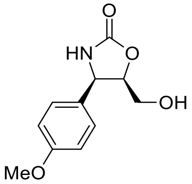

1, (–)-cytoxazone
1H NMR, 300 MHz
acetone-d_6

Vladimir B. Birman's
synthetic **(–)-cytoxazone**
(300 MHz, acetone-d_6)
J. Am. Chem. Soc. **2006**, *128*, 6536

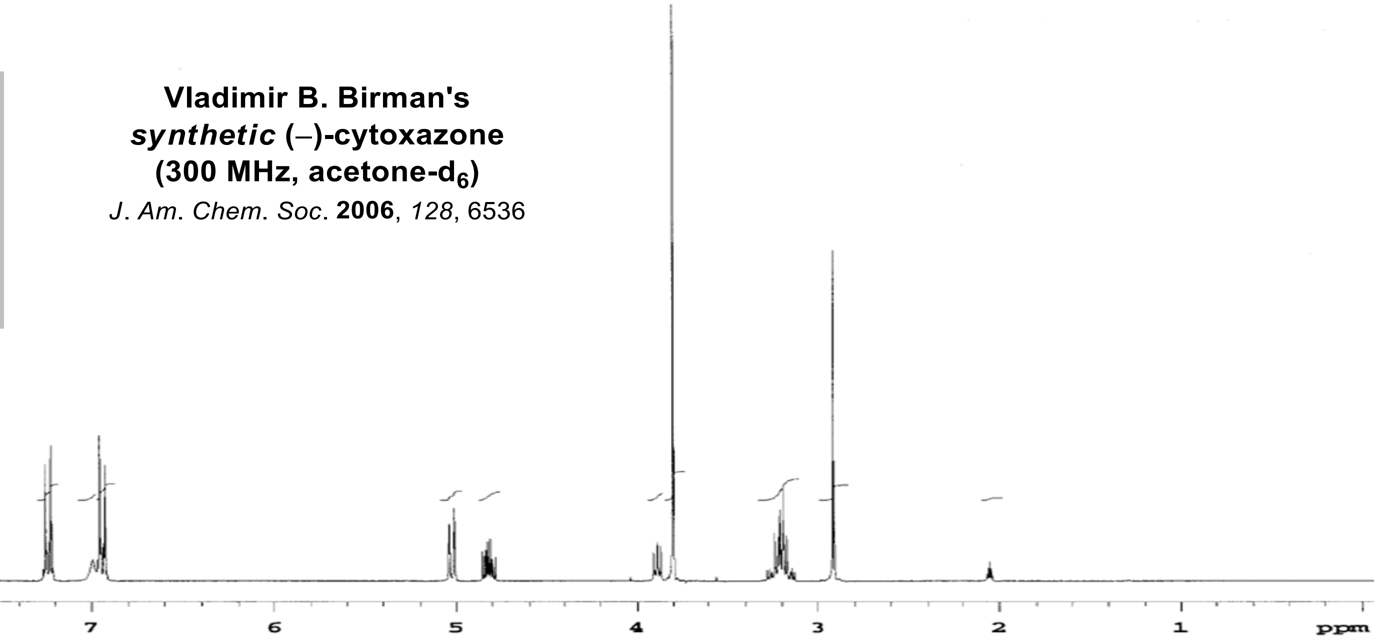

Synthetic **(–)-cytoxazone**
(500 MHz, acetone-d_6)

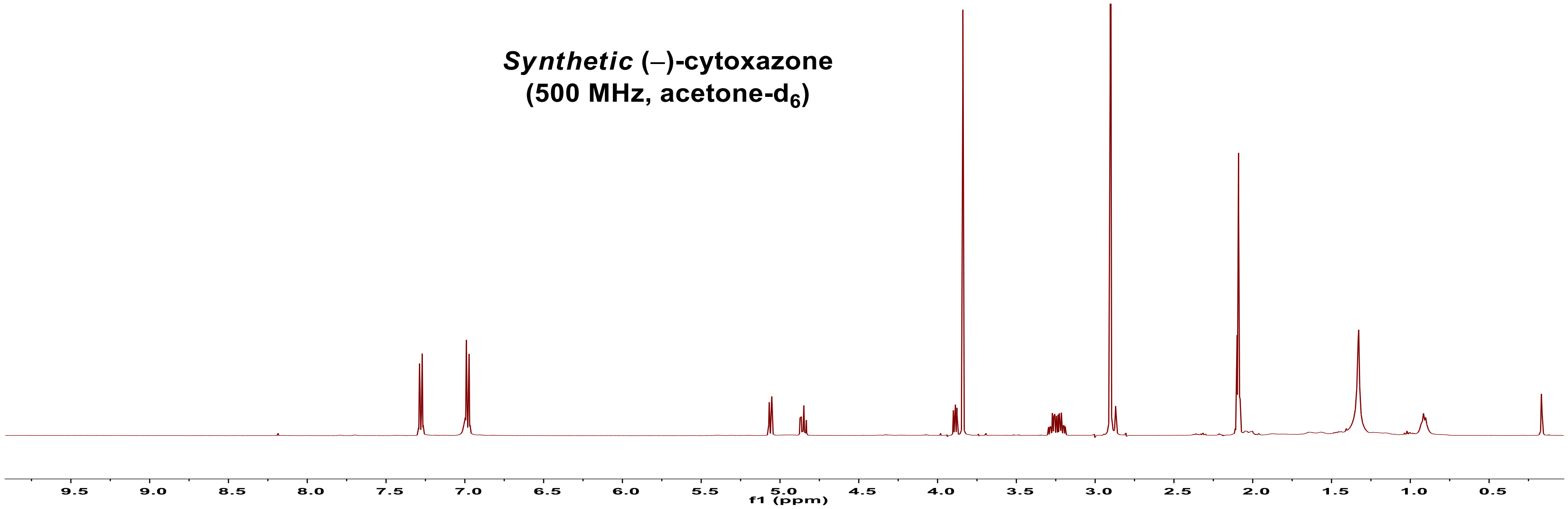

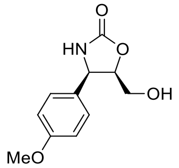

1, (–)-cytoxazone
13C NMR, 75 MHz
acetone-d_6

Vladimir B. Birman's
***synthetic* (–)-cytoxazone**
(75 MHz, acetone-d_6)
J. Am. Chem. Soc. **2006**, *128*, 6536

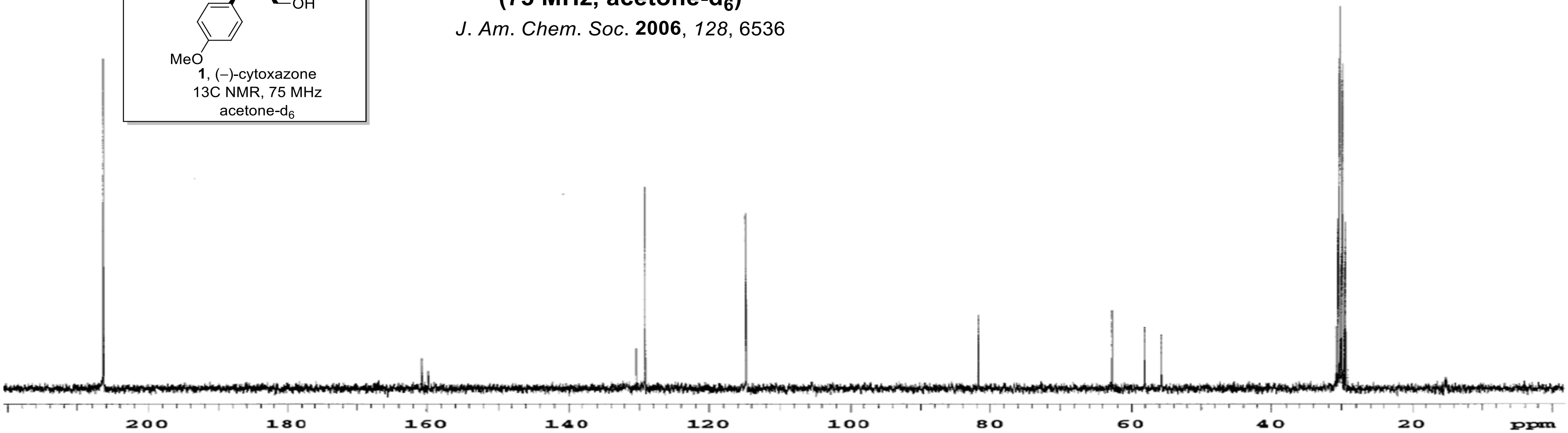

***Synthetic* (–)-cytoxazone**
(125 MHz, acetone-d_6)

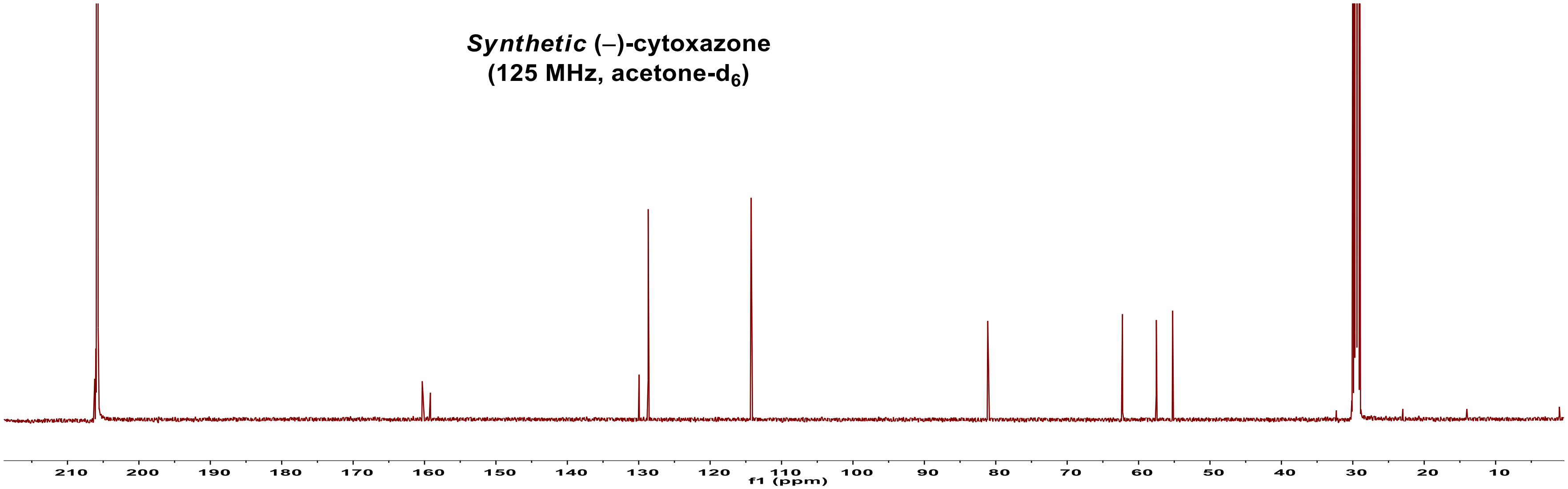

Supplement: Supplementary file 1 [file molecules-26-00597-s001.pdf]
